# Effects of meteorological variability on the burden of musculoskeletal disorders among people aged 55 and above in the United States: a secondary analysis of the Global Burden of Disease Study 2021

**DOI:** 10.3389/fpubh.2026.1630531

**Published:** 2026-03-17

**Authors:** Yingying Zhou, Jiemin Wang, Zhi Xie, Jun Zhang

**Affiliations:** 1 Changsha Center for Disease Control and Prevention, Changsha, China; 2 CÚRAM, Centre for Research in Medical Devices, University of Galway, Galway, Ireland; 3 Department of Orthopedics, The First Affiliated Hospital of Hunan Traditional Chinese Medical College (Hunan Provincial Directly Affiliated Hospital of Traditional Chinese Medicine), Zhuzhou, China

**Keywords:** behavioral risk factor surveillance system, disability-adjusted life years, Global Burden of Disease study 2021, global historical climatology network, meteorological variability effects, musculoskeletal disorders

## Abstract

**Background:**

Older adults in high-income countries face an increasing burden of musculoskeletal (MSK) disorders. Although the belief that meteorological variations influence musculoskeletal conditions is widespread, scientific evidence remains inconclusive. This study aimed to explore these meteorology-health relationships.

**Methods:**

Using publicly available data from the Global Historical Climatology Network Daily, Behavioral Risk Factor Surveillance System and the Global Burden of Disease Study 2021, we investigated associations between inter-annual meteorological factors variability (temperature, humidity, wind speed, barometric pressure) and musculoskeletal burdens, measured by disability-adjusted life years, among adults aged ≥55 in the United States. Penalized Generalized Additive Models were employed to investigate the potential associations for rheumatoid arthritis, osteoarthritis, low back pain, neck pain, gout, and other musculoskeletal diseases. State-level Socio-demographic Index and behavioral indicators (obesity prevalence and smoking rate) were included as covariables, and population density and healthcare access indices were incorporated in sensitivity analyses.

**Results:**

Musculoskeletal burdens among older U.S. adults substantially exceeded global averages (6,205.82 vs. 4,837.46 per 100,000), particularly for other musculoskeletal disorders in females and gout in males. Burdens generally increased from 2013 to 2021, except for rheumatoid arthritis and low back pain. Incorporating meteorological terms, jointly accounted for SDI, behavioral risk factors and residual temporal trends, improved model performance and revealed non-linear exposure-response patterns with potential thresholds. Inter-annual variations in temperature and humidity consistently exhibited significant associations with disease burden, while wind speed and barometric pressure also associated with burden. Sex-based heterogeneity emerged, most prominently for gout and rheumatoid arthritis.

**Conclusions:**

Our findings highlight consistent population-level associations between inter-annual meteorological variability and MSK burdens among older U.S. adults. These associations may reflect direct environmental influences as well as broader behavioral, infrastructural, and contextual contexts, underscoring the need for context-sensitive and sex-specific public-health strategies for aging populations.

## 1 Background

Musculoskeletal (MSK) disorders are among the leading causes of morbidity and long-term disability worldwide (1). Common characteristics of MSK disorders involve chronic pain, reduced flexibility, impaired function, and diminished work capacity, posing a great potential to compromise quality of life and placing significant economic burdens on individuals and healthcare systems (2, 3).

Findings from Global Burden of Diseases, Injuries, and Risk Factors Study (GBD) indicated that MSK disorders largely affect older adults, with the peak age of onset being 50 and 54 years (3–5). Additionally, MSK disorders share several risk factors common to other non-communicable diseases, including aging, obesity, poor nutrition, and a sedentary lifestyle (2, 6, 7). The co-occurrence with serious chronic conditions can worsen health outcomes and intensify financial strains on healthcare, especially in an aging global population (6). As such, understanding both the individual and contextual determinants that contribute to the MSK disease burden should be a critical public health priority.

Globally, the prevalence and associated health burden of MSK disorders vary substantially by geographic region, and diagnostic category, with high-income countries experiencing a disproportionately greater impact (7–9). A female-biased trend in the overall burden of MSK was also documented, suggesting that sex/gender may play a role in MSK development (1, 3, 4, 9). For example, females experience more low back pain, whereas gout is typically seen more often in males (10, 11).

Beyond these sociodemographic contributors, a growing body of research has highlighted potential links between meteorological factors, such as temperature, barometric pressure, humidity and precipitation, and MSK symptoms (12–15). Notably, gout stands out for its sensitivity to environmental triggers: temperature and humidity can alter physiological regulation of serum urate levels (12, 16, 17). In other MSK conditions, shifts in weather coinciding with variations in joint pain and stiffness is supported by theories that barometric pressure could affect joint spaces, temperature might modulate inflammatory mediators, and behavioral or emotional changes linked to seasonality could exacerbate pain (16, 18, 19). Nevertheless, more recent findings question the consistency of these weather-pain links (17, 20), citing limitations including restricted disease coverage, short study durations, wide geographic variability, or reliance on self-reported meteorological exposures. As a result, epidemiological evidence regarding direct meteorological influences on MSK disorders remains inconclusive. It is noteworthy that these reported associations may not necessarily reflect direct biological effects of specific atmospheric variables alone. At the population level, meteorological indicators may also function as contextual proxies for broader behavioral, environmental, and infrastructural conditions, such as seasonal changes of physical activity, indoor heating and cooling practices, air pollution, and access to healthcare (21–26), which themselves are socially patterned and often exhibit gendered distributions.

In the present study, with a focus on the 55-and-older age group, we are trying to address the research gap by leveraging three high-quality, nationally representative data sources. First, GBD 2021 estimates to evaluate the MSK disorder burden across the United States. Second, inter-annual meteorological data were obtained from the Global Historical Climatology Network-Daily (GHCN-D) Version 3 to characterize annual variations of temperature, humidity, barometric pressure and wind speed. Third, state-level behavioral risk and healthcare access indicators were derived from the Behavioral Risk Factor Surveillance System (BRFSS), providing standardized estimates of obesity prevalence, smoking rate, and healthcare access patterns. The GBD data offer a comprehensive, standardized methodology for comparing disease metrics over time and across geographic regions (27). To facilitate systematic evaluation and comparison of the worldwide burden, GBD 2021 classified MSK disorders into six major categories: rheumatoid arthritis, osteoarthritis, low back pain, neck pain, gout, and other musculoskeletal diseases.

We chose to study the United States (U.S.) for several reasons. First, MSK disorders increasingly strain the country's healthcare system and economy, with one of the highest age-standardized prevalence rates worldwide (9, 28). Second, the Global Historical Climatology Network (GHCN), which is supported by U.S. National Centers for Environmental Information, provides detailed meteorological indicators that are uniquely available for U.S. territories. Third, the broad geographic expanse of contiguous U.S. captures substantial meteorological variation while relatively narrowing confounding factors associated with economic development, demographics, and healthcare resources. We specifically limit our modeling analysis to the contiguous 48 states and the District of Columbia (hereafter referred to as the “49 continental states”), excluding Alaska, Hawaii, Fed States of Micronesia, and Puerto Rico, to further minimize the effects of extreme outlier weather conditions or socioeconomic contexts, thus allowing a more reliable assessment of potential meteorology-health associations. Moreover, we adjusted for key sociodemographic and structural factors, including the Socio-demographic Index (SDI), age-specific population estimates, and state-level behavioral indicators, to account for potential confounding and effect modification in observed associations between inter-annual meteorological variability and MSK burden.

To elucidate these relationships, we employ two complementary statistical approaches. First, we use Generalized Linear Models (GLMs) to identify broad associations between meteorological conditions and MSK burden. Then, we apply penalized Generalized Additive Models (GAMs) to detect non-linear patterns and complex interactions. By integrating population-level disease estimates from GBD 2021, systematically curated meteorology observations from GHCN-D, and state-level behavioral indicators from BRFSS, together with sophisticated analytic techniques, this research aims to explore how weather variability influences MSK disorders among older adults. Rather than inferring direct causality, our findings are intended to enhance understanding of population-level meteorology-MSK associations and to support context-sensitive public health planning and preventive strategies in aging societies.

## 2 Method

### 2.1 Data sources

This is an ecological study based on aggregated, population-level data. The MSK burden estimates examined in this study were obtained from the Global Burden of Diseases, Injuries, and Risk Factors Study 2021 (GBD 2021), accessed via the Results Exchange Tools (https://vizhub.healthdata.org/gbd-results/). Detailed information regarding data resources, collection methods, statistical analysis, and modeling strategies implemented by GBD 2021, as well as initiatives to improve data interpretation, are described elsewhere (27, 29). GBD 2021 provides annual, standardized estimates of disease burden for multiple geographic regions and countries. In the United States, these data are further disaggregated into 53 sub-national units (states). In addition, GBD 2021 supplies annually estimated Socio-demographic Index (SDI) and age-specific population data (for both males and females in all ages, and specifically those aged 55 and above) for each included location.

State-level data on obesity prevalence, current smoking rate, and healthcare access indicators were obtained from the Behavioral Risk Factor Surveillance System (BRFSS: https://www.cdc.gov/brfss/brfssprevalence/index.html) with available public estimates. For state-and-year records with missing values (New Jersey in 2019 and Florida in 2021), all variables were imputed using nearest-neighbor filling across contiguous years within each state-sex stratum.

The principal meteorological variables were extracted from the Global Historical Climatology Network–Daily (GHCN-D) Version 3.31 (https://www.ncei.noaa.gov/access/search/data-search/global-hourly). GHCN-D is a comprehensive dataset maintained by the National Centers for Environmental Information (NCEI) under the National Oceanic and Atmospheric Administration (NOAA). It compiles daily weather observations from thousands of land-based stations worldwide, all subjected to rigorous quality control procedures, including checks for outliers and internal consistency, ensuring reliable, standardized data (30). GHCN-D has been used in many studies investigating the links between meteorological exposures and health or ecological outcomes, owing to its extensive spatial coverage, historical depth, and consistent data quality (31–33). To derive the annual estimates of the meteorological variables of interest for each state, external multi-polygons of the United States (including 59 spatial unites) was imported from the National Weather Service (NOAA/NWS) (https://www.weather.gov/gis/USStates). These geographic boundary files enabled us to delineate state borders accurately and calculate state-level annual summaries of the daily meteorological data.

### 2.2 Statistical software

Joinpoint analysis conducted by Joinpoint Trend Analysis Software (Desktop version 5.3.0, https://surveillance.cancer.gov/joinpoint/). Other involved data analyses and visualizations were realized using R version 4.4.2. Tables and figures were formatted by Microsoft Word for MAC Version 16.94 (25020927) and Microsoft PowerPoint for Mac Version 16.95.1 (25031528). Unless otherwise specified, statistical significance was defined at the 0.05 level. We complied with the Standardized Reporting of Burden of Disease studies statement (the STROBOD statement) when presenting secondary analysis results (34). A corresponding checklist is provided in Supplementary Section 1.

### 2.3 Data preprocessing

#### 2.3.1 The burden of musculoskeletal (MSK) disorders

GBD studies apply a four-level hierarchical structure of cause categories, ranking from the most general to the most specific. MSK disorders are listed as a second-level cause and are subdivided at the third level into rheumatoid arthritis, osteoarthritis, low back pain, neck pain, gout, and other musculoskeletal diseases (see Supplementary Section 3) (35). We extracted three metrics (number, percent, rate) of Disability-Adjusted Life-Years (DALYs), along with 95% Uncertainty Intervals (95% UI), for the level 2 and 3 causes across two default age groups (all ages and 55 years and older). MSK burden as measured by DALYs captures the cumulative impact of recurrent symptoms, functional impairment, and healthcare use over time.

Focusing on 2021, we first calculated the disorder-specific percentages via the percent of each MSK subtype (the level 3 cause) diving the percent of the total MSK burden (the level 2 cause). And the rates (measured as years per 100,000 people) of DALYs attributed to total MSK disorders and its six subgroups were compared among four geographic units (Global, High-SDI, High-income North America, and the United States of America). This comparison aimed to contextualize the MSK burden in the United States relative to the global level and other developed regions. Joinpoint analysis was then performed to identify significant temporal changes in DALY rates over the study period (2013–2021). Based on the data characteristics, we allowed up to two joinpoints to capture major trend shifts. The Average Annual Percent Change (AAPC) was subsequently mapped to succinctly convey overall trends across states, while capturing short-term fluctuations during the analyzed years (4).

#### 2.3.2 The inter-annual meteorological variability

We obtained original meteorological data from the U.S. Historical Climatology Network (file “ghcnd_hcn.tar.gz”) for the period 1 January 1990 to 31 December 2021. Daily records underwent NOAA/NCEI Qualification Control (QC); invalid/missing daily values were removed, and annual summaries were computed from valid observations only (further details appear in Supplementary Section 4). To minimize the effects of extreme outlier weather conditions or socioeconomic contexts, 49 continental states (the 48 mainland states plus the District of Columbia, excluding Alaska, Hawaii, Fed States of Micronesia, Puerto Rico) were specifically incorporated into our analysis. Using the *gstat, sp*, and *raster* packages in conjunction with a U.S. multi-polygons shapefile (imported via the *sf* package), we applied Inverse Distance Weighting (IDW) interpolation over the continental domain to obtain continuous annual fields and derived state-level estimates by zonal averaging those fields within state polygons. Because the yearly mean temperatures and relative humidity levels were the primary meteorological variables of interest in our study, we retained only records with valid values for both variables. Consequently, the study period was shortened to 2013 to 2021 phase (detailed in Supplementary Section 4). There were finally six meteorological variables included in the modeling. They were specified as follows:

- TRAN_ANN: Annual maximum temperature range
- MEAN_TAVG: Annual average daily temperature
- RRAV_ANN: Annual maximum relative humidity range
- MEAN_RRAV: Annual average daily relative humidity
- MEAN_ASTP: Annual average barometric pressure
- MEAN_AWND: Annual average wind speed

The six meteorological variables were constructed at the annual scale to ensure temporal alignment with yearly DALY outcomes. We included both annual mean indicators (MEAN_TAVG, MEAN_RRAV, MEAN_ASTP, MEAN_AWND) and annual variability indicators (TRAN_ANN and RRAV_ANN) to capture complementary dimensions of long-term regional meteorological exposure. Annual means reflect sustained background environmental conditions, whereas annual range metrics characterize the magnitude of intra-annual variability. Specially, annual mean wind speed (MEAN_AWND) and atmospheric pressure (MEAN_ASTP) were treated as background control indicators capturing stable geographic and infrastructural conditions at the state level, using annual averages as coarse but temporally stable summaries to adjust for broad environmental heterogeneity.

State-level maps were used to illustrate the spatial heterogeneity of the six annual meteorological indicators in 2021, and AAPC was calculated as a descriptive check of their long-term temporal stability (Supplementary Section 5).

#### 2.3.3 Sociodemographic, behavioral factors, and healthcare access

Both SDI values and the population data were obtained directly from the GBD study. To calculate the age- and sex-specific population density of adults aged 55 and over at the state level, we divided the target populations in each state by its geographic area, derived from the U.S. multi-polygon shapefile using the *sf* package in R.

Seven publicly available annual estimates of and healthcare access, which are stratified by state, sex, and calendar year, were extracted for all study years form BRFSS (see Supplementary Section 2). Two variables were used as behavioral and metabolic confounders: obesity prevalence and smoking rate. To reduce dimensionality and potential collinearity among five healthcare access indicators, we applied principal component analysis (PCA) to the five access measures. The first two principal components were retained: PC1 explaining 48.1% and PC2 23.5% of variance.

### 2.4 Modeling strategy

#### 2.4.1 Preparation

To provide an exploratory overview of unadjusted relationships, we firstly examined the bivariate correlations between meteorological variables and the national-level MSK disease burden rates (DALYs) over time. In addition, we evaluated the correlative relationships between the AAPCs of the meteorological variables and the AAPCs of the MSK disease burden rates. These bivariate patterns are presented for descriptive purposes only (see Supplementary Section 8), as they do not account for socioeconomic, behavioral, or healthcare-related confounding. All inferential interpretations are therefore based on the multivariable GAM results described below.

To investigate the adjusted associations between meteorological factors and MSK disease burden, we employed Generalized Linear Model (GLMs) and Generalized Additive Model (GAMs) using the state-level MSK disease burden data (DALYs) as the dependent variable for both sexes combined, as well as for males and females separately. The models were adjusted for SDI, age- and sex-specific population density, obesity prevalence, smoking rate, and healthcare-access principal components where applicable to account for socio-economic, demographic, and behavioral factors that may influence MSK disease burden. All independent variables were rescaled before entering the models, which are detailed in Supplementary Section 4.

#### 2.4.2 Main model specifications

GLMs (via *stats* package) build standard linear regression framework by allowing the dependent variable to follow a range of distributions with a link function. In our study, we used a Gaussian family (normal) link function. We specified two GLMs as:

**Model A:** DALYs ~ SDI

**Model B:** DALYs ~ SDI + Meteorological factors

While GLMs are relatively straightforward to interpret, they impose a strictly linear relationship between predictors and the outcome, which may be too restrictive if the underlying relationships are more complex or non-linear. GAMs extend GLMs by allowing smooth, flexible functions of the predictors rather than strictly linear terms. These smooth functions can capture non-linear and more complex patterns in the data. Model C is specified as:

**Model C:** DALYs ~ SDI + s (Meteorological factors)

In addition to SDI, we further incorporated state-level obesity prevalence and current smoking rate as behavioral and metabolic confounders, given their well-established roles in the etiology and progression of musculoskeletal disorders. These variables were entered as linear covariates in GAM full models (Model D). In addition, smooth terms can be included for time (years) to account for unobserved temporal trends. The full model is specified as follows:

**Model D:** DALYs ~ SDI + Population Density + s (Meteorological factors) + s (Years) + Obesity Prevalence + Smoking Rate

#### 2.4.3 Meteorological factors

We validated our models through multicollinearity assessment, alternative model specifications, and comparisons of model fit. Multicollinearity among linear parametric covariates was evaluated using Variance Inflation Factor (VIF), with a threshold of VIF < 5 indicating acceptable collinearity (see Supplementary Table 3). To further address potential multicollinearity in GAMs (fitted with the *mgcv* package), we utilized penalized cubic regression splines with double penalties [bs = “cr”, m = c(2, 2)], which allow both smoothness control and shrinkage of entire smooth terms. Smoothness selection was guided by restricted maximum likelihood (REML), and redundant terms were automatically penalized using the *select* = *TRUE* option (shrinking toward zero), thereby stabilizing estimates and reducing overfitting. Model fit was further checked by comparing alternative models (Model A–D) using Explained Deviance (R^2^), Residual Variance (Sigma^2^), Maximum Likelihood (ML), and Akaike Information Criterion (AIC). Thus, the adopted framework balances flexibility in capturing genuine non-linearity with built-in penalties that shrink toward simpler forms when appropriate.

We reported GAM smooth-term estimates as Estimated Degrees of Freedom (Edf), Reference Degrees of Freedom (Ref.df), and *p-values*. A statistically significant smooth (*p* < 0.05) indicates that the predictor's effect deviates significantly from a flat line, although the precise shape can be more complex than a simple linear function. An Edf near 1 suggests a nearly linear relationship, whereas values exceeding 1 imply curvature. However, GAM summaries alone do not clarify how the effect changes across the predictor's range. Therefore, we used partial effect plots to illustrate whether relationships were monotonic, U-shaped, or exhibited thresholds. To further elucidate these patterns, we employed the *gratia* package to compute the first derivative of each smooth, highlighting statistically significant increases or decreases in the smooth function. A positive derivative signifies an increasing partial effect of the predictor, whereas a negative derivative reflects a decreasing effect. Where the derivative and its 95% confidence interval exclude zero, a statistically significant upward or downward trend is indicated. Points at which the derivative crosses zero typically mark local maxima or minima, providing insights into pivotal thresholds within the meteorology-disease association.

#### 2.4.4 Sensitivity analysis

To evaluate the robustness of our findings to alternative specifications of socioeconomic and healthcare access adjustment, we conducted two sets of sensitivity analyses. First, SDI was replaced by the age- and sex-specific population density of individuals aged 55 years and older as an alternative proxy for demographic structure and exposure distribution:

**Sensitivity Model S1:** DALYs ~ Population Density (55+) + s(Meteorological factors) + s(Year) + Obesity Prevalence + Smoking Rate

Second, to examine the influence of healthcare accessibility on the estimated meteorology-MSK associations, SDI was alternatively replaced by principal components derived from multiple BRFSS-based healthcare access indicators. The first two principal components was included as a composite healthcare accessibility index:

**Sensitivity Model S2:** DALYs ~ Healthaccess_ PC1 + Healthaccess_ PC2 + s(Meteorological factors) + s(Year) + Obesity Prevalence + Smoking Rate

Consistency of the estimated meteorology-MSK associations across the primary model (Model D) and the two sensitivity models (S1 and S2) was used to assess the robustness of our findings to alternative confounding control strategies.

Recognizing sex differences in MSK disease burdens, we repeated the entire analytical process (Model A–D and Sensitivity Model 1–2) for males and females. Model specifications remained the same except that behavioral factors, population density, and healthcare access were replaced with a sex-specific measure, allowing for comparability while identifying potential sex-based differences in meteorology-disease relationships.

## 3 Results

### 3.1 Burden (DALYs) of MSK diseases in 2021

**Figure 1:**
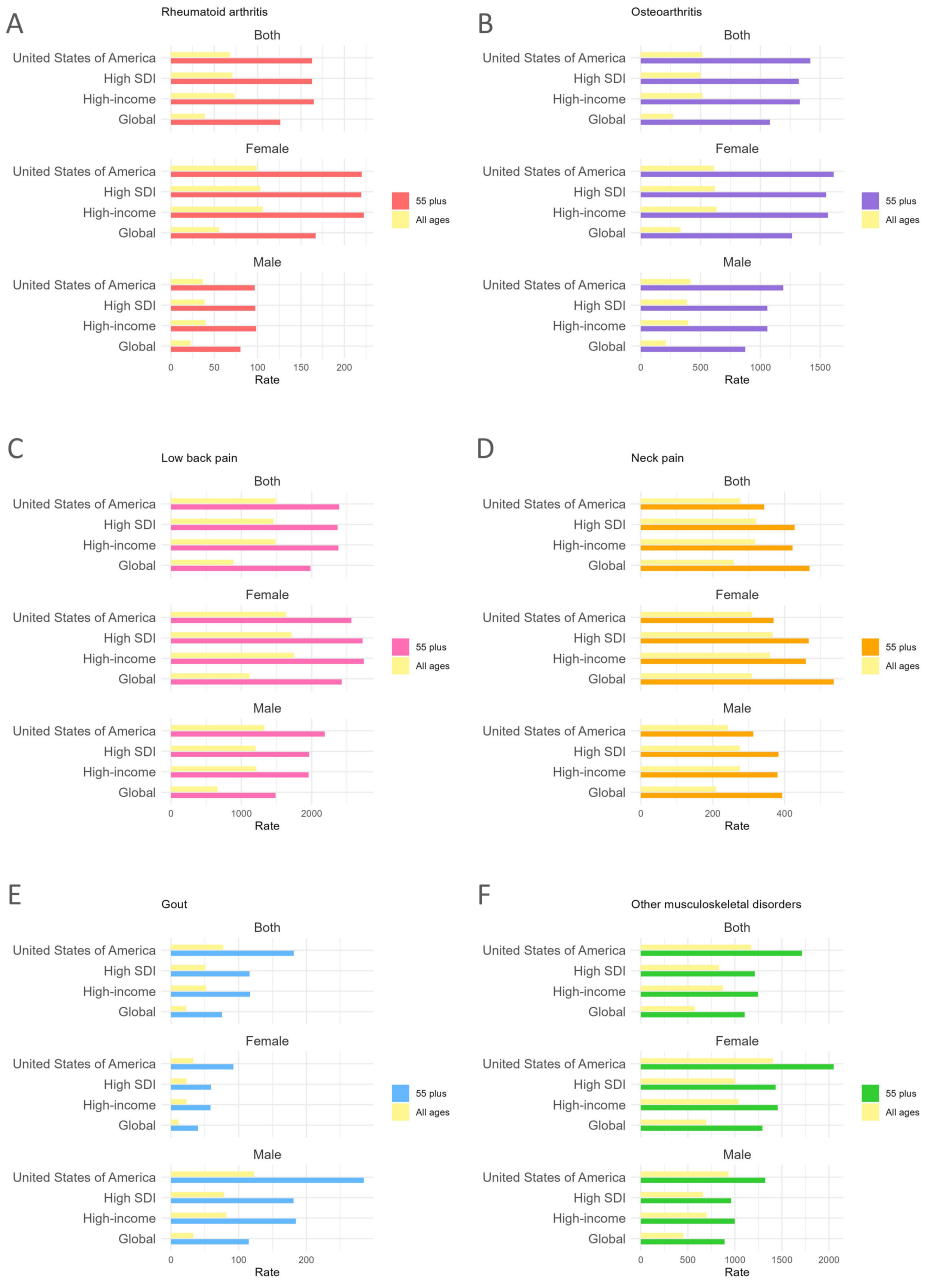
The national-level disease burden of six musculoskeletal disorders (2021). The disease burden was measured by the rates (years per 100,000 people) of DALYs (Disability-Adjusted Life-Years) attributed to six musculoskeletal subgroups. 55 plus: estimates for adults aged 55 and over; all ages: estimates for the whole population. **(A)** rheumatoid arthritis; **(B)** osteoarthritis; **(C)** low back pain; **(D)** neck pain; **(E)** gout; **(F)** other musculoskeletal disorders.

In 2021, MSK disorders accounted for 6.22 million DALYs (95% UI: 4.56 to 8.50 million) among U.S. adults aged 55 and older, with a rate of 6,205.82 per 100,000 people (95% UI: 4,553.48 to 8,477.80), significantly higher than that of the general population (3,593.56 per 100,000 people, 95% UI: 2,661.28 to 4,736.82). Women aged 55 and older experienced a higher MSK burden than age-matched men, with a total of 3.71 million DALYs (95% UI: 2.72 to 5.09 million) and a rate of 6,907.60 per 100,000 people (95% UI: 5,058.32 to 9,466.91). In comparison, men had 2.51 million DALYs (95% UI: 1.84 to 3.41 million) with a lower rate of 5,394.06 per 100,000 people (95% UI: 3,952.02 to 7,334.67). Among the six MSK subtypes, low back pain contributed the largest share of the total DALY burden (38.53%), followed by other musculoskeletal diseases (27.63%), osteoarthritis (22.75%), neck pain (5.53%), gout (2.93%), and rheumatoid arthritis (2.63%) (see Supplementary Figure 3). Consistently, low back pain posed the greatest burden across both sexes in the United States (Rate: 2,389.40 per 100,000 people, 95% UI: 1,710.77 to 3,107.34) (Figure 1C), whereas rheumatoid arthritis had the lowest burden (Rate: 162.80 per 100,000 people, 95% UI: 124.63 to 205.59) (Figure 1A).

Compared to high-SDI and high-income regions, the United States exhibited a more pronounced male burden of gout (Rate: 284.74 per 100,000 people, 95% UI: 198.30 to 404.69) and the greatest female disparity in other musculoskeletal diseases (Rate: 2,049.22 per 100,000 people, 95% UI: 1,463.55 to 2,734.11), as shown in Figures 1E, F. Notably, as Figure 1D indicates, neck pain was the only MSK subtype with a lower burden compared to high-SDI and high-income regions.

In the United States, age amplified MSK burdens across all conditions. According to Figure 1B, osteoarthritis demonstrated the largest age-related disparity, with a DALY rate of 1,416.48 per 100,000 people (95% UI: 700.38 to 2,872.48) among the old adults of both sexes, compared to 514.29 per 100,000 people (95% UI: 249.29 to 1,041.24) in the general population. The state-level burden of MSK diseases was available in Supplementary Figure 4.

### 3.2 Temporal change in burden (DALYs) of MSKs

Joinpoint analysis indicated significant improvements in the DALY rates of osteoarthritis, neck pain, gout, and other musculoskeletal disorders for both sexes (see Supplementary Table 6). Males generally showed larger annual percent changes (AAPC) than females in osteoarthritis (females: 0.772%, 95%CI: 0.704 to 0.841; males: 1.462%, 95% CI: 1.024 to 1.902) and other musculoskeletal disorders (females: 0.773%, 95%CI: 0.719 to 0.827; males: 1.339%, 95% CI: 1.283 to 1.396).

**Figure 2:**
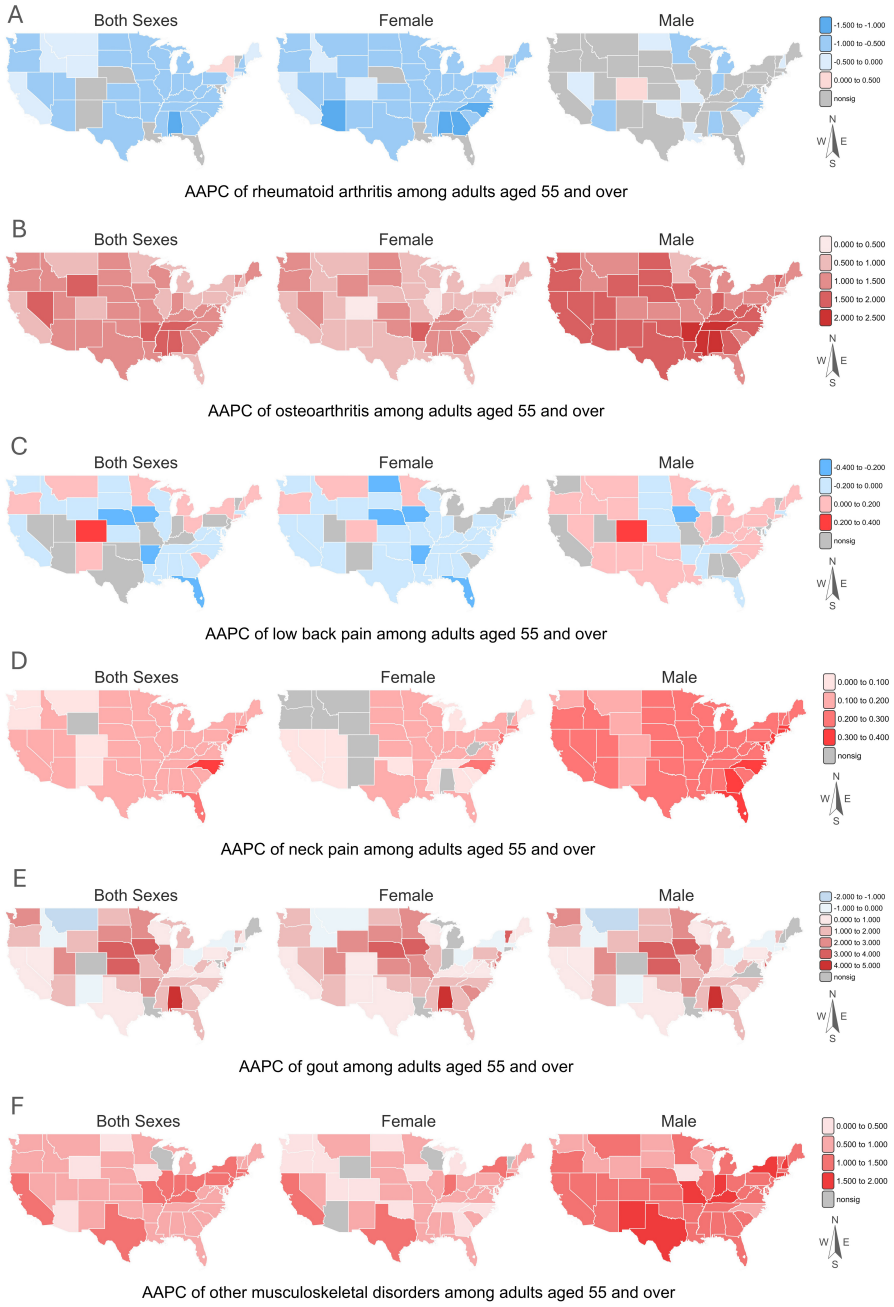
The state-level temporal trend of six musculoskeletal disorders among adults aged 55 and over (from 2013 to 2021; nosig: no statistical significance at 0.05 level). The temporal trend was identified by the Average Annual Percent Change (AAPC, %) using Joinpoint analysis. There were only 49 location units mapped as Alaska, Hawaii, Fed States of Micronesia, and Puerto Rico were excluded in the final modeling analysis. **(A)** rheumatoid arthritis; **(B)** osteoarthritis; **(C)** low back pain; **(D)** neck pain; **(E)** gout; **(F)** other musculoskeletal disorders.

As shown in Figure 2B, a positive trend was consistently observed with respect to osteoarthritis. For men, the AAPC ranged from 0.697% (95% CI: 0.684 to 0.709) in Massachusetts to 2.295% (95% CI: 1.341 to 3.259) in Alabama. For women, the AAPC ranged from 0.285% (95% CI: 0.152 to 0.418) in Delaware to 1.573% (95% CI: 1.151 to 1.996) in Arkansas. A narrowly greater increase in the overall burden of gout among women (females: 0.977%, 95% CI: 0.890 to 1.065; males: 0.772%, 95%CI: 0.657 to 0.886), though Alabama reported the steepest state-specific growth for both sexes (females: 4.006%, 95% CI: 2.585 to 5.446; males: 4.122 %, 95%CI: 2.593 to 5.673) (see Figure 2E). Regarding other musculoskeletal disorders, the highest levels were found in Texas (males: 1.826%, 95%CI: 1.724 to 1.929; females: 1.213%, 95%CI: 0.989 to 1.438) and New York (males: 1.818%, 95%CI: 1.791 to 1.845; females: 1.179%, 95%CI: 1.027 to 1.332), as shown in Figure 2F. Meanwhile, there was an overall downward trend in rheumatoid arthritis among the whole population, excepted for females in New York (0.217%, 95% CI: 0.101 to 0.332) and males in Colorado (0.265%, 95% CI: 0.174 to 0.357) (see Figure 2A). As for low back pain and neck pain, males experienced a larger scale of burden growth than females at the state-level, as Figures 2C, D illustrates.

### 3.3 The performance of model fit and parametric covariates

As shown in Table 1, transitioning from Model A (without meteorological covariates) to Model B and Model C (with meteorological covariates) typically led to reduced sigma^2^ and AIC values and improved R^2^, most notably in rheumatoid arthritis, osteoarthritis and gout. This improvement indicates that meteorological effects contribute explanatory value beyond SDI. Notably, the fit of rheumatoid arthritis was greatly improved when including linear meteorological terms. In contrast, allowing for non-linear meteorological effects (Model C) markedly enhanced model performance over linear meteorological terms (Model B) for osteoarthritis. For gout, model fit improved in a stepwise and progressive manner from Model A to Model B and then to Model C, with modest but consistent gains in explained variance at each step. The inclusion of obesity prevalence, smoking rate, and a smooth function for year for year in Models D resulted in slight improvements in model performance for osteoarthritis, low back pain and gout, suggesting behavioral factors and unexplained temporal dynamics contribute modestly to disease burden beyond the initial covariates.

**Table 1:** Model performance comparisons for both sexes.

**Rheumatoid arthritis**	***R^2^***	**Sigma^2^**	**ML**	**AIC**	**Osteoarthritis**	***R^2^***	**Sigma^2^**	**ML**	**AIC**
Model A	0.124	927.915	−2,131.413	4,268.826	Model A	0.076	6,914.729	−2,574.280	5,154.561
Model B	0.585	445.539	−1,966.608	3,951.215	Model B	0.139	6,534.056	−2,558.760	5,135.520
Model C	0.681	337.081	−1,895.310	3,857.460	Model C	0.552	3,347.192	−2,402.749	4,866.374
Model D	0.684	333.392	−1,891.608	3,855.284	Model D	0.572	3,198.933	−2,393.230	4,844.315
**Low back pain**	***R** ^2^*	**Sigma** ^2^	**ML**	**AIC**	**Neck pain**	***R** ^2^*	**Sigma** ^2^	**ML**	**AIC**
Model A	0.359	14,399.192	−2,736.021	5,478.042	Model A	0.046	49.326	−1,484.357	2,974.714
Model B	0.452	12,480.905	−2,701.461	5,420.923	Model B	0.097	47.307	−1,472.108	2,962.216
Model C	0.571	9,614.985	−2,638.361	5,324.371	Model C	0.320	35.089	−1,401.823	2,845.197
Model D	0.588	9,231.145	−2,628.774	5,306.756	Model D	0.325	34.813	−1,398.658	2,845.154
**Gout**	***R** ^2^*	**Sigma** ^2^	**ML**	**AIC**	**Other musculoskeletal disorders**	***R** ^2^*	**Sigma** ^2^	**ML**	**AIC**
Model A	0.053	641.865	−2,050.145	4,106.290	Model A	0.080	25,510.817	−2,862.132	5,730.264
Model B	0.259	508.912	−1,995.932	4,009.863	Model B	0.151	23,881.107	−2,844.541	5,707.082
Model C	0.483	349.709	−1,907.164	3,864.142	Model C	0.326	18,659.129	−2,785.255	5,615.004
Model D	0.516	327.447	−1,889.572	3,842.931	Model D	0.324	18,721.970	−2,785.022	5,618.517

**Model A:** DALYs ~ SDI.

**Model B:** DALYs ~ SDI + Meteorological factors.

**Model C:** DALYs ~ SDI + s (Meteorological factors).

**Model D:** DALYs ~ SDI + s (Meteorological factors) + s (Years) + Obesity Prevalence + Current Smoking Rate.

**R**^2^**:** Explained Deviance; higher values indicate better model performance.

**Sigma**^2^**:** Residual Variance; lower values reflect tighter fit to observed data.

**ML:** Maximum Likelihood; fewer negative values indicate improved fit.

**AIC:** Akaike Information Criterion; lower values indicate an optimal balance between model complexity and fit.

Similar patterns were observed in the sex-stratified analyses (see Supplementary Tables 7, 8), although notable sex-based differences emerged in baseline Model A (SDI only). Specifically, SDI predicted distinctly better for low back pain and gout among females. Additionally, the inclusion of meteorological variables (from Model A to Model B) resulted in a substantially greater improvement in model fit for rheumatoid arthritis among females, underscoring sex-specific differences in meteorology-disease association. Overall, Model D retained as the primary analytic model, as it jointly accounted for inter-annual meteorological variability, behavioral risk factors, and residual temporal trends, thereby providing the most comprehensive and biologically plausible representation of MSK burden patterns across outcomes and sexes. Although Model C yielded comparable performance for other musculoskeletal disorders, Model D offered superior covariate adjustment and interpretability across most outcomes.

As presented in Table 2A, the associations between SDI and the burden estimates (both sexes combined) remained consistently significant for rheumatoid arthritis, osteoarthritis, low back pain and neck pain when progressing from Model B (linear meteorological terms) to Model D (smooth terms for meteorological and temporal variables with covariate adjustment), though the significance shifted for osteoarthritis among males (Supplementary Tables 9A, 10A). The consistent statistical significance of SDI observed in sex-stratified models extended specifically to gout among females and other musculoskeletal disorders among males. Interestingly, the directions and magnitudes of its associations varied by musculoskeletal subtype, indicating substantial effect heterogeneity rather than a uniform protective or adverse influence of socioeconomic development.

**Table 2:** Model estimates comparisons for both sexes (A: Parametric Coefficients; B: Smooth terms; ^*^p < 0.05, ^**^p < 0.01, ^***^p < 0.001).

**A: Parametric coefficients**									
**Rheumatoid arthritis**	**Model B**		**Model D**		**Osteoarthritis**	**Model B**		**Model D**	
	**Estimate**	**Std. Error**	**Estimate**	**Std. Error**		**Estimate**	**Std. Error**	**Estimate**	**Std. Error**
Intercept	1,955.171	312.355	176.904 ^***^	0.930	Intercept	−3,267.656 ^**^	1,196.182	1,350.526 ^***^	2.865
SDI	−15.912^***^	1.109	−19.451^***^	2.287	SDI	20.935 ^***^	4.248	35.016 ^***^	6.814
Obesity prevalence	–	–	−6.228 ^**^	2.169	Obesity prevalence	–	–	30.544 ^***^	6.425
Smoking rate	–	–	0.949	1.887	Smoking rate	–	–	19.485 ^**^	5.642
**Low back pain**	**Model B**		**Model D**		**Neck pain**	**Model B**		**Model D**	
	**Estimate**	**Std. Error**	**Estimate**	**Std. Error**		**Estimate**	**Std. Error**	**Estimate**	**Std. Error**
Intercept	7,829.959 ^***^	1,653.214	2,396.730 ^***^	4.708	Intercept	344.249 ^**^	101.781	345.200 ^***^	0.299
SDI	−92.780 ^***^	5.872	−55.813 ^***^	10.177	SDI	1.502 ^***^	0.361	2.534 ^***^	0.632
Obesity prevalence	–	–	43.270 ^***^	7.189	Obesity prevalence	–	–	1.304 ^*^	0.643
Smoking rate	–	–	16.127	9.432	Smoking rate	–	–	−0.112	0.558
**Gout**	**Model B**		**Model D**		**Other musculoskeletal disorders**	**Model B**		**Model D**	
	**Estimate**	**Std. Error**	**Estimate**	**Std. Error**		**Estimate**	**Std. Error**	**Estimate**	**Std. Error**
Intercept	270.929	333.831	176.202 ^***^	0.916	Intercept	4,353.753	2,286.827	1,637.092 ^***^	6.887
SDI	−3.069 ^*^	1.186	0.006	2.304	SDI	37.739 ^***^	8.122	27.361	16.276
Obesity prevalence	-	-	−3.945	2.078	Obesity prevalence	–	–	7.088	14.595
Smoking rate	-	-	8.299 ^***^	1.824	Smoking rate	–	–	−7.682	13.395
**B: Smooth terms**									
**Rheumatoid arthritis**	**Model B**		**Model D**		**Osteoarthritis**	**Model B**		**Model D**	
	**Estimate**	**Std. Error**	**Estimate**	**Std. Error**		**Estimate**	**Std. Error**	**Estimate**	**Std. Error**
TRAN_ANN	7.567^***^	1.558	4.781^***^	9.000	TRAN_ANN	−18.662^**^	5.966	5.617^***^	9.000
MEAN_TAVG	−4.279^**^	1.374	2.344^**^	9.000	MEAN_TAVG	−11.468^*^	5.264	3.700^***^	9.000
RRAV_ANN	−1.345	1.542	4.732^***^	9.000	RRAV_ANN	1.851	5.905	0.000	9.000
MEAN_RRAV	−5.544	9.829	2.650^***^	9.000	MEAN_RRAV	−46.699	37.642	0.904^**^	9.000
MEAN_ASTP	−225.571 ^***^	37.436	3.935^***^	9.000	MEAN_ASTP	527.605^***^	143.365	3.699^***^	9.000
MEAN_AWND	48.079^***^	8.607	4.991^***^	9.000	MEAN_AWND	88.696^**^	32.960	3.370^***^	9.000
Year	–	–	1.753^**^	4.000	Year	–	–	2.226^***^	4.000
**Low back pain**	**Model B**		**Model D**		**Neck pain**	**Model B**		**Model D**	
	**Estimate**	**Std. Error**	**Edf**	**Ref.df**		**Estimate**	**Std. Error**	**Edf**	**Ref.df**
TRAN_ANN	−14.870	8.246	6.246^***^	9.000	TRAN_ANN	1.344^**^	0.508	1.500^*^	9.000
MEAN_TAVG	−8.920	7.275	0.873	9.000	MEAN_TAVG	−0.345	0.448	3.148^**^	9.000
RRAV_ANN	−12.144	8.161	0.006	9.000	RRAV_ANN	1.194^*^	0.502	0.361	9.000
MEAN_RRAV	−180.544	52.024	2.751^***^	9.000	MEAN_RRAV	−0.842	3.203	1.441^**^	9.000
MEAN_ASTP	−599.282^**^	198.141	3.855^***^	9.000	MEAN_ASTP	4.364	12.199	0.000	9.000
MEAN_AWND	120.117 ^**^	45.554	2.236^**^	9.000	MEAN_AWND	−9.383^**^	2.805	5.270^***^	9.000
Year	–	–	0.000	4.000	Year	–	–	3.576^***^	4.000
**Gout**	**Model B**		**Model D**		**Other musculoskeletal disorders**	**Model B**		**Model D**	
	**Estimate**	**Std. Error**	**Edf**	**Ref.df**		**Estimate**	**Std. Error**	**Edf**	**Ref.df**
TRAN_ANN	−12.842^***^	1.665	3.642^***^	9.000	TRAN_ANN	−0.481	11.406	0.003	9.000
MEAN_TAVG	−2.810	1.469	2.462^**^	9.000	MEAN_TAVG	−26.027^*^	10.063	1.578^***^	9.000
RRAV_ANN	5.250^**^	1.648	2.518^***^	9.000	RRAV_ANN	−5.637	11.289	0.543	9.000
MEAN_RRAV	9.554	10.505	2.662^**^	9.000	MEAN_RRAV	−215.516^**^	71.962	2.319^*^	9.000
MEAN_ASTP	−9.282	40.010	3.713^***^	9.000	MEAN_ASTP	−221.187	274.081	4.834^***^	9.000
MEAN_AWND	−15.444	9.199	4.040^***^	9.000	MEAN_AWND	18.012	63.013	4.332^***^	9.000
Year	–	–	2.527^***^	4.000	Year	–	–	1.730	4.000

**Model B**: DALYs ~ SDI + Population Density + Meteorological factors.

**Model D**: DALYs ~ SDI + Population Density + s (Meteorological factors) + s (Years).

**Estimate**: Linear coefficient.

**Std. Error**: Standard Error.

**Edf**: Estimated Degrees of Freedom.

**Ref.df**: Reference Degrees of Freedom.

Behavioral factors (obesity prevalence and smoking rate) exhibited consistent positive associations with osteoarthritis across sexes in Model D, with obesity prevalence remaining statistically significant in both Sensitivity Models S1 and S2 (see Supplementary Tables 11A, 12A, 13A). In contrast, more pronounced sex heterogeneity was observed in the associations between behavioral factors and rheumatoid arthritis, low back pain, gout, and other musculoskeletal disorders. Specifically, smoking rate showed persistent positive effects on the burden of low back pain among females and gout among males across both the main and sensitivity analyses.

### 3.4 The potential associations of inter-annual meteorological variability with burden of MSKs

As shown in Table 2B and Supplementary Tables 9B, 10B, for each MSK subtype, multiple meteorological variables were already statistically significant in the linear models (Model B). As the model specification progressed to non-linear smooth terms and additionally adjusted for behavioral covariates and temporal trends (Model D), the number of significant meteorological terms generally increased, indicating enhanced detection of non-linear exposure-burden relationships. Importantly, the majority of these significant associations remained robust in Sensitivity Models S1 and S2 (see Supplementary Tables 11B, 12B, 13B), supporting the stability of the observed effects.

#### 3.4.1 Annual average daily temperature

**Figure 3:**
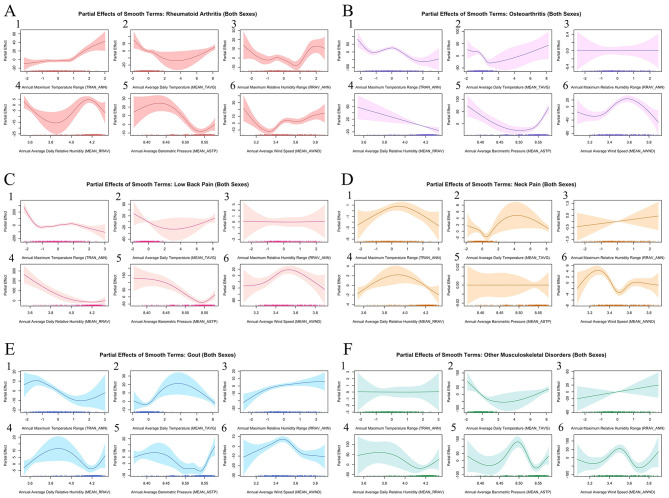
Partial effects of GAM smooth terms for both sexes (Model D). **(A)** rheumatoid arthritis; **(B)** osteoarthritis; **(C)** low back pain; **(D)** neck pain; **(E)** gout; **(F)** other musculoskeletal disorders. 1: TRAN_ANN, Annual maximum temperature range; 2: MEAN_TAVG, Annual average daily temperature; 3: RRAV_ANN: Annual relative humidity range; 4: MEAN_RRAV, Annual average daily relative humidity; 5: MEAN_ASTP, Annual average barometric pressure; 6: MEAN_AWND, Annual average wind speed.

GAM results indicated distinct non-linear associations between MEAN_TAVG (annual average daily temperature) and the burdens of rheumatoid arthritis, osteoarthritis, neck pain, gout, and other musculoskeletal disorders in both sexes (see Table 2B and Figures 3A_2_, B_2_, D_2_–F_2_). Specifically, these MSK conditions exhibited broadly similar patterns, with burdens initially declining at the lower exposure distribution of temperatures before rebounding at higher extremes. Further indicated by the first-derivative analysis (see Supplementary Figure 15), the direction of the temperature effects shifted within moderate exposure ranges between the standardized zero point and approximately one standard deviation units for osteoarthritis, neck pain and gout. According to sex-stratified analyses (see Supplementary Tables 9B, 10B, and Supplementary Figures 6, 7), MEAN_TAVG displayed the fall-and-rise pattern in the burden of low back pain and gout exclusively for males. Similarly, the association between temperature exposures and the burden of other musculoskeletal disorders was more pronounced among males compared to females. For low back pain, the smooth term for MEAN_TAVG was not statistically significant, suggesting no clear association after adjustment (Figure 3C_2_; Table 2B).

#### 3.4.2 Annual maximum temperature range

Despite fluctuations, the relationships between TRAN_ANN (annual maximum temperature range) and disease burden revealed distinct yet related patterns among rheumatoid arthritis, osteoarthritis, low back pain, and gout (see Table 2B and Figures 3A_1_–C_1_, E_1_ and Supplementary Figure 14), with consistent thresholds identified at around one standard deviation of the mean for both sexes. Below at approximately one standard deviation below the mean of the exposure distribution, osteoarthritis and low back pain burdens declined steeply. Across the mid-range of the standardized distribution (−1 to +1 SD), these conditions transitioned into a plateau or a small rise, but shifted directions as approaching the higher extremes. In contrast, the associations were comparatively weaker and closer to flat for neck pain and other musculoskeletal disorders (Figures 3D_1_ and 3F_1_; Table 2B). Sex-stratified analyses (see Supplementary Figures 6, 7) shows that the negative association of TRAN_ANN and gout burden were more pronounced among males, whereas rheumatoid arthritis exhibited the opposite sex-specific pattern at the higher tail of the standardized distribution of inter-annual temperature variability, with stronger effects among females.

#### 3.4.3 Annual average daily relative humidity

For MEAN_RRAV (annual average daily relative humidity), the smooth terms were statistically significant across all MSK subtypes and sexes (Figures 3A_4_–F_4_; Table 2B). Specifically, low back pain showed a sharp decline that leveled off within the most frequently sampled range around the median (4.20 log-scaled units), as Figure 3C_4_ and Supplementary Figure 17 indicates. In contrast, rheumatoid arthritis, gout, and other musculoskeletal disorders exhibited more noticeable fluctuations: rheumatoid arthritis followed an inverted U-shaped pattern across the empirically observed exposure range, with a minimum near the lower 5th percentile (3.84 log-scaled units) and a peak near the median (4.20 log-scaled units), while gout and other musculoskeletal disorders displayed the opposite curvature (see Figures 3A_4_, E_4_, F_4_ and Supplementary Figure 17). Notably, the magnitude and variability of the MEAN_RRAV effects on gout and other musculoskeletal disorders were more noticeable among males than females (see Supplementary Figures 6, 7).

#### 3.4.4 Annual maximum relative humidity range

Regarding RRAV_ANN (annual maximum relative humidity range), gout revealed a predominantly positive relationship, whereas rheumatoid arthritis exhibited a roughly U-shaped trajectory featuring an inflection around one standard deviation above the mean (see Figures 3A_3_, E_3_ and Supplementary Figure 16). Additionally, sex-specific analyses indicated a stronger impact of RRAV_ANN on gout burden in males compared to females, as presented in Supplementary Figures 6, 7. By comparison, the associations between RRAV_ANN and osteoarthritis, low back pain, neck pain, and other musculoskeletal disorders were not statistically significant in the main model (Figures 3B_3_–D_3_, F_3_; Table 2B).

#### 3.4.5 Annual average barometric pressure

In Figures 3A_5_–C_5_, E_5_, F_5_, the curve of MEAN_ASTP (annual average barometric pressure) showed a common breakpoint within the interquartile range (between 8.50 and 8.55 log-scaled units) across rheumatoid arthritis, osteoarthritis, low back pain, gout and other musculoskeletal disorders, as confirmed by derivative analyses (see Supplementary Figure 18), although the burden of other musculoskeletal disorders exhibited greater variability. For male-specific burden of gout, MEAN_ASTP demonstrated a more pronounced threshold-like effect within the central range of the exposure distribution, compared that of females (see Supplementary Figures 6, 7). No clear association was observed for neck pain (Figure 3D_5_; Table 2B).

#### 3.4.6 Annual average wind speed

Overall, MEAN_AWND (annual average wind speed) displayed great non-linear complexity across all MSK conditions, with a consistent turning point near the median of the exposure distribution (at approximately 3.50 log-scaled units), where rheumatoid arthritis, osteoarthritis, low back pain, gout, and other musculoskeletal disorders reached their maximum burdens (see Figures 3A_6_–C_6_, E_6_, F_6_ and Supplementary Figure 19). In contrast, neck pain uniquely attained its minimum burden at this point, although the overall effect size was relatively small (see Figure 3D_6_). Moreover, the MEAN_AWND-gout association exhibited greater magnitude and variability across the empirically observed exposure range among males than females (Supplementary Figures 6, 7).

## 4 Discussion

In 2021, older adults in the United States experienced considerably higher burdens from six musculoskeletal disorders compared to global averages, with gout among males and other musculoskeletal diseases among females showing the most substantial disparities. Over the study period, the burden for most MSK disorders increased, except rheumatoid arthritis and low back pain, which exhibited a declining trend. Correlation analyses revealed significant bivariate associations between specific meteorological variables and disease burdens. Subsequent modeling using GAMs, allowing for non-linear meteorology-health relationships, jointly accounted for behavioral risk factors and residual temporal trends, significantly improved model performance relative to baseline models, uncovering nuanced and disorder-specific effects of ambient meteorological conditions with potential common thresholds across MSK conditions. Notably, beyond the impact of annual average conditions, inter-annual fluctuations in temperature and humidity consistently exhibited significant associations with MSK burdens. Furthermore, absolute levels of wind speed and barometric pressure was significantly correlated with nearly all disorders studied, underscoring their broad population-level relevance. Sex-based differences in meteorology-disease associations were also evident, particularly for gout and rheumatoid arthritis. Rather than implying direct causality, our findings primarily aim to advance understanding of population-level meteorology-MSK associations and to inform context-sensitive public health planning and preventive strategies in aging societies.

Previous research has long suggested an association between meteorological factors and MSK conditions (20, 36, 37), yet findings remain inconclusive. Notably, gout is supported by the strongest evidence implicating weather conditions among the MSKs examined (17). Many studies report seasonal peaks in gout attacks driven by changes in air temperature, barometric pressure, humidity, and wind speed (12, 16, 21, 38). Several biological mechanisms have been proposed to explain these associations, including disrupted physiological homeostasis, enhanced renal reabsorption of urate, and dysregulation of innate immune responses (12, 21). Additionally, a meta-analysis of case-crossover studies indicated that the combination of high temperature and low humidity significantly increased the risk of gout flares (17). Consistent with this, our study found that gout burden reached a lowest level within moderate ranges of the annual average temperatures (MEAN_TAVG) but rose markedly at the higher end (most pronounced among males). Apart from the absolute temperatures, there is also evidence highlighting that greater temperature variability may induce acute gout episodes (12, 17). In contrast, our study revealed a moderate and negative association between annual maximum temperature range (TRAN_ANN) and gout burden, and this effect was again more pronounced among males. In particular, this inverse association was estimated after adjustment for mean annual temperature, suggesting that the effect of intra-annual temperature variability may be independent of average thermal exposure.

Similar regulatory effects of temperature have been previously explored for other MSK subtypes. For example, a retrospective longitudinal analysis from Morocco reported that increased daily minimum temperatures during summer were associated with reduced pain intensity in patients with rheumatoid arthritis (14). Similarly, a 14-year primary care study conducted in Spain observed fewer referrals for shoulder or arm pain in years when annual minimum temperatures were higher (39). In Australia, a web-based longitudinal study found associations between sudden temperature fluctuations and increased hip osteoarthritis pain (40). Moreover, nationwide big-data research from South Korea reported a stronger association between monthly temperature differences and the temporomandibular disorder prevalence compared to absolute temperatures alone (41).

In our current study, although we did not observe a consistent pattern of temperature effects across the six MSK subtypes, some common “breakpoints” were identified across six MSK subtypes in terms of annual average temperatures (MEAN_TAVG) or maximum temperature range (TRAN_ANN). These threshold points corresponded closely to local minima or maxima, indicating critical changes in the temperature-burden relationships. It is biologically plausible that extreme environmental conditions trigger adaptive physiological responses, thereby influencing the pathology of MSK disorders. For example, prolonged exposure to high temperatures can induce heat stress, altering immune function through changes in cytokine production and inflammatory signaling pathways (42). By contrast, cold exposure might exacerbate joint stiffness and pain in inflammatory arthritis, likely through transient reductions in peripheral blood flow and neuromuscular function (43, 44).

More importantly, the outcome of interest in this study was disease burden measured by DALYs, which reflects the chronic accumulation of disability and premature mortality rather than acute disease incidence or short-term symptom fluctuations. As such, the observed associations with the inter-annual meteorological variability are unlikely to represent direct biological triggers of acute pain episodes. Instead, long-term weather patterns, particularly those characterizing regions with greater seasonal temperature contrast, may serve as proxies for broader contextual influences, including habitual physical activity, occupational exposure, healthcare access, and meteorology-related adaptive behaviors, all of which can shape cumulative MSK burdens over time. Consistent with this interpretation, elevated summer temperatures can contribute to increased consumption of beer, sugary beverages, and high-purine foods, thereby raising uric acid levels and exacerbating gout risk (21). In parallel, extended periods of extreme heat or cold may discourage regular outdoor physical activity (22), while reduced physical activity levels can lead to weight gain, loss of muscle strength, diminished joint support, and ultimately increased burden of conditions such as osteoarthritis or low back pain (23). Recent evidence further suggests that meteorological conditions may influence musculoskeletal health primarily by shaping physical activity patterns, activity constraints, and the utilization of rehabilitation and physical therapy resources, rather than through isolated direct biomechanical or inflammatory effects alone (24–26). In addition, substantial individual variability, shaped by genetic predisposition, lifestyle factors, psychological resilience, pain perception, coping mechanisms, or baseline disease severity, can further explain heterogeneous vulnerability to meteorological exposures (45–48).

Humidity has previously been implicated in gout flare-ups, with Neogi et al. reporting a reverse J-shaped association between average relative humidity and recurrent gout attacks, characterized by increased risk at higher humidity extremes (16). Our analyses revealed predictive potential of both humidity-related metrics (annual average and range) for gout and rheumatoid arthritis. Given previous research primarily emphasized short-term humidity exposure, our findings highlight the inter-annual humidity variability may also influence MSK burden through cumulative, indirect pathways, offering new avenues for future research.

Compared to temperature, humidity, wind speed, and barometric pressure are less consistently documented in relation to MSK aggravation. Nonetheless, some research has suggested a mitigating role of lower barometric pressure and stronger winds in chronic MSK pain (14, 49, 50). Under GAM specifications in our study, the population-level associations of most MSK disorders and annual average barometric pressure (MEAN_ASTP) demonstrated a similar U-shaped pattern. Conversely, annual average wind speed (MEAN_AWND) exhibited more distinct, disease-specific trends despite sharing common turning points. In this context, unlike temperature and humidity, which were characterized using both annual mean and annual range metrics to better capture physiologically relevant exposure patterns, MEAN_ASTP and MEAN_AWND were represented solely by annual mean values. Thus, their associations with MSK burden are more plausibly interpreted as population-level statistical correlations that likely arise from complex environmental and structural pathways.

Despite meaningful insights emerging from various study designs so far (51, 52), systematic reviews and bigdata-based epidemiological studies have often questioned the assumption that meteorological factors directly trigger MSK pain onset or exacerbation (17, 36, 45, 46). These conflicting findings possibly stem from methodological limitations commonly encountered in environmental-health research, including small sample sizes, short follow-up periods, misclassification of meteorological exposure, heterogeneity in outcome measurements, and insufficient adjustment for potential confounding factors (17, 20). The case-crossover design is frequently employed to assess the effects of transient environmental exposures on the risk of acute MSK pain events whose validity depends on appropriate selection of control time windows (pain-free periods). Besides, due to the self-reported nature of symptom exacerbations, participants may recall or report environmental conditions differently during episodes of pain compared to pain-free intervals, introducing recall bias. Another substantial concern is the inconsistent treatment of environmental variables across studies, complicating comparisons and synthesis of results. For instance, some studies utilize daily averages or maximum values, whereas others incorporate more precise hourly or instantaneous records. Furthermore, relatively few studies have adequately accounted for lagged effects (delayed weather impact on pain) or lead effects (pain preceding weather changes), potentially overlooking long-term or cumulative meteorological impacts on MSK symptoms (20). Addressing these methodological limitations through standardized exposure assessments, clearly defined referent periods, rigorous design choices, and long-term prospective studies will be critical to advancing the understanding of meteorological effects on MSK health outcomes.

Globally, MSK conditions rank prominently among the top contributors to the loss of healthy years, with burdens that accumulate with advancing age, and disproportionately affect older adults. The rapidly aging global population amplifies their importance as a public health priority. Our study identified distinct, occasionally contrasting patterns of meteorology-disease associations across the six included MSK conditions. Notably, we also observed sex-based disparities, particularly evident in gout and rheumatoid arthritis. The use of GAMs and derivative analyses revealed that both the direction and magnitude of meteorological effects varied across certain thresholds, characterized by some common breakpoints located around the central portion of the exposure distribution (i.e., near the mean or median on the standardized scale). These breakpoints likely represent transition zones in relative exposure intensity experienced by a large proportion of the population, where modest shifts may interact with behavioral adaptation, physiological tolerance, and healthcare-system buffering capacity, thereby exerting a disproportionate influence on the long-term accumulation of MSK disease burden at the regional level. Based on these insights, observed non-linear and sex-specific patterns are more plausibly interpreted as reflecting the integrated influence of environmental conditions, behavioral adaptations, psychosocial vulnerability, and healthcare utilization over prolonged periods, rather than direct short-term physiological triggering alone. To move beyond ecologic inference, future research should prioritize longitudinal and individual-level study designs that integrate repeated symptom assessment with detailed psychosocial, behavioral, and social-contextual data. Such approaches will be essential for clarifying how meteorological variability interacts with aging, lifestyle, healthcare access, and broader structural determinants of health in shaping the long-term population burden of MSK disorders. Ultimately, this integrated perspective may inform context-sensitive prevention strategies and resource planning for MSK disorders in an era of demographic aging and increasing environmental variability.

Several limitations should be noted when interpreting our findings. First, because we conducted a secondary analysis of GBD study data, the results may be influenced by biases inherent in GBD modeling, and our scope may exclude certain MSK conditions not captured by that study. Second, meteorological elements are usually highly correlated, and multicollinearity challenges the robustness of studies on environment-health associations. In our design, we improved numerical stability by rescaling predictor variables and mitigated multicollinearity by employing GAMs with penalized cubic regression splines, using restricted maximum likelihood and automatic smoothness selection. Additionally, VIFs were assessed to confirm minimal residual collinearity (VIF < 5) among predictors.

Third, meteorological exposures were generated by IDW over the continental domain and then averaged within state polygons, whose precision depends on station density. Coverage is limited for extended elements, and some state estimates necessarily borrow information from neighboring states. As a result, values are spatially smoothed and more uncertain in sparsely monitored areas, although IDW still recovers broad, physically plausible continental gradients. Fourth, this study is based on state-level aggregation of both meteorological exposures and MSK burden outcomes, which inevitably introduces exposure misclassification, particularly in meteorologically heterogeneous states. This aggregation also implies an inherent risk of ecological fallacy, whereby population-averaged associations cannot be directly translated into individual-level exposure–outcome relationships. This form of misclassification is expected to be largely non-differential with respect to DALYs and would therefore predominantly bias associations toward the null rather than generate systematic false-positive findings. To partially address this limitation, we conducted multiple sensitivity analyses incorporating alternative demographic and healthcare-access adjustments as well as sex-specific models, and the overall patterns of the main associations remained generally consistent. Fifth, although we adjusted for key sociodemographic, behavioral, and healthcare-access factors across the main and sensitivity model specifications, residual confounding by unmeasured individual-level factors such as comorbidities, detailed lifestyle behaviors, and individual healthcare utilization patterns may still confound or modify the observed meteorological-MSK relationship. Sixth, our analysis is explicitly burden-oriented and population-based, so there is a potential for ecological fallacy. The annual meteorological summaries used in this study capture regional environmental contexts under which MSK burden accumulates over time, rather than individual-level acute physiological exposures. Accordingly, the observed associations should be not interpreted as evidence of short-term weather-triggered symptom dynamics. Finally, our findings are specific to individuals aged 55 and older in the United States, and the results may therefore not generalize to younger populations or low- or middle-income regions with markedly different meteorological contexts, healthcare systems, or lifestyle factors.

## 5 Conclusion

By integrating data from the Global Historical Climatology Network-Daily (GHCN-D), the Behavioral Risk Factor Surveillance System (BRFSS) and Global Burden of Disease (GBD) 2021, this study adopted an ecological design to explore condition-specific associations between inter-annual meteorological variability and musculoskeletal (MSK) disorders in older adults in the United States. Apart from the yearly mean summaries, annual temperature and humidity variations were consistently associated with MSK burdens at the population level. Additionally, annual average wind speed and barometric pressure were also associated with disease burdens across most disorders. Notably, sex-based differences were shown in meteorology-disease relationships, especially for gout and rheumatoid. Furthermore, General Additive Model (GAM) analyses highlighted common non-linear breakpoints across several MSK conditions. Given that Disability-Adjusted Life Years (DALYs) capture the cumulative impact of disability and premature mortality rather than acute symptom triggers, the observed associations should be interpreted as context-sensitive correlates of chronic disease burden operating through complex behavioral, psychosocial, and structural pathways. Collectively, our findings offer population-level support for the associations between inter-annual meteorological variability and MSK disorder burdens, reinforcing the importance of incorporating environmental variability into population-level MSK surveillance and burden forecasting, particularly in aging societies. Future research should prioritize longitudinal, individual-level studies that incorporate physiological, psychological, and behavioral data and social-contextual data. Such work will be critical for disentangling potential biological mechanisms from perceptual biases, behavioral and structural influences, and for clarifying how social resources, cultural beliefs, and healthcare access interact with meteorological variability in shaping long-term MSK disease burden. This integrated perspective is essential for informing population-based prevention and health-system planning for aging populations under ongoing environmental change.

## Data Availability

The original contributions presented in the study are included in the article/Supplementary material, further inquiries can be directed to the corresponding author.

## References

[1] Gill TK Mittinty MM March LM Steinmetz JD Culbreth GT Cross M . Global, regional, and national burden of other musculoskeletal disorders, 1990and#x2013;2020, and projections to 2050: a systematic analysis of the Global Burden of Disease Study 2021. Lancet Rheumatol. (2023) 5:e670–82. doi: 10.1016/S2665-9913(23)00232-1 37927903 PMC10620749

[2] Briggs AM Woolf AD Dreinhöfer K Homb N Hoy DG Kopansky-Giles D . Reducing the global burden of musculoskeletal conditions. Bull World Health Organ. (2018) 96:366–8. doi: 10.2471/BLT.17.204891 29875522 PMC5985424

[3] Ingram M Symmons DPM. The burden of musculoskeletal conditions. Medicine. (2018) 46:152–5. doi: 10.1016/j.mpmed.2017.12.005

[4] Liu S Wang B Fan S Wang Y Zhan Y Ye D. Global burden of musculoskeletal disorders and attributable factors in 204 countries and territories: a secondary analysis of the Global Burden of Disease 2019 study. BMJ Open. (2022) 12:e062183. doi: 10.1136/bmjopen-2022-062183 35768100 PMC9244680

[5] Institute for Health Metrics and Evaluation. Musculoskeletal disorders – Methods appendices. (2024). Available online at: https://www.healthdata.org/gbd/methods-appendices-2021/musculoskeletal-disorders (Accessed March 20, 2025).

[6] Briggs AM Cross MJ Hoy DG Sànchez-Riera L Blyth FM Woolf AD . Musculoskeletal health conditions represent a global threat to healthy aging: a report for the 2015 world health organization world report on ageing and health. Gerontologist. (2016) 56:S243–55. doi: 10.1093/geront/gnw002 26994264

[7] World Health Organization. Musculoskeletal conditions. (2024). Available online at: https://www.who.int/news-room/fact-sheets/detail/musculoskeletal-conditions (Accessed March 20, 2025).

[8] Sebbag E Felten R Sagez F Sibilia J Devilliers H Arnaud L. The world-wide burden of musculoskeletal diseases: a systematic analysis of the World Health Organization Burden of Diseases Database. Ann Rheum Dis. (2019) 78:844–8. doi: 10.1136/annrheumdis-2019-215142 30987966

[9] Bouziri H Roquelaure Y Descatha A Dab W Jean K. Temporal and spatial distribution of musculoskeletal disorders from 1990 to 2019: a systematic analysis of the global burden of disease. BMJ Public Health. (2023) 1:e000353. doi: 10.1136/bmjph-2023-000353 41127806 PMC12538050

[10] Patwardhan V Gil GF Arrieta A Cagney J Degraw E Herbert ME . Differences across the lifespan between females and males in the top 20 causes of disease burden globally: a systematic analysis of the Global Burden of Disease Study 2021. Lancet Public Health. (2024) 9:e282–94. doi: 10.1016/S2468-2667(24)00053-7 38702093 PMC11080072

[11] Cross M Ong KL Culbreth GT Steinmetz JD Cousin E Lenox H . Global, regional, and national burden of gout, 1990and#x2013;2020, and projections to 2050: a systematic analysis of the Global Burden of Disease Study 2021. Lancet Rheumatol. (2024) 6:e507–17. doi: 10.1016/S2665-9913(24)00117-6 38996590 PMC11263476

[12] Wu ZD Yang XK He YS Ni J Wang J Yin KJ . Environmental factors and risk of gout. Environ Res. (2022) 212:113377. doi: 10.1016/j.envres.2022.113377 35500858

[13] Gherscovici ED Mayer JM. Relationship of healthy building determinants with musculoskeletal disorders of the extremities: a systematic review. Cureus. (2023) 15:e37456. doi: 10.7759/cureus.37456 37091490 PMC10115431

[14] Azzouzi H Ichchou L. Seasonal and weather effects on rheumatoid arthritis: myth or reality? Pain Res Manag. (2020) 2020:5763080. doi: 10.1155/2020/5763080 32963656 PMC7492902

[15] Steffens D Maher CG Li Q Ferreira ML Pereira LS Koes BW . Effect of weather on back pain: results from a case-crossover study. Arthritis Care Res. (2014) 66:1867–72. doi: 10.1002/acr.22378 25044376

[16] Neogi T Chen C Niu J Chaisson C Hunter DJ Choi H . Relation of temperature and humidity to the risk of recurrent gout attacks. Am J Epidemiol. (2014) 180:372–7. doi: 10.1093/aje/kwu147 24993733 PMC4184385

[17] Ferreira ML Hunter DJ Fu A Raihana S Urquhart D Ferreira PH. Come rain or shine: is weather a risk factor for musculoskeletal pain? A systematic review with meta-analysis of case-crossover studies. Semin Arthritis Rheum. (2024) 65:152392. doi: 10.1016/j.semarthrit.2024.152392 38340613

[18] McAlindon T Formica M Schmid CH Fletcher J. Changes in barometric pressure and ambient temperature influence osteoarthritis pain. Am J Med. (2007) 120:429–34. doi: 10.1016/j.amjmed.2006.07.036 17466654

[19] McDougall JJ. Arthritis and pain. Neurogenic origin of joint pain. Arthritis Res Ther. (2006) 8:220. doi: 10.1186/ar2069 17118212 PMC1794504

[20] Beukenhorst AL Schultz DM Mcbeth J Sergeant JC Dixon WG. Are weather conditions associated with chronic musculoskeletal pain? Review of results and methodologies. Pain. (2020) 161:668–83. doi: 10.1097/j.pain.0000000000001776 32195783

[21] Zheng Y Jin M Cheng L Chen S Shen B Li J . Universal thermal climate index influences on uric acid levels and hyperuricemia: insights from a large-scale population study. Int Immunopharmacol. (2025) 149:114224. doi: 10.1016/j.intimp.2025.114224 39919459

[22] Ho JY Lam HYC Huang Z Liu S Goggins WB Mo PKH . Factors affecting outdoor physical activity in extreme temperatures in a sub-tropical Chinese urban population: an exploratory telephone survey. BMC Public Health. (2023) 23:101. doi: 10.1186/s12889-022-14788-0 36641429 PMC9840260

[23] Wanjau MN Möller H Haigh F Milat A Hayek R Lucas P . The potential impact of physical activity on the burden of osteoarthritis and low back pain in Australia: a systematic review of reviews and life table analysis. J Phys Act Health. (2023) 20:690–701. doi: 10.1123/jpah.2022-0541 37268300

[24] Wu R-Y Pan R-H Wu C-Y Chan C-L Yeh H-J. Association between weather and utilisation of physical therapy in patients with osteoarthritis: a case-crossover study. BMC Musculoskelet. Disord. (2022) 23:269. doi: 10.1186/s12891-022-05233-9 35305583 PMC8933890

[25] Vrouva S Sopidou V Sifakis E Ntoulaveris I Papamarkos G Tse G . Chronic sufferers and environmental conditions. Safety. (2023) 9:85. doi: 10.3390/safety9040085

[26] Thetkathuek A Kongsombatsuk M Nakyai T Polyong CP. Access to health services and factors affecting musculoskeletal disorders among outdoor pollution workers following Sustainable Development Goals: a weakness in Thailand. Global Health J. (2025) 9:85–93. doi: 10.1016/j.glohj.2025.06.002

[27] GBD 2021 Diseases and Injuries Collaborators. Global incidence, prevalence, years lived with disability (YLDs), disability-adjusted life-years (DALYs), and healthy life expectancy (HALE) for 371 diseases and injuries in 204 countries and territories and 811 subnational locations, 1990-2021: a systematic analysis for the Global Burden of Disease Study 2021. Lancet. (2024) 403:2133–61. doi: 10.1016/S0140-6736(24)00757-8 38642570 PMC11122111

[28] McConaghy K Klika AK Apte SS Erdemir A Derwin K Piuzzi NS. A call to action for musculoskeletal research funding: the growing economic and disease burden of musculoskeletal conditions in the United States is not reflected in musculoskeletal research funding. J Bone Joint Surg Am. (2023) 105:492–8. doi: 10.2106/JBJS.22.00693 36574617

[29] Institute for Health Metrics and Evaluation. Musculoskeletal disorders – Level 2 disease. (2024). Available online at: https://www.healthdata.org/research-analysis/diseases-injuries-risks/factsheets/2021-musculoskeletal-disorders-level-2-disease (Accessed March 20, 2025).

[30] Menne MJ Durre I Vose RS Gleason BE Houston TG. An overview of the global historical climatology network-daily database. J Atmos Ocean Technol. (2012) 29:897–910. doi: 10.1175/JTECH-D-11-00103.1

[31] Zhan Y Gu H Li X. Study on association factors of intestinal infectious diseases based-Bayesian spatio-temporal model. BMC Infect Dis. (2023) 23:720. doi: 10.1186/s12879-023-08665-3 37875791 PMC10598920

[32] Puvvula J Abadi AM Conlon KC Rennie JJ Herring SC Thie L . Estimating the burden of heat-related illness morbidity attributable to anthropogenic climate change in North Carolina. GeoHealth. (2022) 6:e2022GH000636. doi: 10.1029/2022GH000636 36439028 PMC9685474

[33] Obradovich N Migliorini R Mednick SC Fowler JH. Nighttime temperature and human sleep loss in a changing climate. Sci Adv. (2017) 3:e1601555. doi: 10.1126/sciadv.1601555 28560320 PMC5446217

[34] Devleesschauwer B Charalampous P Gorasso V Assunção R Hilderink H Idavain J . Standardised reporting of burden of disease studies: the STROBOD statement. Popul Health Metr. (2024) 22:28. doi: 10.1186/s12963-024-00347-9 39375690 PMC11459887

[35] Jin Y Guo C Abbasian M Abbasifard M Abbott JH Abdullahi A . Global pattern, trend, and cross-country inequality of early musculoskeletal disorders from 1990 to 2019, with projection from 2020 to 2050. Med. (2024) 5:943–62.e6. doi: 10.1016/j.medj.2024.04.009 38834074 PMC11321819

[36] Jena AB Olenski AR Molitor D Miller N. Association between rainfall and diagnoses of joint or back pain: retrospective claims analysis. BMJ. (2017) 359:j5326. doi: 10.1136/bmj.j5326 29237605 PMC5728253

[37] Telfer S Obradovich N. Local weather is associated with rates of online searches for musculoskeletal pain symptoms. PLoS ONE. (2017) 12:e0181266. doi: 10.1371/journal.pone.0181266 28792953 PMC5549896

[38] Park KY Kim HJ Ahn HS Yim S-Y Jun J-B. Association between acute gouty arthritis and meteorological factors: an ecological study using a systematic review and meta-analysis. Semin Arthritis Rheum. (2017) 47:369–75. doi: 10.1016/j.semarthrit.2017.05.006 28583691

[39] Cuenca-Zaldívar JN Del Corral-Villar C García-Torres S Araujo-Zamora R Gragera-Peña P Martínez-Lozano P . Fourteen-year retrospective cohort study on the impact of climatic factors on chronic musculoskeletal pain: a Spanish primary care analysis. Int J Rheum Dis. (2025) 28:e70125. doi: 10.1111/1756-185X.70125 40040581

[40] Fu K Metcalf B Bennell KL Zhang Y Deveza LA Robbins SR . Association of weather to the risk of hip osteoarthritis pain exacerbations. Osteoarthr Cartil. (2019) 27:S249. doi: 10.1016/j.joca.2019.02.615

[41] Lee Y-H Chung J-W. Climate temperature and seasonal influences on the prevalence of temporomandibular disorders in South Korea. Sci Rep. (2024) 14:10974. doi: 10.1038/s41598-024-61829-2 38744911 PMC11094084

[42] Hu X Fu H Zhang L Zhang Q Xu T Chen Y . Effect of elevated temperatures on inflammatory cytokine release: an in vitro and population-based study. Environ Health. (2024) 2:721–8. doi: 10.1021/envhealth.4c00059 39474437 PMC11503718

[43] Rajaram A Ioussoufovitch S Morrison LB St Lawrence K Lee TY Bureau Y . Joint blood flow is more sensitive to inflammatory arthritis than oxyhemoglobin, deoxyhemoglobin, and oxygen saturation. Biomed Opt Express. (2016) 7:3843–54. doi: 10.1364/BOE.7.003843 27867697 PMC5102556

[44] Farbu EH Höper AC Reierth E Nilsson T Skandfer M. Cold exposure and musculoskeletal conditions; a scoping review. Front Physiol. (2022) 13:934163. doi: 10.3389/fphys.2022.934163 36117709 PMC9475294

[45] Smedslund G Hagen KB. Does rain really cause pain? A systematic review of the associations between weather factors and severity of pain in people with rheumatoid arthritis. Eur J Pain. (2011) 15:5–10. doi: 10.1016/j.ejpain.2010.05.003 20570193

[46] Deall C Majeed H. Effect of cold weather on the symptoms of arthritic disease: a review of the literature. J Gen Pract. (2016) 4:1000275. doi: 10.4172/2329-9126.1000275

[47] Horvath G Nagy K Tuboly G Nagy E. Pain and weather associations – Action mechanisms; personalized profiling. Brain Res Bull. (2023) 200:110696. doi: 10.1016/j.brainresbull.2023.110696 37391130

[48] Farbu EH Rypdal M Skandfer M Steingrímsdóttir ÓA Brenn T Stubhaug A . To tolerate weather and to tolerate pain: two sides of the same coin? The Tromsø Study 7. Pain. (2022) 163:878–86. doi: 10.1097/j.pain.0000000000002437 34510136 PMC9009320

[49] Fagerlund AJ Iversen M Ekeland A Moen CM Aslaksen PM. Blame it on the weather? The association between pain in fibromyalgia, relative humidity, temperature and barometric pressure. PLoS ONE. (2019) 14:e0216902. doi: 10.1371/journal.pone.0216902 31075151 PMC6510434

[50] Schultz DM Beukenhorst AL Yimer BB Cook L Pisaniello HL House T . Weather patterns associated with pain in chronic-pain sufferers. Bull Am Meteorol Soc. (2020) 101:E555–66. doi: 10.1175/BAMS-D-19-0265.1

[51] Kondo Y Abe S Toko H Hirota T Takahashi H Shimizu M . Effect of climatic environment on immunological features of rheumatoid arthritis. Sci Rep. (2023) 13:1304. doi: 10.1038/s41598-022-27153-3 36693893 PMC9873807

[52] Van Der Lans AA Boon MR Haks MC Quinten E Schaart G Ottenhoff TH . Cold acclimation affects immune composition in skeletal muscle of healthy lean subjects. Physiol Rep. (2015) 3:e12394. doi: 10.14814/phy2.12394 26149277 PMC4552515

