# Supplementary material for: Effects of meteorological variability on the burden of musculoskeletal disorders among people aged 55 and above in the United States: a secondary analysis of the Global Burden of Disease Study 2021
Source: Front Public Health. 2026 Mar 17;14:1630531. doi: 10.3389/fpubh.2026.1630531 (PMC13036215; doi:10.3389/fpubh.2026.1630531)
Supplement: Supplementary file 1 [file Data_Sheet_1.docx]

**Supplementary Material 1-14**

**Contents**

[1. The STROBOD statement checklist (1) 1](#_Toc365687877)

[2. Behavioral and Healthcare Access Covariates from BRFSS 3](#_Toc17951025)

[3. The case definitions of GBD musculoskeletal causes (2) 4](#_Toc1055166595)

[4. Meteorological data preprocessing and covariates recalling 5](#_Toc265005239)

[5. Spatial and temporal trend of included meteorological variables 11](#_Toc61967995)

[6. The percentages and rates of specific-disorder burden in 2021 13](#_Toc2114006817)

[7. National-level temporal trend (AAPC) of MSK DALYs rate 15](#_Toc2143117351)

[8. Correlations between Meteorological factors and Burden of MSKs 16](#_Toc1777590773)

[9. Model comparisons for females and males (Model A-D) 18](#_Toc175624747)

[10. Model estimates for females and males (Model D) 18](#_Toc1082591851)

[11. Plots of GAM smooth terms for females and males (Model D) 18](#_Toc1639782373)

[12. Model estimates for sensitivity analysis (Sensitivity Model S1-2) 18](#_Toc1164701060)

[13. Plots of GAM smooth terms for sensitivity analysis (Sensitivity Model S1-2; both sexes, females and males) 18](#_Toc817273015)

[14. Plots of derivative analyses outputs (Model D; both sexes, females and males) 18](#_Toc602156893)

### The STROBOD statement checklist (1)

**Checklist of items that should be included in reports of cross-sectional studies.** Give information separately for exposed and unexposed groups. Note: An Explanation and Elaboration article discusses each checklist item and gives methodological background and published examples of transparent reporting. The STROBE checklist is best used in conjunction with this article (freely available on the Web sites of PLoS Medicine at <http://www.plosmedicine.org/> , Annals of Internal Medicine at http://www.annals.org/, and Epidemiology at <http://www.epidem.com/> ). Information on the STROBE Initiative is available at [www.strobe-statement.org](http://www.strobe-statement.org).

**Supplementary Material Table 1. STROBE Statement**

|  | Item No | Recommendation |  |
| --- | --- | --- | --- |
| **Title and abstract** | 1 | (*a*) Indicate the study’s design with a commonly used term in the title or the abstract | √ |
|  |  | (*b*) Provide in the abstract an informative and balanced summary of what was done and what was found | √ |
| Introduction | | |  |
| Background/rationale | 2 | Explain the scientific background and rationale for the investigation being reported | √ |
| Objectives | 3 | State specific objectives, including any prespecified hypotheses | √ |
| Methods | | |  |
| Study design | 4 | Present key elements of study design early in the paper | √ |
| Setting | 5 | Describe the setting, locations, and relevant dates, including periods of recruitment, exposure, follow-up, and data collection | √ |
| Participants | 6 | (*a*) Give the eligibility criteria, and the sources and methods of selection of participants | - |
| Variables | 7 | Clearly define all outcomes, exposures, predictors, potential confounders, and effect modifiers. Give diagnostic criteria, if applicable | √ |
| Data sources/ measurement | 8* | For each variable of interest, give sources of data and details of methods of assessment (measurement). Describe comparability of assessment methods if there is more than one group | √ |
| Bias | 9 | Describe any efforts to address potential sources of bias | √ |
| Study size | 10 | Explain how the study size was arrived at | √ |
| Quantitative variables | 11 | Explain how quantitative variables were handled in the analyses. If applicable, describe which groupings were chosen and why | √ |
| Statistical methods | 12 | (*a*) Describe all statistical methods, including those used to control for confounding | √ |
|  |  | (*b*) Describe any methods used to examine subgroups and interactions | √ |
|  |  | (*c*) Explain how missing data were addressed | √ |
|  |  | (*d*) If applicable, describe analytical methods taking account of sampling strategy | - |
|  |  | (*e*) Describe any sensitivity analyses | √ |
| Results | | |  |
| Participants | 13* | (a) Report numbers of individuals at each stage of study—eg numbers potentially eligible, examined for eligibility, confirmed eligible, included in the study, completing follow-up, and analysed | - |
|  |  | (b) Give reasons for non-participation at each stage | - |
|  |  | (c) Consider use of a flow diagram | - |
| Descriptive data | 14* | (a) Give characteristics of study participants (eg demographic, clinical, social) and information on exposures and potential confounders | - |
|  |  | (b) Indicate number of participants with missing data for each variable of interest | - |
| Outcome data | 15* | Report numbers of outcome events or summary measures | √ |
| Main results | 16 | (*a*) Give unadjusted estimates and, if applicable, confounder-adjusted estimates and their precision (eg, 95% confidence interval). Make clear which confounders were adjusted for and why they were included | √ |
|  |  | (*b*) Report category boundaries when continuous variables were categorized | - |
|  |  | (*c*) If relevant, consider translating estimates of relative risk into absolute risk for a meaningful time period | - |
| Other analyses | 17 | Report other analyses done—eg analyses of subgroups and interactions, and sensitivity analyses | √ |
| Discussion | | |  |
| Key results | 18 | Summarise key results with reference to study objectives | √ |
| Limitations | 19 | Discuss limitations of the study, taking into account sources of potential bias or imprecision. Discuss both direction and magnitude of any potential bias | √ |
| Interpretation | 20 | Give a cautious overall interpretation of results considering objectives, limitations, multiplicity of analyses, results from similar studies, and other relevant evidence | √ |
| Generalisability | 21 | Discuss the generalisability (external validity) of the study results | √ |
| Other information | | |  |
| Funding | 22 | Give the source of funding and the role of the funders for the present study and, if applicable, for the original study on which the present article is based | - |

### Behavioral and Healthcare Access Covariates from BRFSS

State-level behavioral risk factors and healthcare access indicators were obtained from the Behavioral Risk Factor Surveillance System (BRFSS), a nationally representative, continuous, state-based telephone survey conducted by the Centers for Disease Control and Prevention (CDC) (<https://www.cdc.gov/brfss/brfssprevalence/index.html> ). Publicly available annual estimates stratified by state, sex, and calendar year were extracted for all study years. Two variables were used as behavioral and metabolic confounders in the main models:

- **Obesity prevalence** was defined as the proportion of adults aged ≥18 years with a self-reported body mass index (BMI) ≥30 kg/m².
- **Current smoking rate** was defined as the proportion of adults who reported smoking cigarettes every day or on some days at the time of the survey.

Healthcare access was characterized using five BRFSS-derived indicators, reflecting complementary dimensions of coverage, financial barriers, continuity of care, and utilization:

- **Healthcare coverage:** proportion of adults who reported having any kind of health care coverage.
- **Financial barrier to care:** proportion of adults reporting that they were unable to see a doctor at least once in the past 12 months because of cost.
- **Routine checkup utilization:** proportion of adults reporting having a routine medical checkup within the past 12 months.
- **Personal healthcare provider availability:** proportion of adults reporting not having a personal doctor or healthcare provider.
- **Under-65 coverage index:** BRFSS composite indicator summarizing health insurance coverage among adults aged 18-64 years, calculated from one or more survey questions.

For state-year-sex records with missing values (specifically New Jersey in 2019 and Florida in 2021), all behavioral and healthcare access variables were imputed using nearest-neighbor temporal interpolation within each state–sex stratum, leveraging values from adjacent survey years to preserve within-state temporal continuity.

To reduce dimensionality and mitigate multicollinearity among the five healthcare access indicators, we conducted **principal component analysis (PCA)** using the correlation matrix of all standardized variables. PCA yielded five orthogonal components, of which the first two were retained for sensitivity analyses based on variance explained and substantive interpretability.

- **The first principal component (PC1),** explaining **48.1% of the total variance**, represented **overall healthcare accessibility and social medical security**, characterized by high positive loadings on health insurance coverage and recent routine checkup utilization, and strong inverse loadings on cost-related barriers to medical care. Higher PC1 scores therefore indicated better structural access to healthcare and stronger financial protection.
- **The second principal component (PC2),** accounting for an additional **23.5% of the variance**, primarily captured **healthcare utilization patterns reflecting preventive checkup behavior versus cost-related care avoidance**, with contrasting loadings on routine checkup frequency and financial access barriers. Higher PC2 values reflected more active preventive service utilization under relatively lower cost pressure.

Together, PC1 and PC2 explained **71.5% of the total variance** in healthcare access measures and were jointly interpreted as complementary dimensions of healthcare access structure and utilization behavior. In sensitivity analyses, PC1 and PC2 were jointly incorporated to evaluate the robustness of meteorology-musculoskeletal disease associations to alternative representations of healthcare access. All PCA-derived component scores were standardized before model entry.

### The case definitions of GBD musculoskeletal causes (2)

- Musculoskeletal disorders

Musculoskeletal disorders (MSKs) represent a category of conditions primarily associated with disability, and in some instances mortality, including rheumatoid arthritis, osteoarthritis, low back pain, neck pain, gout, and a broad residual category known as “other musculoskeletal disorders.”

- Rheumatoid arthritis

Rheumatoid arthritis (RA) is a systemic autoimmune disorder characterized by chronic inflammation that leads to joint pain, swelling, and deformity, frequently accompanied by systemic symptoms. Although RA can affect internal organs beyond the joints, the Global Burden of Disease (GBD) framework does not quantify these extra-articular manifestations. ICD-10 codes for rheumatoid arthritis include M05, M06, and M08, with corresponding ICD-9 codes ranging from 714.0 to 714.9.

- Osteoarthritis

The standard diagnostic definition of osteoarthritis (OA) is symptomatic osteoarthritis confirmed radiologically as Kellgren-Lawrence grades 2–4. GBD 2019 introduced two additional OA categories: hand osteoarthritis (following the same diagnostic standards for individual hand joints) and osteoarthritis of joints other than the hand, hip, knee, or spine, also using the same criteria.

Grade 2 symptomatic OA is characterized by at least one clear osteophyte in the affected joint, with pain lasting at least one month within the past 12 months. Grade 3–4 symptomatic OA requires the presence of osteophytes and joint-space narrowing, with deformities occurring in grade 4, and similarly requires pain persisting for at least one month in the past year.

- Low back pain

Low back pain (LBP) is defined as pain in the lower back region—with or without referred pain radiating into one or both lower limbs—lasting at least one day. The anatomical definition of "low back" covers the posterior area from the lower edge of the twelfth ribs to the lower gluteal folds.

The relevant ICD-10 codes for low back pain are M54.3, M54.4, and M54.5; the corresponding ICD-9 code is 724.

- Neck pain

Neck pain involves discomfort localized to the neck, with or without referred pain extending into the upper limbs, lasting a minimum of one day. The ICD-10 code for neck pain is M54.2, with the corresponding ICD-9 code being 723.1.

- Gout

Gout is a rheumatic disorder caused by the deposition of monosodium urate (MSU) crystals in joint fluid and tissues due to elevated urate concentrations in extracellular fluids, triggering inflammatory responses. GBD adopts the American College of Rheumatology’s ARA 1977 survey criteria for gout diagnosis. ICD-10 code for gout is M10, with the corresponding ICD-9 code being 274.

- Other musculoskeletal disorders

The category "other musculoskeletal disorders" comprises a heterogeneous group of musculoskeletal and connective tissue conditions that are not classified as rheumatoid arthritis, osteoarthritis, low back pain, neck pain, or gout. These disorders include selected soft tissue disorders, non-specific rheumatologic and musculoskeletal conditions, and other residual musculoskeletal diseases and do not include chronic complications resulting from injuries (3). The majority of mortality within this residual group is attributed to autoimmune disorders such as systemic lupus erythematosus and systemic sclerosis.

### Meteorological data preprocessing and covariates recalling

We obtained data on 8 meteorological elements from the U.S. Historical Climatology Network (file “ghcnd_hcn.tar.gz”) for the period 1 January 1990 to 31 December 2021 (4). The original element codes are as follows:

- TMAX = Maximum temperature (tenths of degrees C)
- TMIN = Minimum temperature (tenths of degrees C)
- TAVG = Average daily temperature (tenths of degrees C)

Note that TAVG is computed in a variety of ways including traditional fixed hours of the day other than solely computed as (TMAX+TMIN)/2.0.

- RHAV = Average relative humidity for the day (percent)
- RHMN = Minimum relative humidity for the day (percent)
- RHMX = Maximum relative humidity for the day (percent)
- AWND = Average daily wind speed (tenths of meters per second)
- ASTP = Average Station Level Pressure for the day (hPa * 10)

These elements are recorded daily at a station level with quality control. The yearly summaries of each meteorological variables were computed as:

- TMAX_ANN = max (TMAX, na.rm = TRUE), yearly maximum temperature
- TMIN_ANN = min (TMIN, na.rm = TRUE), yearly minimum temperature
- RHMX_ANN = max (RHMX, na.rm = TRUE), yearly maximum relative humidity
- RHMN_ANN = min (RHMN, na.rm = TRUE), yearly minimum relative humidity
- MEAN_TAVG = mean (TAVG, na.rm = TRUE), yearly average daily temperature
- MEAN_RRAV = mean (RHAV, na.rm = TRUE), yearly average daily relative humidity
- MEAN_AWND = mean (AWND, na.rm = TRUE), yearly average daily wind speed
- MEAN_ASTP = mean (ASTP, na.rm = TRUE), yearly average daily barometric pressure

Following quality control, we removed all invalid or missing daily values and retained only genuine observations. Therefore, annual summaries were computed directly from valid daily records. We did not apply multiple imputation, as the missingness in GHCN-Daily is largely structural (i.e., many stations never reported certain variables, or entire years were excluded by QC flags), which does not meet the assumptions of imputation and risks introducing synthetic, non-observed values. Instead, we restricted analyses to elements with valid reporting. According to GHCN-Daily README file (Version 3.31, available at <https://www1.ncdc.noaa.gov/pub/data/ghcn/daily/readme.txt>), Core variables (TMAX, TMIN) were consistently available across most U.S. stations, while extended variables (TAVG, RHAV, RHMX, RHMN, AWND, ASTP) were only available from a limited subset of stations, largely after 2013 (see Supplementary Material Table 2). Accordingly, the study period was restricted to 2013-2021.

Incorporating the multi-polygons and station metadata, we applied Inverse Distance Weighting (IDW) interpolation, a widely used spatial interpolation method, to generate state-level averages based solely on nearby valid observations, to produce annual summaries of these meteorological elements for the continental states during the study period. Analyses were limited to the 49 continental U.S. states, excluding Alaska, Hawaii, Puerto Rico, and other territories, to reduce heterogeneity from extreme meteorologicals and socio-economic contexts.

Before we fit the interpolated variables into the models, we obtained the annual ranges of temperatures and relative humidity by:

- TRAN_ANN = abs (TMAX_ANN - TMIN_ANN)
- RRAV_ANN = abs (RHMX_ANN - RHMN_ANN)

Finally, six meteorological variables (with under-5 VIF values) were further rescaled and modelled in the final analysis. All the covariates were presented as in Supplementary Material Table 3.To improve interpretability, we have provided supplementary conversion tables linking standardized meteorological units to their corresponding physical scales (Supplementary Material Table 4 and Supplementary Material Figure 1). Nevertheless, it is important to note that the present analysis is fundamentally burden-oriented and population-based, rather than focused on individual-level exposure thresholds. Several meteorological indicators, particularly annual temperature range, were derived from state-level spatially aggregated annual extremes and thus reflect regional meteorological variability rather than local or individual thermal exposure. Consequently, the absolute magnitude of these values should not be interpreted as direct physiological thresholds for disease onset or exacerbation.

**Supplementary Material Table 2. The number of stations (post-QC) across U.S. continental states.**

| Year | TMAX | TMIN | TAVG | RHMX | RHMN | RHAV | AWND | ASTP |
| --- | --- | --- | --- | --- | --- | --- | --- | --- |
| 1990 | 1205 | 1205 | - | - | - | - | 63 | - |
| 1991 | 1199 | 1198 | - | - | - | - | 68 | - |
| 1992 | 1199 | 1199 | - | - | - | - | 69 | - |
| 1993 | 1200 | 1200 | 1 | - | - | - | 66 | - |
| 1994 | 1199 | 1199 | 1 | - | - | - | 67 | - |
| 1995 | 1195 | 1195 | 1 | - | - | - | 67 | - |
| 1996 | 1189 | 1189 | 1 | - | - | - | 67 | - |
| 1997 | 1179 | 1179 | - | - | - | - | 68 | - |
| 1998 | 1182 | 1182 | 58 | - | - | - | 68 | - |
| 1999 | 1181 | 1181 | 60 | - | - | - | 67 | - |
| 2000 | 1181 | 1181 | 62 | - | - | - | 67 | - |
| 2001 | 1172 | 1172 | 64 | - | - | - | 44 | - |
| 2002 | 1164 | 1164 | 64 | - | - | - | 44 | - |
| 2003 | 1156 | 1156 | 60 | - | - | - | 43 | - |
| 2004 | 1143 | 1143 | 61 | - | - | - | 47 | - |
| 2005 | 1135 | 1135 | 61 | - | - | - | 48 | - |
| 2006 | 1124 | 1124 | - | 55 | 55 | 55 | 42 | 55 |
| 2007 | 1105 | 1105 | - | 55 | 55 | 55 | 41 | 55 |
| 2008 | 1098 | 1098 | - | 55 | 55 | 55 | 41 | 55 |
| 2009 | 1076 | 1076 | - | 55 | 55 | 55 | 49 | 55 |
| 2010 | 1051 | 1050 | - | 56 | 56 | 56 | 50 | 56 |
| 2011 | 1036 | 1036 | - | 55 | 55 | 55 | 56 | 55 |
| 2012 | 1008 | 1008 | - | 58 | 58 | 57 | 59 | 56 |
| 2013 | 969 | 969 | 38 | 57 | 57 | 57 | 60 | 56 |
| 2014 | 949 | 948 | 38 | 56 | 56 | 56 | 59 | 55 |
| 2015 | 933 | 933 | 38 | 57 | 57 | 57 | 61 | 56 |
| 2016 | 920 | 920 | 38 | 58 | 58 | 57 | 60 | 56 |
| 2017 | 909 | 909 | 38 | 58 | 58 | 57 | 60 | 57 |
| 2018 | 882 | 882 | 38 | 58 | 58 | 57 | 60 | 57 |
| 2019 | 868 | 867 | 38 | 57 | 57 | 57 | 58 | 57 |
| 2020 | 863 | 863 | 38 | 56 | 56 | 55 | 58 | 55 |
| 2021 | 853 | 852 | 38 | 56 | 56 | 55 | 60 | 55 |
| **Note:** **QC is referred as built-in quality control in the GHCN-Daily dataset.** | | | | | | | | |

**Supplementary Material Table 3. Covariates reprocessing outlook.**

| **Covariates** | **Definitions** | **Scaling method** | **VIF_M0** | **VIF_M1** | **VIF_S1** | **VIF_S2** |
| --- | --- | --- | --- | --- | --- | --- |
| SDI | Socio-Demographic Indices | standardized scaling | 5.431 | 3.988 | - | - |
| Obesity Prevalence | Proportion of people with BMI ≥30 | standardized scaling | 2.025 | 1.844 | 1.925 | 1.489 |
| Smoking Rate | Proportion of current smoker | standardized scaling | 3.358 | 3.353 | 1.983 | 1.920 |
| Population density | Population of adults aged  55 and over | logarithmic scaling (natural log) | 5.263 | - | 3.865 | - |
| Healthcare access PC1 | Structural access to healthcare and stronger financial protection | standardized scaling | - | - | - | 2.349 |
| Healthcare access PC2 | Active preventive service utilization under relatively lower cost pressure | standardized scaling | - | - | - | 2.753 |
| TRAN_ANN | Annual maximum temperature range (tenths of degrees C) | standardized scaling | 3.275 | 2.698 | 2.819 | 2.820 |
| MEAN_TAVG | Annual average daily temperature (tenths of degrees C) | standardized scaling | 2.001 | 1.887 | 1.846 | 2.050 |
| RRAV_ANN | Annual relative humidity range (percent) | standardized scaling | 2.438 | 2.366 | 2.323 | 2.373 |
| MEAN_RRAV | Annual average daily relative humidity (percent) | logarithmic scaling (natural log) | 2.259 | 2.255 | 2.150 | 2.351 |
| MEAN_ASTP | Annual average barometric pressure (hPa * 10) | logarithmic scaling (natural log) | 4.446 | 3.763618 | 3.764011 | 3.763771 |
| MEAN_AWND | Annual average wind speed (tenths of meters per second) | logarithmic scaling (natural log) | 2.043 | 1.996905 | 1.996389 | 1.997471 |

**VIF_M0** including SDI, Obesity Prevalence, Smoking Rate, Population density and six meteorological factors;

**VIF_M1** including SDI, Obesity Prevalence, Smoking Rate and six meteorological factors;

**VIF_S1** including Obesity Prevalence, Smoking Rate, Population density and six meteorological factors;

**VIF_S2** including Obesity Prevalence, Smoking Rate, Healchcare Access Principal Components and six meteorological factors**.**

**Supplementary Material Table 4. Comparisons of meteorological variables units before and after rescaling.**

| Variable | Scaled Units | Original Units 1 | Original Units 2 | Quantile |
| --- | --- | --- | --- | --- |
| TRAN_ANN (tenths of °C) | -2 | 366.83 | 36.68 °C | - |
| TRAN_ANN (tenths of °C) | -1 | 440.18 | 44.02 °C | - |
| TRAN_ANN (tenths of °C) | 0 | 513.54 | 51.35 °C | - |
| TRAN_ANN (tenths of °C) | 1 | 586.90 | 58.69 °C | - |
| TRAN_ANN (tenths of °C) | 2 | 660.26 | 66.03 °C | - |
| MEAN_TAVG (tenths of °C) | -2 | 42.47 | 4.25 °C | - |
| MEAN_TAVG (tenths of °C) | -1 | 87.88 | 8.79 °C | - |
| MEAN_TAVG (tenths of °C) | 0 | 133.29 | 13.33 °C | - |
| MEAN_TAVG (tenths of °C) | 1 | 178.70 | 17.87 °C | - |
| MEAN_TAVG (tenths of °C) | 2 | 224.11 | 22.41 °C | - |
| RRAV_ANN (percent) | -2 | 74.98 | 74.98 % | - |
| RRAV_ANN (percent) | -1 | 79.71 | 79.71 % | - |
| RRAV_ANN (percent) | 0 | 84.44 | 84.44 % | - |
| RRAV_ANN (percent) | 1 | 89.16 | 89.16 % | - |
| RRAV_ANN (percent) | 2 | 93.89 | 93.89 % | - |
| MEAN_RRAV (percent) | 3.845 | 46.76 | 46.76 % | 0.05 |
| MEAN_RRAV (percent) | 4.134 | 62.45 | 62.45 % | 0.25 |
| MEAN_RRAV (percent) | 4.203 | 66.88 | 66.88 % | 0.50 |
| MEAN_RRAV (percent) | 4.238 | 69.27 | 69.27 % | 0.75 |
| MEAN_RRAV (percent) | 4.290 | 73.00 | 73.00 % | 0.95 |
| MEAN_AWND (tenths of m/s) | 3.279 | 26.56 | 2.66 m/s | 0.05 |
| MEAN_AWND (tenths of m/s) | 3.387 | 29.58 | 2.96 m/s | 0.25 |
| MEAN_AWND (tenths of m/s) | 3.492 | 32.84 | 3.28 m/s | 0.50 |
| MEAN_AWND (tenths of m/s) | 3.639 | 38.04 | 3.80 m/s | 0.75 |
| MEAN_AWND (tenths of m/s) | 3.791 | 44.32 | 4.43 m/s | 0.95 |
| MEAN_ASTP (hPa * 10) | 8.428 | 4574.97 | 45749.73 hPa | 0.05 |
| MEAN_ASTP (hPa * 10) | 8.504 | 4934.23 | 49342.28 hPa | 0.25 |
| MEAN_ASTP (hPa * 10) | 8.543 | 5131.73 | 51317.25 hPa | 0.50 |
| MEAN_ASTP (hPa * 10) | 8.556 | 5197.44 | 51974.43 hPa | 0.75 |
| MEAN_ASTP (hPa * 10) | 8.569 | 5267.40 | 52673.98 hPa | 0.95 |


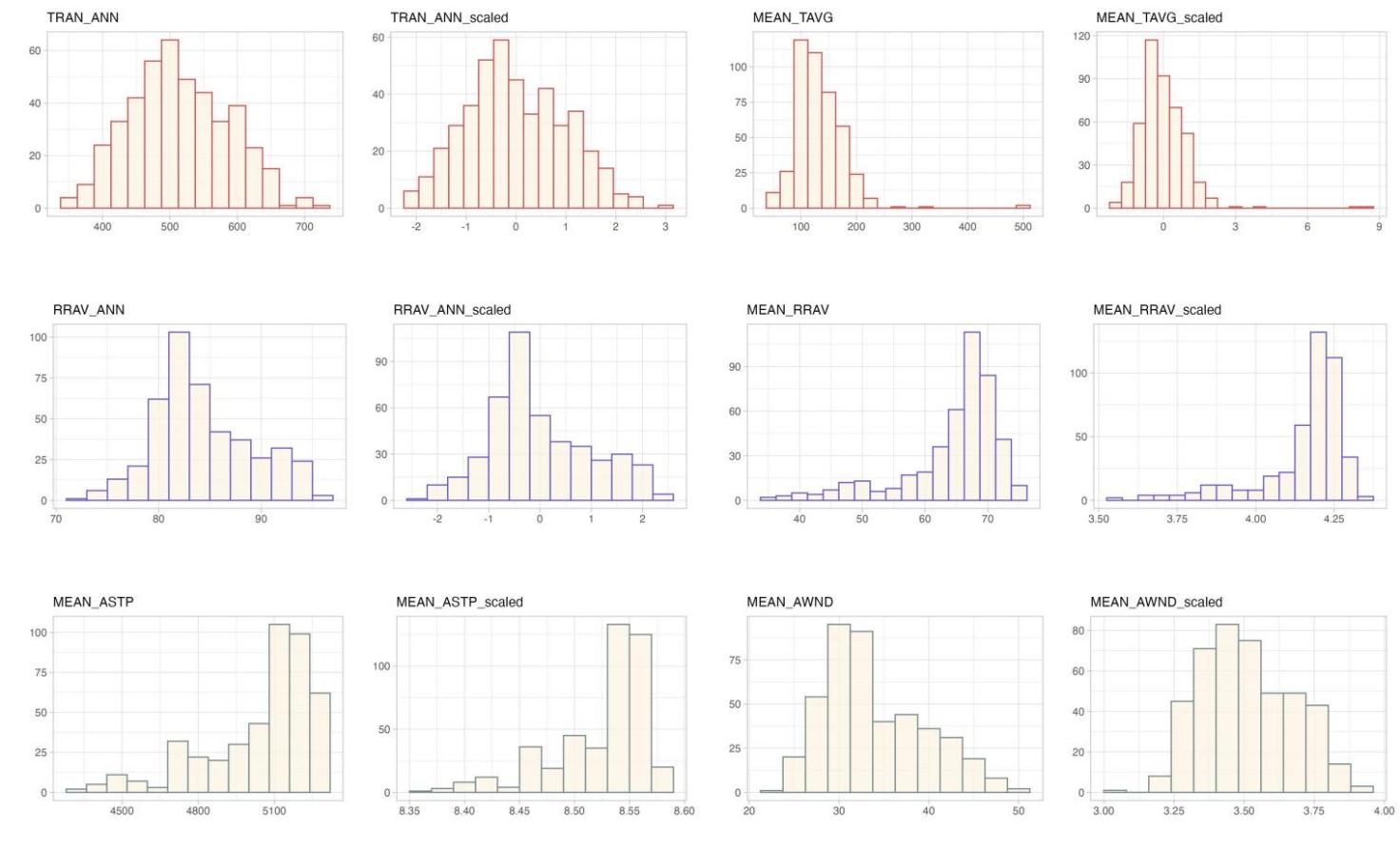
**Supplementary Material Figure 1. Comparisons of meteorological variables description before and after rescaling.** TRAN_ANN: Annual maximum temperature range; MEAN_TAVG: Annual average daily temperature; RRAV_ANN: Annual relative humidity range; MEAN_RRAV: Annual average daily relative humidity; MEAN_ASTP: Annual average barometric pressure; MEAN_AWND: Annual average wind speed.

### Spatial and temporal trend of included meteorological variables

**
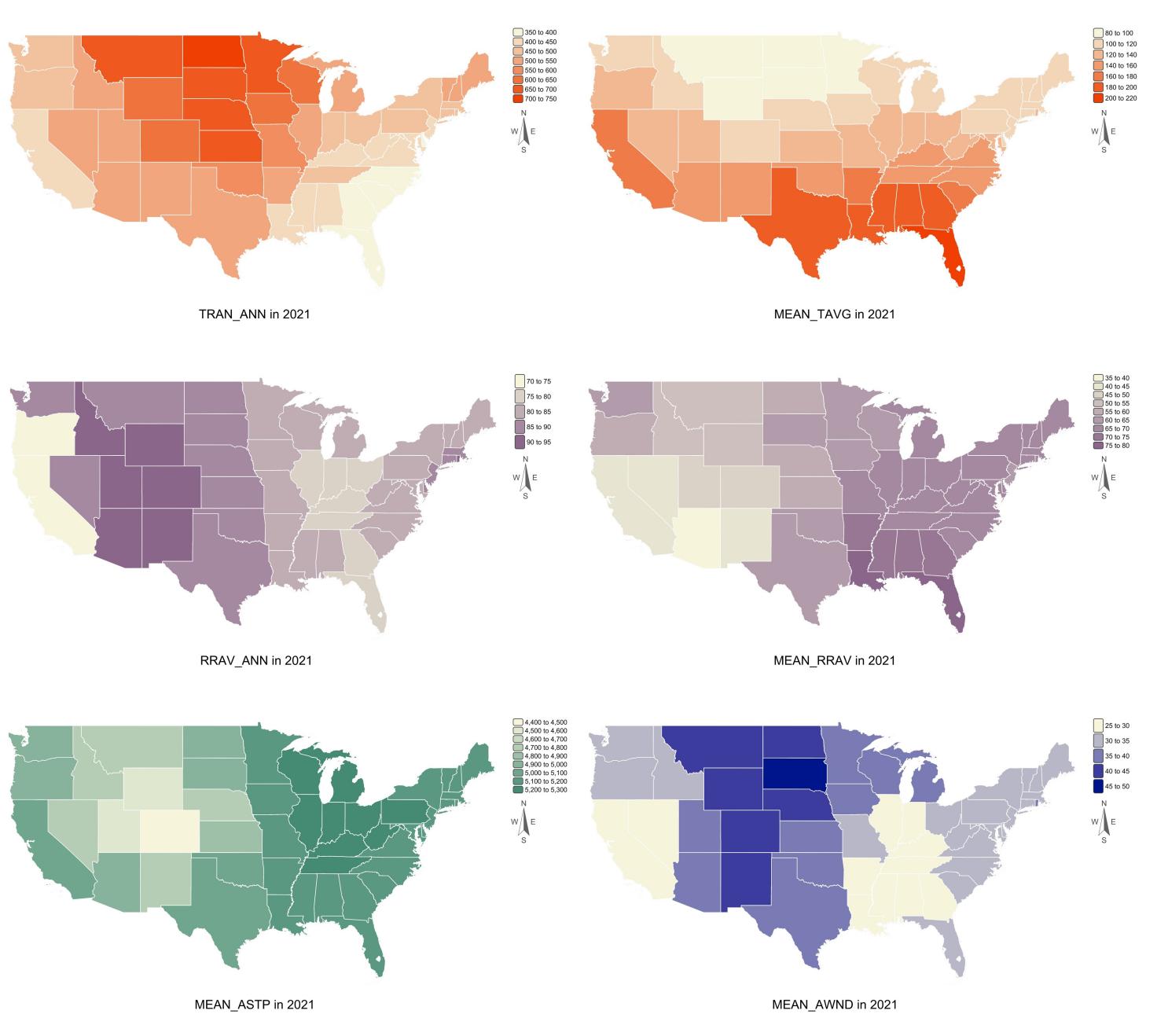
**

**Supplementary Material Figure 2.** **The nation-wide distributions of meteorological variables (2021).** The maps were plotted using the *tmap* package. There were only 49 location units mapped since Alaska, Hawaii, Fed States of Micronesia, and Puerto Rico were excluded in the final modelling analysis (for minimizing the effects of extreme outlier meteorologicals or socioeconomic contexts).

TRAN_ANN: Annual maximum temperature range; MEAN_TAVG: Annual average daily temperature; RRAV_ANN: Annual relative humidity range; MEAN_RRAV: Annual average daily relative humidity; MEAN_ASTP: Annual average barometric pressure; MEAN_AWND: Annual average wind speed.

**Supplementary Material Table 5. The states with statistically significant temporal trend**

**(from 2013 to 2021; *** P values < 0.001, ** P values < 0.01, * P values < 0.05)**

| **Meteorological variables** | **Definitions** | **States** | **AAPC (%)** | **AAPC 95% CI (%)** |
| --- | --- | --- | --- | --- |
| TRAN_ANN | Annual maximum temperature range | Idaho | -2.269 * | -4.011 to -0.494 |
|  |  | Minnesota | 0.847 * | 0.025 to 1.676 |
|  |  | North Dakota | 1.296 * | 0.130 to 2.475 |
|  | | | | |
| MEAN_TAVG | Annual average  daily temperature | Alabama | -1.504 ** | -2.509 to -0.489 |
|  |  | Kentucky | -2.945 ** | -5.000 to -0.845 |
|  |  | North Carolina | -1.999 * | -3.917 to-0.044 |
|  |  | Pennsylvania | -3.482 * | -6.335 to -0.542 |
|  |  | South Carolina | -1.422 ** | -2.450 to -0.383 |
|  |  | Tennessee | -2.083 ** | -3.271 to -0.880 |
|  | | | | |
| RRAV_ANN | Annual maximum relative humidity range | - | - | - |
|  | | | | |
| MEAN_RRAV | Annual average daily relative humidity | District of Columbia | 0.834 * | 0.144 to 1.530 |
|  |  | Hawaii | -1.883 ** | -3.107 to -0.644 |
|  |  | Maryland | 0.750 * | 0.088 to 1.416 |
|  |  | Oregon | -2.242 *** | -3.209 to -1.266 |
|  | | | | |
| MEAN_ASTP | Annual average barometric pressure | Connecticut | 0.243 *** | 0.119 to 0.366 |
|  |  | Massachusetts | 0.277 ** | 0.103 to 0.451 |
|  |  | Rhode Island | 0.374 * | 0.043 to 0.706 |
|  | | | | |
| MEAN_AWND | Annual average wind speed | Arizona | 4.409 * | 1.083 to 7.845 |
|  |  | Maine | 1.301 * | 0.290 to 2.322 |
|  |  | Massachusetts | 0.965 * | 0.108 to 1.830 |
|  |  | New Mexico | 2.548 * | 0.333 to 4.812 |
|  |  | Rhode Island | 1.094 ** | 0.377 to 1.817 |

**Note: The temporal trend was identified by the Average Annual Percent Change (AAPC) using Joinpoint analysis; Alaska, Hawaii, Fed States of Micronesia, and Puerto Rico were excluded.**

### The percentages and rates of specific-disorder burden in 2021

**
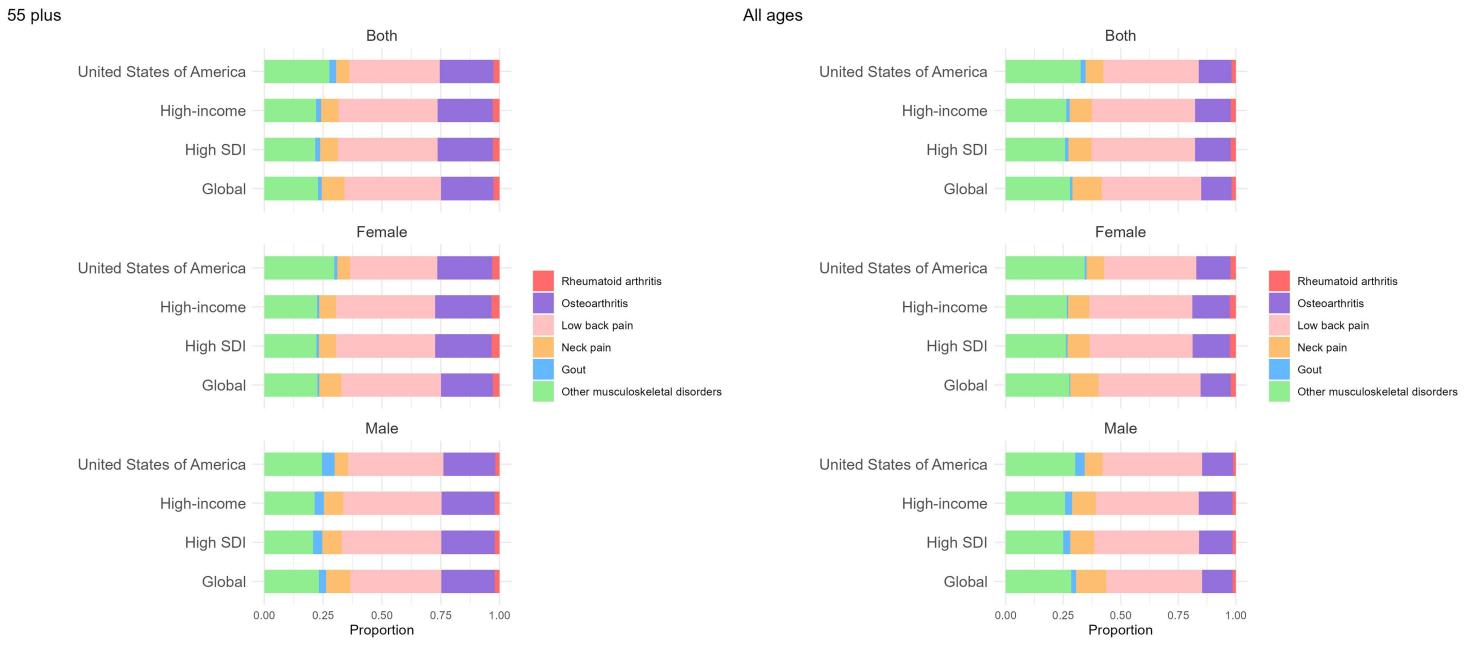
**

**Supplementary Material** **Figure 3. The percentages of disease burden caused by specific musculoskeletal (MSK) disorders among adults aged 55 and over in the United States (2021).**

The disorder-specific percentages were calculated as the percent of each MSK subtype (the level 3 cause) diving the percent of the total MSK burden (the level 2 cause). 55 plus: Percentage of DALYs (Disability-Adjusted Life-Years) estimated for adults aged 55 and over; All ages: Percentage of DALYs (Disability-Adjusted Life-Years ) estimated for the whole population.

**
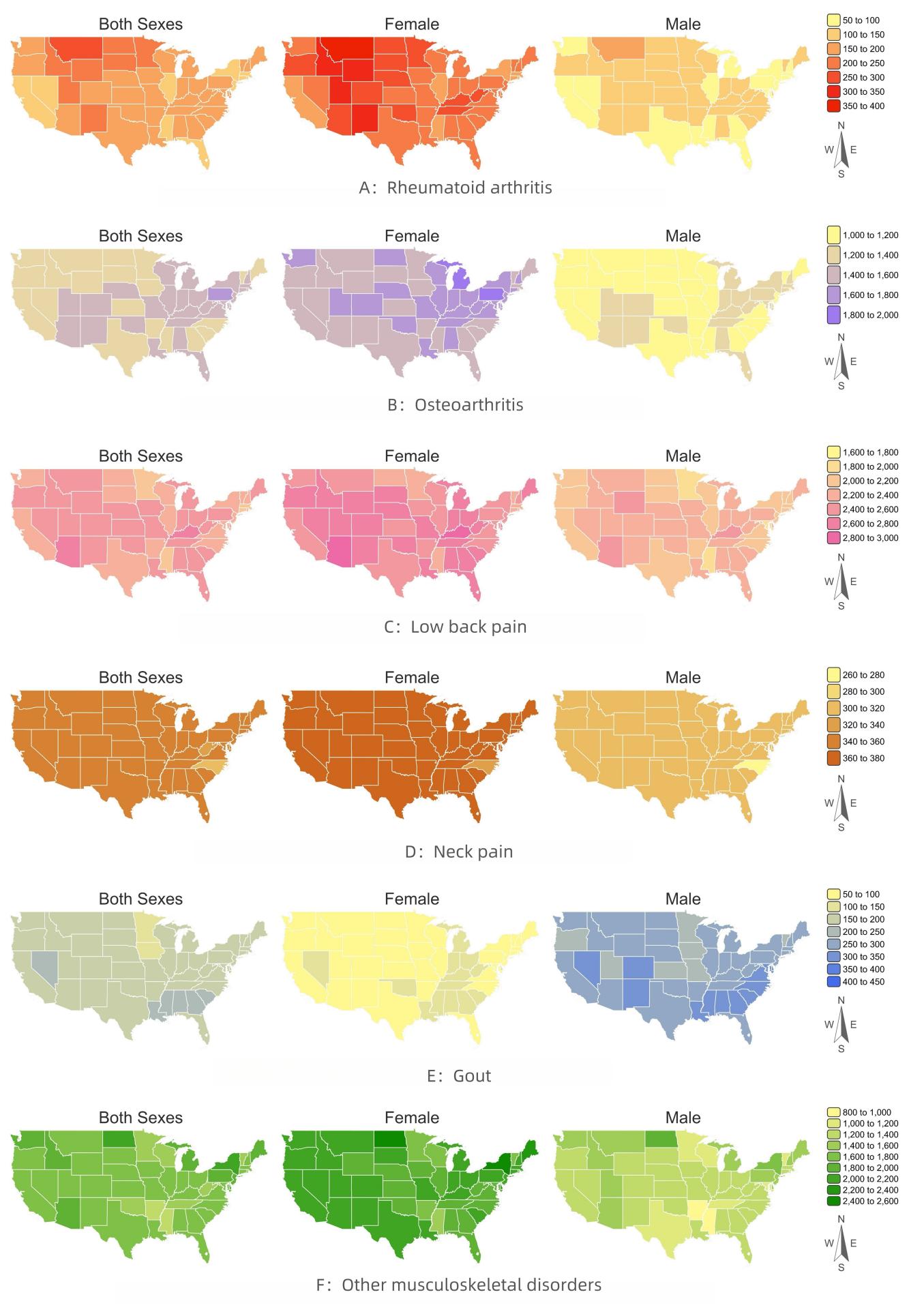
**

**Supplementary Material** **Figure 4. The state-level disease burden of six musculoskeletal (MSK) disorders among adults aged 55 and over in the United States (2021).**

The MSK burden was measured by the rates (years per 100,000 people) of DALYs (Disability-Adjusted Life-Years) attributed to six MSK subgroups. The maps were plotted using *tmap* package in R. There were only 49 location units mapped since Alaska, Hawaii, Fed States of Micronesia, and Puerto Rico were excluded in the final modelling analysis (for minimizing the effects of extreme outlier meteorologicals or socioeconomic contexts). A: rheumatoid arthritis; B: osteoarthritis; C: low back pain; D: neck pain; E: gout; F: other musculoskeletal disorders.

### National-level temporal trend (AAPC) of MSK DALYs rate

**Supplementary Material** **Table 6. The nation-level temporal trend of six musculoskeletal (MSK) disorders among adults aged 55 and over in the United States (from 2013 to 2021;** *** P values < 0.001, ** P values < 0.01, * P values < 0.05**)**

| **Sex** | **Cause** | **AAPC (%)** | **AAPC 95% CI (%)** |
| --- | --- | --- | --- |
| Both | Rheumatoid arthritis | -0.526 *** | -0.778 to -0.273 |
|  | Osteoarthritis | 1.013 *** | 0.792 to 1.234 |
|  | Low back pain | -0.044 *** | -0.062 to -0.026 |
|  | Neck pain | 0.180 *** | 0.164 to 0.196 |
|  | Gout | 0.878 *** | 0.813 to 0.943 |
|  | Other musculoskeletal disorders | 0.950 *** | 0.938 to 0.963 |
|  |  |  |  |
| Female | Rheumatoid arthritis | -0.576 *** | -0.75 to -0.401 |
|  | Osteoarthritis | 0.772 *** | 0.704 to 0.841 |
|  | Low back pain | -0.083 *** | -0.098 to -0.067 |
|  | Neck pain | 0.122 *** | 0.099 to 0.145 |
|  | Gout | 0.977 *** | 0.890 to 1.065 |
|  | Other musculoskeletal disorders | 0.773 *** | 0.719 to 0.827 |
|  |  |  |  |
| Male | Rheumatoid arthritis | -0.356 * | -0.667 to -0.044 |
|  | Osteoarthritis | 1.462 *** | 1.024 to 1.902 |
|  | Low back pain | 0.025 | -0.005 to 0.056 |
|  | Neck pain | 0.282 *** | 0.268 to 0.296 |
|  | Gout | 0.772 *** | 0.657 to 0.886 |
|  | Other musculoskeletal disorders | 1.339 *** | 1.283 to 1.396 |

**Note:** **The temporal trend was identified by the Average Annual Percent Change (AAPC) using Joinpoint analysis.**

### Correlations between Meteorological factors and Burden of MSKs

As shown in Supplementary Material Figure 5 3A-C, nearly all the included meteorological variables were significantly correlated with one or more MSK conditions, although the direction and strength of these correlations varied. For rheumatoid arthritis, both sexes demonstrated strong positive correlations with MEAN_AWND (annual average wind speed) , TRAN_ANN (annual maximum temperature range), and RRAV_ANN (annual humidity range). For other musculoskeletal disorders, males demonstrated stronger correlations compared to females with MEAN_TAVG (annual average daily temperature), TRAN_ANN , and MEAN_AWND. In contrast, gout exhibited more pronounced sex-based differences: RRAV_ANN, MEAN_RRAV (annual average daily relative humidity), and MEAN_ASTP (annual average barometric pressure) were significant only for females. In terms of osteoarthritis, females exhibited more substantial associations with meteorological variables, whereas males showed significant correlation primarily with MEAN_TAVG.

Additionally, Supplementary Material Figure 5 3D-E presented a strong positive correlation between the percentage annual changes (AAPCs) of neck pain and MEAN_RRAV. The estimated AAPC of MEAN_TAVG was positively correlated to that of osteoarthritis . Another noticeable pair of AAPCs was seen between rheumatoid arthritis and MEAN_ASTP.


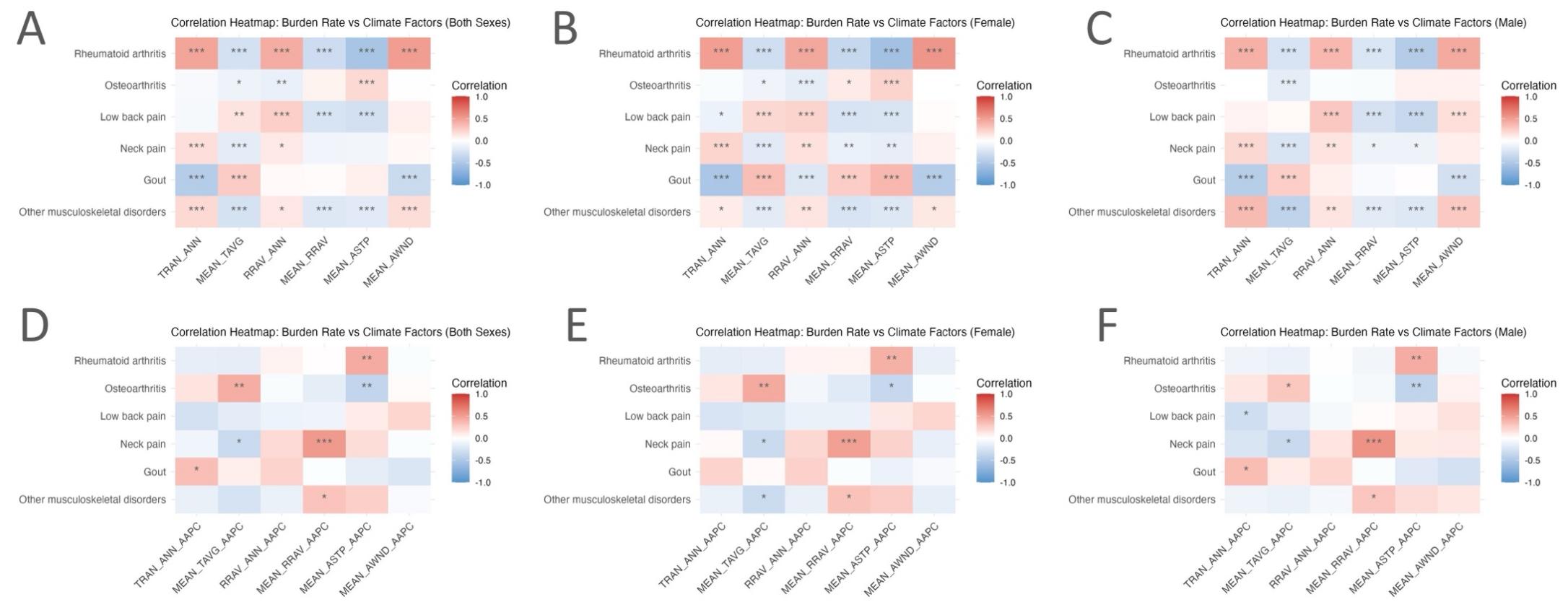


**Supplementary Material Figure 5. Unadjusted pairwise correlations between meteorological variables and MSK burden (*p<0.05, **p<0.01, ***p<0.001).** A-C: correlations between the rates of DALYs and meteorological variables; D-F: correlations between the AAPCs of the rates of DALYs and the AAPCs of meteorological variables. TRAN_ANN: Annual maximum temperature range; MEAN_TAVG: Annual average daily temperature; RRAV_ANN: Annual relative humidity range; MEAN_RRAV: Annual average daily relative humidity; MEAN_ASTP: Annual average barometric pressure; MEAN_AWND: Annual average wind speed.

### Model comparisons for females and males (Model A-D)

(see Supplementary Material Table 7 and 8)

### Model estimates for females and males (Model D)

(see Supplementary Material Table 9 and 10)

### Plots of GAM smooth terms for females and males (Model D)

(see Supplementary Material Figure 6 and 7)

### Model estimates for sensitivity analysis (Sensitivity Model S1-2)

(see Supplementary Material Table 11-13)

### Plots of GAM smooth terms for sensitivity analysis (Sensitivity Model S1-2; both sexes, females and males)

(see Supplementary Material Figure 8-13)

### Plots of derivative analyses outputs (Model D; both sexes, females and males)

(see Supplementary Material Figure 14-19)

**Supplementary Material Table 7. Comparisons of GLM and GAM model fit for females**

| **Rheumatoid arthritis** | **R^2^** | **Sigma^2^** | **ML** | **AIC** | **Osteoarthritis** | **R^2^** | **Sigma^2^** | **ML** | **AIC** |
| --- | --- | --- | --- | --- | --- | --- | --- | --- | --- |
|  |  |  |  |  |  |  |  |  |  |
| Model A | 0.094 | 1815.689 | -2279.430 | 4564.860 | Model A | 0.072 | 8728.571 | -2625.645 | 5257.291 |
| Model B | 0.604 | 803.965 | -2096.762 | 4211.524 | Model B | 0.135 | 8244.199 | -2610.022 | 5238.044 |
| Model C | 0.699 | 602.509 | -2023.544 | 4112.461 | Model C | 0.500 | 4691.119 | -2479.020 | 5010.809 |
| Model D | 0.704 | 591.070 | -2017.823 | 4107.852 | Model D | 0.533 | 4381.319 | -2462.839 | 4982.495 |
|  | | | | | | | | | |
| **Low back pain** | **R^2^** | **Sigma^2^** | **ML** | **AIC** | **Neck pain** | **R^2^** | **Sigma^2^** | **ML** | **AIC** |
|  |  |  |  |  |  |  |  |  |  |
| Model A | 0.413 | 14099.135 | -2731.378 | 5468.755 | Model A | 0.036 | 61.599 | -1533.354 | 3072.707 |
| Model B | 0.492 | 12368.528 | -2699.467 | 5416.934 | Model B | 0.110 | 57.657 | -1515.737 | 3049.473 |
| Model C | 0.593 | 9764.348 | -2642.193 | 5330.219 | Model C | 0.297 | 44.856 | -1456.033 | 2953.693 |
| Model D | 0.594 | 9726.650 | -2640.492 | 5330.055 | Model D | 0.298 | 44.772 | -1454.081 | 2956.582 |
|  | | | | | | | | | |
| **Gout** | **R^2^** | **Sigma^2^** | **ML** | **AIC** | **Other musculoskeletal disorders** | **R^2^** | **Sigma^2^** | **ML** | **AIC** |
|  |  |  |  |  |  |  |  |  |  |
| Model A | 0.155 | 215.992 | -1809.991 | 3625.981 | Model A | 0.055 | 31160.963 | -2906.246 | 5818.492 |
| Model B | 0.418 | 150.695 | -1727.579 | 3473.158 | Model B | 0.130 | 29099.445 | -2888.119 | 5794.238 |
| Model C | 0.644 | 90.868 | -1608.048 | 3275.234 | Model C | 0.295 | 23183.669 | -2833.528 | 5709.382 |
| Model D | 0.660 | 86.626 | -1597.797 | 3251.084 | Model D | 0.302 | 22975.203 | -2830.706 | 5706.808 |

**Model A:** DALYs ~ SDI.

**Model B:** DALYs ~ SDI + Meteorological factors.

**Model C:** DALYs ~ SDI + s (Meteorological factors).

**Model D:** DALYs ~ SDI + s (Meteorological Factors) + s (Years) + Obesity Prevalence + Smoking Rate.

**Supplementary Material Table 8. Comparisons of GLM and GAM model fit for males**

| **Rheumatoid arthritis** | **R^2^** | **Sigma^2^** | **ML** | **AIC** | **Osteoarthritis** | **R^2^** | **Sigma^2^** | **ML** | **AIC** |
| --- | --- | --- | --- | --- | --- | --- | --- | --- | --- |
|  |  |  |  |  |  |  |  |  |  |
| Model A | 0.169 | 391.477 | -1941.118 | 3888.237 | Model A | 0.071 | 5746.561 | -2533.476 | 5072.953 |
| Model B | 0.550 | 215.118 | -1806.062 | 3630.124 | Model B | 0.125 | 5488.131 | -2520.296 | 5058.592 |
| Model C | 0.662 | 159.102 | -1730.291 | 3524.661 | Model C | 0.609 | 2411.157 | -2328.465 | 4725.196 |
| Model D | 0.667 | 156.584 | -1725.614 | 3519.858 | Model D | 0.662 | 159.102 | -1730.291 | 3524.661 |
|  | | | | | | | | | |
| **Low back pain** | **R^2^** | **Sigma^2^** | **ML** | **AIC** | **Neck pain** | **R^2^** | **Sigma^2^** | **ML** | **AIC** |
|  |  |  |  |  |  |  |  |  |  |
| Model A | 0.264 | 17292.929 | -2776.400 | 5558.801 | Model A | 0.052 | 40.570 | -1441.269 | 2888.537 |
| Model B | 0.398 | 14349.474 | -2732.224 | 5482.448 | Model B | 0.115 | 38.403 | -1426.127 | 2870.253 |
| Model C | 0.533 | 10952.836 | -2666.830 | 5382.151 | Model C | 0.286 | 30.499 | -1366.706 | 2717.788 |
| Model D | 0.548 | 10602.484 | -2657.992 | 5371.649 | Model D | 0.390 | 26.074 | -1332.547 | 2681.538 |
|  | | | | | | | | | |
| **Gout** | **R^2^** | **Sigma^2^** | **ML** | **AIC** | **Other musculoskeletal disorders** | **R^2^** | **Sigma^2^** | **ML** | **AIC** |
|  |  |  |  |  |  |  |  |  |  |
| Model A | 0.017 | 1719.433 | -2267.419 | 4540.839 | Model A | 0.100 | 23873.199 | -2847.502 | 5701.005 |
| Model B | 0.253 | 1325.382 | -2206.990 | 4431.980 | Model B | 0.229 | 20733.740 | -2813.379 | 5644.758 |
| Model C | 0.461 | 940.094 | -2123.358 | 4305.703 | Model C | 0.419 | 15381.785 | -2740.915 | 5535.431 |
| Model D | 0.485 | 898.462 | -2112.047 | 4288.212 | Model D | 0.417 | 15420.062 | -2741.174 | 5536.331 |

**Model A:** DALYs ~ SDI.

**Model B:** DALYs ~ SDI + Meteorological factors.

**Model C:** DALYs ~ SDI + s (Meteorological factors).

**Model D:** DALYs ~ SDI + s (Meteorological Factors) + s (Years) + Obesity Prevalence + Smoking Rate.

**Supplementary Material Table 9. GLM and GAM estimates for females**

| **A: Parametric Coefficients (*p<0.05; **p<0.01; ***p<0.001)** | | | | |  |  |  |  |  | |  |
| --- | --- | --- | --- | --- | --- | --- | --- | --- | --- | --- | --- |
| **Rheumatoid arthritis** | **Model B** | | **Model D** | | **Osteoarthritis** | | **Model B** | | **Model D** | | |
|  | **Estimate** | **Std. Error** | **Estimate** | **Std. Error** |  |  | **Estimate** | **Std. Error** | **Estimate** | **Std. Error** | |
| Intercept | 2834.325 *** | 419.589 | 241.733 *** | 1.572 | Intercept | | -3806.661 ** | 1343.632 | 1559.405 *** | 4.230 | |
| SDI | -19.869 *** | 1.490 | -21.992 *** | 2.733 | SDI | | 22.397 *** | 4.772 | 33.732 *** | 7.203 | |
| Obesity Prevalence | - | - | -8.007 *** | 2.183 | Obesity Prevalence | | - | - | 25.534 *** | 5.668 | |
| Smoking Rate | - | - | 3.203 | 2.360 | Smoking Rate | | - | - | 21.817 ** | 6.265 | |
|  | | | | | | | | | | | |
| **Low back pain** | **Model B** | | **Model D** | | **Neck pain** | | **Model B** | | **Model D** | | |
|  | **Estimate** | **Std. Error** | **Estimate** | **Std. Error** |  |  | **Estimate** | **Std. Error** | **Estimate** | **Std. Error** | |
| Intercept | 7147.630 *** | 1645.754 | 2578.963 *** | 6.323 | Intercept | | 476.744 *** | 112.366 | 2578.963 *** | 6.323 | |
| SDI | -100.181 *** | 5.845 | -90.263 *** | 10.037 | SDI | | 1.438 *** | 0.399 | -90.263 *** | 10.037 | |
| Obesity Prevalence | - | - | -2.326 | 8.799 | Obesity Prevalence | | - | - | -2.326 | 8.799 | |
| Smoking Rate | - | - | 19.402 * | 9.404 | Smoking Rate | | - | - | 19.402 * | 9.404 | |
|  | | | | | | | | | | | |
| **Gout** | **Model B** | | **Model D** | | **Other musculoskeletal disorders** | | **Model B** | | **Model D** | | |
|  | **Estimate** | **Std. Error** | **Estimate** | **Std. Error** |  |  | **Estimate** | **Std. Error** | **Estimate** | **Std. Error** | |
| Intercept | -632.280 ** | 181.658 | 372.123 *** | 0.419 | Intercept | | 6689.110 ** | 2524.343 | 1943.846 *** | 9.512 | |
| SDI | -5.211 *** | 0.645 | 1.988 ** | 0.687 | SDI | | 38.720 *** | 8.965 | 1.408 | 16.198 | |
| Obesity Prevalence | - | - | 0.007 | 0.576 | Obesity Prevalence | | - | - | -10.971 | 13.014 | |
| Smoking Rate | - | - | 0.481 | 0.610 | Smoking Rate | | - | - | -34.075 * | 13.758 | |

| **B: Smooth Terms (Edf: Estimated Degrees of Freedom, *p<0.05; **p<0.01; ***p<0.001)** | | | | | | | |  |  |
| --- | --- | --- | --- | --- | --- | --- | --- | --- | --- |
| **Rheumatoid arthritis** | **Model B** | | **Model D** | | **Osteoarthritis** | **Model B** | | **Model D** | |
|  | **Estimate** | **Std. Error** | **Edf** | **Ref.df** |  | **Estimate** | **Std. Error** | **Edf** | **Ref.df** |
| TRAN_ANN | 11.179 *** | 2.093 | 4.917 *** | 9.000 | TRAN_ANN | -16.039 * | 6.702 | 5.202 *** | 9.000 |
| MEAN_TAVG | -5.592 ** | 1.846 | 1.994 ** | 9.000 | MEAN_TAVG | -10.184 | 5.913 | 4.288 ** | 9.000 |
| RRAV_ANN | -1.862 | 2.071 | 5.178 *** | 9.000 | RRAV_ANN | 0.362 | 6.633 | 0.002 | 9.000 |
| MEAN_RRAV | -17.490 | 13.204 | 2.455 ** | 9.000 | MEAN_RRAV | -31.869 | 42.282 | 0.825 * | 9.000 |
| MEAN_ASTP | -323.209 *** | 50.289 | 4.054 *** | 9.000 | MEAN_ASTP | 604.393 *** | 161.037 | 3.234 *** | 9.000 |
| MEAN_AWND | 66.846 *** | 11.562 | 4.906 *** | 9.000 | MEAN_AWND | 95.214 * | 37.023 | 3.364 *** | 9.000 |
| Year | - | - | 1.768 ** | 4.000 | Year | - | - | 2.119 *** | 4.000 |
|  | | | | | | | | | |
| **Low back pain** | **Model B** | | **Model D** | | **Neck pain** | **Model B** | | **Model D** | |
|  | **Estimate** | **Std. Error** | **Edf** | **Ref.df** |  | **Estimate** | **Std. Error** | **Edf** | **Ref.df** |
| TRAN_ANN | -17.897 * | 8.209 | 5.755 *** | 9.000 | TRAN_ANN | 1.908 ** | 0.560 | 1.436 * | 9.000 |
| MEAN_TAVG | -5.019 | 7.242 | 0.012 | 9.000 | MEAN_TAVG | -0.294 | 0.494 | 3.187 ** | 9.000 |
| RRAV_ANN | -13.087 | 8.124 | 0.740 | 9.000 | RRAV_ANN | 1.124 * | 0.555 | 0.002 | 9.000 |
| MEAN_RRAV | -227.266 | 51.789 | 2.639 *** | 9.000 | MEAN_RRAV | -1.493 | 3.536 | 1.539 ** | 9.000 |
| MEAN_ASTP | -469.174 * | 197.247 | 3.415 *** | 9.000 | MEAN_ASTP | -7.064 | 13.467 | 0.904 | 9.000 |
| MEAN_AWND | 104.562 * | 45.348 | 2.128 * | 9.000 | MEAN_AWND | -10.942 *** | 3.096 | 5.010 *** | 9.000 |
| Year | - | - | 0.919 *** | 4.000 | Year | - | - | 3.323 *** | 4.000 |
|  | | | | | | | | | |
| **Gout** | **Model B** | | **Model D** | | **Other musculoskeletal disorders** | **Model B** | | **Model D** | |
|  | **Estimate** | **Std. Error** | **Edf** | **Ref.df** |  | **Estimate** | **Std. Error** | **Edf** | **Ref.df** |
| TRAN_ANN | -5.943 *** | 0.906 | 0.991 *** | 9.000 | TRAN_ANN | -13.513 | 12.591 | 0.003 | 9.000 |
| MEAN_TAVG | -1.922 * | 0.799 | 0.001 | 9.000 | MEAN_TAVG | -21.000 | 11.108 | 1.072 * | 9.000 |
| RRAV_ANN | 2.069 * | 0.897 | 2.972 *** | 9.000 | RRAV_ANN | -7.516 | 12.461 | 0.161 | 9.000 |
| MEAN_RRAV | 11.974 * | 5.716 | 3.845 *** | 9.000 | MEAN_RRAV | -277.746 ** | 79.436 | 1.825 * | 9.000 |
| MEAN_ASTP | 83.907 *** | 21.772 | 5.581 *** | 9.000 | MEAN_ASTP | -420.738 | 302.548 | 5.025 *** | 9.000 |
| MEAN_AWND | -12.417 * | 5.006 | 3.097 *** | 9.000 | MEAN_AWND | 3.443 | 69.557 | 4.338 *** | 9.000 |
| Year | - | - | 2.376 *** | 4.000 | Year | - | - | 1.876 | 4.000 |

**Supplementary Material Table 10. GLM and GAM estimates for males**

| **A: Parametric Coefficients (*p<0.05; **p<0.01; ***p<0.001)** | | | | | | | | | |
| --- | --- | --- | --- | --- | --- | --- | --- | --- | --- |
| **Rheumatoid arthritis** | **Model B** | | **Model D** | | **Osteoarthritis** | **Model B** | | **Model D** | |
|  | **Estimate** | **Std. Error** | **Estimate** | **Std. Error** |  | **Estimate** | **Std. Error** | **Estimate** | **Std. Error** |
| Intercept | 1259.745 *** | 217.042 | 107.268 *** | 0.801 | Intercept | -1880.033 | 1096.272 | 1110.897 *** | 3.154 |
| SDI | -11.933 *** | 0.771 | -14.818 *** | 1.328 | SDI | 17.200 *** | 3.894 | 7.041 | 5.186 |
| Obesity Prevalence | - | - | 0.047 | 0.671 | Obesity Prevalence | - | - | 12.121 *** | 2.678 |
| Smoking Rate | - | - | -3.862 ** | 1.332 | Smoking Rate | - | - | 10.437 * | 5.220 |
|  | | | | | | | | | |
| **Low back pain** | **Model B** | | **Model D** | | **Neck pain** | **Model B** | | **Model D** | |
|  | **Estimate** | **Std. Error** | **Estimate** | **Std. Error** |  | **Estimate** | **Std. Error** | **Estimate** | **Std. Error** |
| Intercept | 9187.448 *** | 1772.654 | 2177.542 *** | 6.553 | Intercept | 302.684 ** | 91.704 | 314.191 *** | 0.309 |
| SDI | -85.935 *** | 6.296 | -72.709 *** | 10.403 | SDI | 1.314 *** | 0.326 | 1.206 ** | 0.455 |
| Obesity Prevalence | - | - | 21.376 *** | 5.363 | Obesity Prevalence | - | - | 0.299 | 0.293 |
| Smoking Rate | - | - | 13.092 | 10.727 | Smoking Rate | - | - | -0.324 | 0.485 |
|  | | | | | | | | | |
| **Gout** | **Model B** | | **Model D** | | **Other musculoskeletal disorders** | **Model B** | | **Model D** | |
|  | **Estimate** | **Std. Error** | **Estimate** | **Std. Error** |  | **Estimate** | **Std. Error** | **Estimate** | **Std. Error** |
| Intercept | 904.871 | 538.737 | 272.870 *** | 1.915 | Intercept | 2948.246 | 2130.810 | 1253.039 *** | 7.859 |
| SDI | 0.239 | 1.913 | 10.167 ** | 3.119 | SDI | 33.174 ** | 7.568 | 31.637 * | 12.713 |
| Obesity Prevalence | - | - | 0.360 | 1.614 | Obesity Prevalence | - | - | 8.425 | 6.504 |
| Smoking Rate | - | - | 14.353 *** | 3.154 | Smoking Rate | - | - | 19.633 | 12.803 |

| **B: Smooth Terms (Edf: Estimated Degrees of Freedom, *p<0.05; **p<0.01; ***p<0.001)** | | | | | | | | | |  |
| --- | --- | --- | --- | --- | --- | --- | --- | --- | --- | --- |
| **Rheumatoid arthritis** | **Model B** | | **Model D** | | **Osteoarthritis** | **Model B** | | **Model D** | |  |
|  | **Estimate** | **Std. Error** | **Edf** | **Ref.df** |  | **Estimate** | **Std. Error** | **Edf** | **Ref.df** |  |
| TRAN_ANN | 4.300 *** | 1.083 | 3.308 *** | 9.000 | TRAN_ANN | -19.227 *** | 5.468 | 5.385 *** | 9.000 |  |
| MEAN_TAVG | -3.577 *** | 0.955 | 3.936 *** | 9.000 | MEAN_TAVG | -15.436 ** | 4.824 | 3.391 *** | 9.000 |  |
| RRAV_ANN | -1.032 | 1.071 | 4.070 *** | 9.000 | RRAV_ANN | 2.559 | 5.412 | 0.001 | 9.000 |  |
| MEAN_RRAV | 7.238 | 6.830 | 2.880 *** | 9.000 | MEAN_RRAV | -70.917 * | 34.498 | 4.651 *** | 9.000 |  |
| MEAN_ASTP | -150.401 *** | 26.013 | 4.414 *** | 9.000 | MEAN_ASTP | 350.824 ** | 131.390 | 3.761 *** | 9.000 |  |
| MEAN_AWND | 27.978 *** | 5.981 | 4.534 *** | 9.000 | MEAN_AWND | 85.737 ** | 30.207 | 4.253 *** | 9.000 |  |
| Year | - | - | 0.839 * | 4.000 | Year | - | - | 2.645 *** | 4.000 |  |
|  | | | | | | | | | |  |
|  |  |  |  |  |  |  |  |  |  |  |
|  |  |  |  |  |  |  |  |  |  |  |
| **Low back pain** | **Model B** | | **Model D** | | **Neck pain** | **Model B** | | **Model D** | |  |
|  | **Estimate** | **Std. Error** | **Edf** | **Ref.df** |  | **Estimate** | **Std. Error** | **Edf** | **Ref.df** |  |
| TRAN_ANN | -9.090 | 8.842 | 5.631 *** | 9.000 | TRAN_ANN | 1.054 * | 0.457 | 1.606 ** | 9.000 |  |
| MEAN_TAVG | -15.588 * | 7.800 | 0.998 * | 9.000 | MEAN_TAVG | -0.743 | 0.404 | 3.270 ** | 9.000 |  |
| RRAV_ANN | -11.910 | 8.751 | 0.385 | 9.000 | RRAV_ANN | 1.137 * | 0.453 | 0.001 | 9.000 |  |
| MEAN_RRAV | -137.078 * | 55.782 | 2.806 *** | 9.000 | MEAN_RRAV | -1.101 | 2.886 | 1.266 * | 9.000 |  |
| MEAN_ASTP | -812.085 *** | 212.456 | 3.981 *** | 9.000 | MEAN_ASTP | 4.812 | 10.991 | 0.001 | 9.000 |  |
| MEAN_AWND | 140.262 ** | 48.845 | 2.795 *** | 9.000 | MEAN_AWND | -7.124 ** | 2.527 | 5.069 *** | 9.000 |  |
| Year | - | - | 1.891 *** | 4.000 | Year | - | - | 3.638 *** | 4.000 |  |
|  | | | | | | | | | |  |
|  |  |  |  |  |  |  |  |  |  |  |
| **Gout** | **Model B** | | **Model D** | | **Other musculoskeletal disorders** | **Model B** | | **Model D** | |  |
|  | **Estimate** | **Std. Error** | **Edf** | **Ref.df** |  | **Estimate** | **Std. Error** | **Edf** | **Ref.df** |  |
| TRAN_ANN | -22.081 *** | 2.687 | 3.111 *** | 9.000 | TRAN_ANN | 18.779 | 10.628 | 0.815 | 9.000 |  |
| MEAN_TAVG | -2.759 | 2.371 | 2.595 *** | 9.000 | MEAN_TAVG | -35.885 *** | 9.376 | 2.256 *** | 9.000 |  |
| RRAV_ANN | 9.359 *** | 2.659 | 2.402 *** | 9.000 | RRAV_ANN | -4.874 | 10.519 | 1.077 | 9.000 |  |
| MEAN_RRAV | 11.231 | 16.953 | 2.789 *** | 9.000 | MEAN_RRAV | -158.709 * | 67.053 | 2.035 ** | 9.000 |  |
| MEAN_ASTP | -71.192 | 64.569 | 3.523 *** | 9.000 | MEAN_ASTP | -135.760 | 255.382 | 4.722 *** | 9.000 |  |
| MEAN_AWND | -18.827 | 14.845 | 4.907 *** | 9.000 | MEAN_AWND | 37.305 | 58.714 | 4.397 *** | 9.000 |  |
| Year | - | - | 2.403 *** | 4.000 | Year | - | - | 2.070 *** | 4.000 |  |

**Supplementary Material Table 11. Sensitivity analysis for both sexes (A: Parametric Coefficients; B: Smooth Terms; *p<0.05, **p<0.01, ***p<0.001).**

| **A: Parametric Coefficients** | | | | | |  |  |  |  | |  | | | |  | |  |  |
| --- | --- | --- | --- | --- | --- | --- | --- | --- | --- | --- | --- | --- | --- | --- | --- | --- | --- | --- |
| **Sensitivity Model S1** | | **Rheumatoid arthritis** | | **Osteoarthritis** | | **Low back pain** | | **Neck pain** | | | | | **Gout** | | | **Other musculoskeletal disorders** | | |
|  |  | **Estimate** | **Std. Error** | **Estimate** | **Std. Error** | **Estimate** | **Std. Error** | **Estimate** | **Estimate** | | **Std. Error** | | | | **Estimate** | | **Std. Error** | **Std. Error** |
| Intercept | | 222.627 *** | 3.423 | 1286.534 *** | 11.695 | 2509.397 *** | 19.124 | 343.713 *** | 1.059 | | 170.737 *** | | | | 3.598 | | 1628.925 *** | 25.610 |
| Population Density | | -17.375 *** | 1.278 | 24.302 *** | 4.417 | -43.195 *** | 7.148 | 0.530 | 0.391 | | 2.115 | | | | 1.345 | | 2.650 | 9.539 |
| Obesity Prevalence | | -4.691 *** | 1.284 | 25.522 *** | 5.887 | 44.408 *** | 8.318 | 0.252 | 0.582 | | -2.924 | | | | 1.787 | | -6.543 | 13.376 |
| Smoking Rate | | 1.796 | 1.347 | 13.256 * | 5.167 | 28.034 ** | 8.010 | -0.733 | 0.556 | | 9.057 *** | | | | 1.666 | | -16.269 | 12.334 |
|  |  |  |  |  |  |  |  |  |  | |  | | | |  | |  |  |
| **Sensitivity Model S2** | | **Rheumatoid arthritis** | | **Osteoarthritis** | | **Low back pain** | | **Neck pain** | | **Gout** | | | | | | **Other musculoskeletal disorders** | | |
|  |  | **Estimate** | **Std. Error** | **Estimate** | **Std. Error** | **Estimate** | **Std. Error** | **Estimate** | **Std. Error** | | **Estimate** | | | | **Std. Error** | | **Estimate** | **Std. Error** |
| Intercept | | 179.361 *** | 0.977 | 2.912 *** | 462.912 | 2395.718 *** | 5.075 | 345.436 *** | 0.310 | | 174.947 *** | | | | 0.948 | | 1637.002 *** | 7.159 |
| Healthcare Acces PC1 | | -6.052 *** | 1.203 | 3.756 *** | 5.602 | -7.087 | 6.067 | 0.550 | 0.370 | | -1.278 | | | | 1.171 | | -2.680 | 8.615 |
| Healthcare Acces PC2 | | -12.518 *** | 1.823 | 5.730 ** | 2.812 | 10.267 | 9.692 | -1.653 ** | 0.580 | | 6.420 ** | | | | 1.835 | | -10.518 | 13.444 |
| Obesity Prevalence | | 5.434 *** | 1.358 | 5.333 * | 2.440 | 63.773 *** | 8.052 | 0.491 | 0.556 | | -5.565 ** | | | | 1.739 | | -8.731 | 12.482 |
| Smoking Rate | | 9.663 *** | 1.408 | 4.997 | 0.980 | 39.897 *** | 8.049 | -0.790 | 0.522 | | 7.555 *** | | | | 1.613 | | -15.805 | 11.869 |
|  |  |  |  |  |  |  |  |  |  | |  | | | |  | |  |  |
|  |  |  |  |  |  |  |  |  |  | |  | | | |  | |  |  |
| **B: Smooth Terms** | | | | | |  |  |  |  | |  | | | |  | |  |  |
| **Sensitivity Model S1** | | **Rheumatoid arthritis** | | **Osteoarthritis** | | **Low back pain** | | **Neck pain** | | | | | | **Gout** | | **Other musculoskeletal disorders** | | |
|  |  | **Edf** | **Ref.df** | **Edf** | **Ref.df** | **Edf** | **Ref.df** | **Edf** | **Ref.df** | | **Edf** | | | | **Ref.df** | | **Edf** | **Ref.df** |
| TRAN_ANN | | 4.895 *** | 9.000 | 4.658 *** | 9.000 | 6.486 *** | 9.000 | 1.524 * | 9.000 | | | 3.472 *** | | | 9.000 | | 0.001 | 9.000 |
| MEAN_TAVG | | 4.183 ** | 9.000 | 2.956 *** | 9.000 | 0.409 | 9.000 | 3.483 *** | 9.000 | | | 2.597 *** | | | 9.000 | | 1.755 *** | 9.000 |
| RRAV_ANN | | 4.016 ** | 9.000 | 0.002 | 9.000 | 0.000 | 9.000 | 0.001 | 9.000 | | | 2.555 *** | | | 9.000 | | 0.391 | 9.000 |
| MEAN_RRAV | | 2.992 *** | 9.000 | 4.936 *** | 9.000 | 3.001 *** | 9.000 | 1.360 ** | 9.000 | | | 2.874 ** | | | 9.000 | | 1.909 * | 9.000 |
| MEAN_ASTP | | 3.137 *** | 9.000 | 3.937 *** | 9.000 | 3.989 *** | 9.000 | 1.908 | 9.000 | | | 3.874 *** | | | 9.000 | | 4.763 *** | 9.000 |
| MEAN_AWND | | 3.552 *** | 9.000 | 3.788 *** | 9.000 | 2.077 ** | 9.000 | 4.849 *** | 9.000 | | | 4.276 *** | | | 9.000 | | 4.373 *** | 9.000 |
| Year | | 0.001 | 4.000 | 2.387 *** | 4.000 | 0.967 | 4.000 | 3.477 *** | 4.000 | | | 2.528 *** | | | 4.000 | | 1.930 *** | 4.000 |
|  |  |  |  |  |  |  |  |  |  | |  | | | |  | |  |  |
| **Sensitivity Model S2** | | **Rheumatoid arthritis** | | **Osteoarthritis** | | **Low back pain** | | **Neck pain** | | | | | | **Gout** | | **Other musculoskeletal disorders** | | |
|  |  | **Edf** | **Ref.df** | **Edf** | **Ref.df** | **Edf** | **Ref.df** | **Edf** | **Ref.df** | | **Edf** | | | | **Ref.df** | | **Edf** | **Ref.df** |
| TRAN_ANN | | 2.456 * | 9.000 | 5.703 *** | 9.000 | 5.993 *** | 9.000 | 1.520 * | 9.000 | | | 4.304 *** | | | 9.000 | | 0.002 | 9.000 |
| MEAN_TAVG | | 3.294 ** | 9.000 | 2.865 ** | 9.000 | 0.000 | 9.000 | 3.720 *** | 9.000 | | | 1.995 * | | | 9.000 | | 1.604 *** | 9.000 |
| RRAV_ANN | | 2.882 * | 9.000 | 0.003 | 9.000 | 0.022 | 9.000 | 0.500 | 9.000 | | | 2.118 *** | | | 9.000 | | 0.472 | 9.000 |
| MEAN_RRAV | | 0.001 | 9.000 | 4.251 *** | 9.000 | 2.898 *** | 9.000 | 1.364 * | 9.000 | | | 2.468 ** | | | 9.000 | | 1.684 *** | 9.000 |
| MEAN_ASTP | | 3.769 *** | 9.000 | 4.338 *** | 9.000 | 4.347 *** | 9.000 | 2.405 * | 9.000 | | | 3.499 *** | | | 9.000 | | 4.591 | 9.000 |
| MEAN_AWND | | 4.252 *** | 9.000 | 3.395 *** | 9.000 | 2.061 ** | 9.000 | 5.203 *** | 9.000 | | | 3.772 *** | | | 9.000 | | 4.338 *** | 9.000 |
| Year | | 0.001 | 4.000 | 2.300 *** | 4.000 | 0.935 | 4.000 | 3.144 *** | 4.000 | | | 2.825 *** | | | 4.000 | | 1.947 *** | 4.000 |

**Sensitivity Model S1:** DALYs ~ Population Density (55+) + s(Meteorological factors) + s(Year) + Obesity Prevalence + Smoking Rate.

Adjusted R^2^: Rheumatoid arthritis 0.732; Osteoarthritis 0.589; Low back pain 0.598; Neck pain 0.313; Gout 0.521; Other musculoskeletal disorders 0.320.

**Sensitivity Model S2:** DALYs ~ Healthaccess_ PC1 + Healthaccess_ PC2 + s(Meteorological factors) + s(Year) + Obesity Prevalence + Smoking Rate

Adjusted R^2^: Rheumatoid arthritis 0.653; Osteoarthritis 0.588; Low back pain 0.569; Neck pain 0.333; Gout 0.528; Other musculoskeletal disorders 0.317.

**Estimate:** Linear coefficient.

**Std. Error:** Standard Error.

**Edf:** Estimated Degrees of Freedom.

**Ref.df:** Reference Degrees of Freedom

**Supplementary Material Table 12. Sensitivity analysis for females (A: Parametric Coefficients; B: Smooth Terms; *p<0.05, **p<0.01, ***p<0.001).**

| **A: Parametric Coefficients** | | | | | |  |  |  |  |  | | | |  | |  |  |
| --- | --- | --- | --- | --- | --- | --- | --- | --- | --- | --- | --- | --- | --- | --- | --- | --- | --- |
| **Sensitivity Model S1** | | **Rheumatoid arthritis** | | **Osteoarthritis** | | **Low back pain** | | **Neck pain** | | | | **Gout** | | | **Other musculoskeletal disorders** | | |
|  |  | **Estimate** | **Std. Error** | **Estimate** | **Std. Error** | **Estimate** | **Std. Error** | **Estimate** | **Estimate** | **Std. Error** | | | | **Estimate** | | **Std. Error** | **Std. Error** |
| Intercept | | 297.968 *** | 3.986 | 1480.676 *** | 11.543 | 2725.748 *** | 17.235 | 371.546 *** | 1.092 | 83.493 *** | | | | 1.617 | | 1940.503 *** | 25.251 |
| Population Density | | -21.346 *** | 1.597 | 29.379 *** | 4.570 | -52.406 *** | 6.867 | 0.083 | 0.432 | 3.300 *** | | | | 0.640 | | 1.260 | 9.982 |
| Obesity Prevalence | | -6.416 *** | 1.444 | 22.329 *** | 5.324 | 19.255 ** | 6.318 | -0.636 | 0.534 | 4.238 *** | | | | 0.721 | | -10.996 ** | 11.837 |
| Smoking Rate | | 3.522 * | 1.772 | 18.579 ** | 5.574 | 37.081 *** | 7.945 | -0.225 | 0.573 | 5.831 *** | | | | 0.788 | | -34.154 *** | 12.598 |
|  |  |  |  |  |  |  |  |  |  |  | | | |  | |  |  |
| **Sensitivity Model S2** | | **Rheumatoid arthritis** | | **Osteoarthritis** | | **Low back pain** | | **Neck pain** | | | | **Gout** | | | **Other musculoskeletal disorders** | | |
|  |  | **Estimate** | **Std. Error** | **Estimate** | **Std. Error** | **Estimate** | **Std. Error** | **Estimate** | **Std. Error** | **Estimate** | | | | **Std. Error** | | **Estimate** | **Std. Error** |
| Intercept | | 263.673 *** | 3.315 | 1525.277 *** | 8.812 | 2583.055 *** | 11.849 | 374.635 *** | 0.854 | 90.783 *** | | | | 1.244 | | 1943.151 *** | 19.697 |
| Healthcare Acces PC1 | | -8.522 *** | 1.766 | 21.292 *** | 4.635 | -3.025 | 5.767 | -0.744 | 0.446 | -0.559 | | | | 0.632 | | 2.773 | 10.157 |
| Healthcare Acces PC2 | | -12.288 *** | 2.429 | 9.242 | 6.433 | 27.278 ** | 9.685 | -3.000 *** | 0.638 | 1.587 | | | | 0.918 | | -2.826 | 14.448 |
| Obesity Prevalence | | 1.708 | 2.032 | 13.547 * | 5.258 | 27.860 *** | 7.219 | -0.007 | 0.521 | 2.687 *** | | | | 0.726 | | -10.986 | 11.705 |
| Smoking Rate | | 15.754 *** | 1.997 | 2.416 | 5.294 | 59.409 *** | 7.531 | 0.129 | 0.517 | 4.335 *** | | | | 0.754 | | -34.555 ** | 11.718 |
|  |  |  |  |  |  |  |  |  |  |  | | | |  | |  |  |
|  |  |  |  |  |  |  |  |  |  |  | | | |  | |  |  |
| **B: Smooth Terms** | | | | | |  |  |  |  |  | | | |  | |  |  |
| **Sensitivity Model S1** | | **Rheumatoid arthritis** | | **Osteoarthritis** | | **Low back pain** | | **Neck pain** | | | | | **Gout** | | **Other musculoskeletal disorders** | | |
|  |  | **Edf** | **Ref.df** | **Edf** | **Ref.df** | **Edf** | **Ref.df** | **Edf** | **Ref.df** | **Edf** | | | | **Ref.df** | | **Edf** | **Ref.df** |
| TRAN_ANN | | 5.049 *** | 9.000 | 4.350 *** | 9.000 | 6.060 *** | 9.000 | 1.353 * | 9.000 | | 0.899 ** | | | 9.000 | | 0.001 | 9.000 |
| MEAN_TAVG | | 3.903 ** | 9.000 | 3.442 *** | 9.000 | 1.597 ** | 9.000 | 3.581 *** | 9.000 | | 3.212 ** | | | 9.000 | | 1.113 * | 9.000 |
| RRAV_ANN | | 5.548 *** | 9.000 | 0.000 | 9.000 | 0.079 | 9.000 | 0.001 | 9.000 | | 2.944 *** | | | 9.000 | | 0.128 | 9.000 |
| MEAN_RRAV | | 2.961 *** | 9.000 | 0.791 * | 9.000 | 2.967 *** | 9.000 | 1.654 ** | 9.000 | | 3.575 *** | | | 9.000 | | 1.835 * | 9.000 |
| MEAN_ASTP | | 3.235 *** | 9.000 | 4.015 *** | 9.000 | 3.230 *** | 9.000 | 2.180 *** | 9.000 | | 4.779 *** | | | 9.000 | | 5.031 *** | 9.000 |
| MEAN_AWND | | 3.622 *** | 9.000 | 3.600 *** | 9.000 | 1.928 ** | 9.000 | 4.863 *** | 9.000 | | 3.237 *** | | | 9.000 | | 4.344 *** | 9.000 |
| Year | | 0.001 | 4.000 | 2.296 *** | 4.000 | 0.000 | 4.000 | 3.206 | 4.000 | | 2.334 *** | | | 4.000 | | 1.895 *** | 4.000 |
|  |  |  |  |  |  |  |  |  |  |  | | | |  | |  |  |
| **Sensitivity Model S2** | | **Rheumatoid arthritis** | | **Osteoarthritis** | | **Low back pain** | | **Neck pain** | | | | | **Gout** | | **Other musculoskeletal disorders** | | |
|  |  | **Edf** | **Ref.df** | **Edf** | **Ref.df** | **Edf** | **Ref.df** | **Edf** | **Ref.df** | **Edf** | | | | **Ref.df** | | **Edf** | **Ref.df** |
| TRAN_ANN | | 3.488 *** | 9.000 | 4.941 *** | 9.000 | 5.331 *** | 9.000 | 1.429 * | 9.000 | | 0.978 *** | | | 9.000 | | 0.002 | 9.000 |
| MEAN_TAVG | | 2.899 ** | 9.000 | 4.384 ** | 9.000 | 0.133 | 9.000 | 3.876 *** | 9.000 | | 0.001 | | | 9.000 | | 0.937 * | 9.000 |
| RRAV_ANN | | 4.493 ** | 9.000 | 0.001 | 9.000 | 0.314 | 9.000 | 0.315 | 9.000 | | 2.475 *** | | | 9.000 | | 0.086 | 9.000 |
| MEAN_RRAV | | 0.003 | 9.000 | 0.572 | 9.000 | 3.317 *** | 9.000 | 1.289 * | 9.000 | | 3.632 *** | | | 9.000 | | 1.752 * | 9.000 |
| MEAN_ASTP | | 3.842 *** | 9.000 | 4.340 *** | 9.000 | 3.846 *** | 9.000 | 2.972 ** | 9.000 | | 5.407 *** | | | 9.000 | | 5.030 *** | 9.000 |
| MEAN_AWND | | 4.486 *** | 9.000 | 3.379 *** | 9.000 | 1.617 * | 9.000 | 5.261 *** | 9.000 | | 3.069 *** | | | 9.000 | | 4.319 *** | 9.000 |
| Year | | 1.668 * | 4.000 | 2.002 *** | 4.000 | 0.001 | 4.000 | 2.900 | 4.000 | | 2.329 *** | | | 4.000 | | 1.835 ** | 4.000 |

**Sensitivity Model S1:** DALYs ~ Population Density (55+) + s(Meteorological factors) + s(Year) + Obesity Prevalence + Smoking Rate.

Adjusted R^2^: Rheumatoid arthritis 0.756; Osteoarthritis 0.547; Low back pain 0.583; Neck pain 0.394; Gout 0.677; Other musculoskeletal disorders 0.302.

**Sensitivity Model S2:** DALYs ~ Healthaccess_ PC1 + Healthaccess_ PC2 + s(Meteorological factors) + s(Year) + Obesity Prevalence + Smoking Rate

Adjusted R^2^: Rheumatoid arthritis 0.674; Osteoarthritis 0.533; Low back pain 0.541; Neck pain 0.331; Gout 0.661; Other musculoskeletal disorders 0.300.

**Estimate:** Linear coefficient.

**Std. Error:** Standard Error.

**Edf:** Estimated Degrees of Freedom.

**Ref.df:** Reference Degrees of Freedom

**Supplementary Material Table 13. Sensitivity analysis for males (A: Parametric Coefficients; B: Smooth Terms; *p<0.05, **p<0.01, ***p<0.001).**

| **A: Parametric Coefficients** | | | | | |  |  |  |  |  | | | |  | |  |  |
| --- | --- | --- | --- | --- | --- | --- | --- | --- | --- | --- | --- | --- | --- | --- | --- | --- | --- |
| **Sensitivity Model S1** | | **Rheumatoid arthritis** | | **Osteoarthritis** | | **Low back pain** | | **Neck pain** | | | | **Gout** | | | **Other musculoskeletal disorders** | | |
|  |  | **Estimate** | **Std. Error** | **Estimate** | **Std. Error** | **Estimate** | **Std. Error** | **Estimate** | **Estimate** | **Std. Error** | | | | **Estimate** | | **Std. Error** | **Std. Error** |
| Intercept | | 138.694 *** | 2.390 | 1103.416 *** | 10.942 | 2333.632 *** | 20.832 | 313.632 *** | 0.950 | 256.620 *** | | | | 6.537 | | 1262.310 *** | 26.014 |
| Population Density | | -12.411 *** | 0.801 | 3.253 | 3.662 | -61.022 *** | 6.962 | 0.302 | 0.313 | 6.660 ** | | | | 2.180 | | -0.207 | 8.684 |
| Obesity Prevalence | | -0.024 | 0.597 | 11.591 *** | 2.654 | 18.235 *** | 5.037 | 0.157 | 0.291 | 0.145 | | | | 1.607 | | 2.436 | 6.405 |
| Smoking Rate | | -0.905 | 0.959 | 7.978 | 4.500 | 24.941 ** | 8.563 | -0.854 | 0.446 | 11.306 *** | | | | 2.696 | | 0.037 | 10.974 |
|  |  |  |  |  |  |  |  |  |  |  | | | |  | |  |  |
| **Sensitivity Model S2** | | **Rheumatoid arthritis** | | **Osteoarthritis** | | **Low back pain** | | **Neck pain** | | | | **Gout** | | | **Other musculoskeletal disorders** | | |
|  |  | **Estimate** | **Std. Error** | **Estimate** | **Std. Error** | **Estimate** | **Std. Error** | **Estimate** | **Estimate** | **Std. Error** | | | | **Estimate** | | **Std. Error** | **Std. Error** |
| Intercept | | 95.925 *** | 1.592 | 1140.931 *** | 5.549 | 2180.917 *** | 12.251 | 314.286 *** | 0.533 | 288.354 *** | | | | 3.368 | | 1253.016 *** | 14.280 |
| Healthcare Acces PC1 | | -1.919 * | 0.913 | 17.351 *** | 3.016 | -0.455 | 6.519 | 0.854 ** | 0.293 | 0.037 | | | | 1.844 | | -2.066 | 7.688 |
| Healthcare Acces PC2 | | -6.440 *** | 1.304 | 16.869 *** | 4.561 | 26.722 ** | 9.686 | -0.980 * | 0.464 | 13.786 *** | | | | 2.777 | | -8.037 | 11.732 |
| Obesity Prevalence | | 2.493 *** | 0.710 | 9.260 *** | 2.408 | 31.358 *** | 5.296 | 0.211 | 0.278 | -1.498 | | | | 1.503 | | 2.551 | 6.160 |
| Smoking Rate | | 6.468 *** | 1.175 | 5.925 | 3.996 | 56.030 *** | 8.755 | -0.895 * | 0.389 | 5.004 * | | | | 2.439 | | 1.079 | 10.267 |
|  |  |  |  |  |  |  |  |  |  |  | | | |  | |  |  |
|  |  |  |  |  |  |  |  |  |  |  | | | |  | |  |  |
| **B: Smooth Terms** | | | | | |  |  |  |  |  | | | |  | |  |  |
| **Sensitivity Model S1** | | **Rheumatoid arthritis** | | **Osteoarthritis** | | **Low back pain** | | **Neck pain** | | | | | **Gout** | | **Other musculoskeletal disorders** | | |
|  |  | **Edf** | **Ref.df** | **Edf** | **Ref.df** | **Edf** | **Ref.df** | **Edf** | **Ref.df** | **Edf** | | | | **Ref.df** | | **Edf** | **Ref.df** |
| TRAN_ANN | | 2.044 | 9.000 | 5.313 *** | 9.000 | 6.015 *** | 9.000 | 1.580 ** | 9.000 | | 3.090 ** | | | 9.000 | | 3.274 * | 9.000 |
| MEAN_TAVG | | 4.290 *** | 9.000 | 3.406 *** | 9.000 | 0.206 | 9.000 | 3.469 *** | 9.000 | | 3.184 *** | | | 9.000 | | 2.423 *** | 9.000 |
| RRAV_ANN | | 4.855 *** | 9.000 | 0.001 | 9.000 | 0.001 | 9.000 | 0.001 | 9.000 | | 2.432 *** | | | 9.000 | | 0.996 | 9.000 |
| MEAN_RRAV | | 3.170 *** | 9.000 | 4.935 *** | 9.000 | 3.630 *** | 9.000 | 1.307 * | 9.000 | | 2.710 *** | | | 9.000 | | 1.299 ** | 9.000 |
| MEAN_ASTP | | 3.450 *** | 9.000 | 3.698 *** | 9.000 | 4.102 *** | 9.000 | 1.498 | 9.000 | | 3.495 *** | | | 9.000 | | 4.640 *** | 9.000 |
| MEAN_AWND | | 3.055 *** | 9.000 | 4.342 *** | 9.000 | 2.475 *** | 9.000 | 4.947 *** | 9.000 | | 5.168 *** | | | 9.000 | | 4.421 *** | 9.000 |
| Year | | 0.002 | 4.000 | 2.646 *** | 4.000 | 0.815 * | 4.000 | 3.616 *** | 4.000 | | 2.423 *** | | | 4.000 | | 2.000 *** | 4.000 |
|  |  |  |  |  |  |  |  |  |  |  | | | |  | |  |  |
| **Sensitivity Model S2** | | **Rheumatoid arthritis** | | **Osteoarthritis** | | **Low back pain** | | **Neck pain** | | | | | **Gout** | | **Other musculoskeletal disorders** | | |
|  |  | **Edf** | **Ref.df** | **Edf** | **Ref.df** | **Edf** | **Ref.df** | **Edf** | **Ref.df** | **Edf** | | | | **Ref.df** | | **Edf** | **Ref.df** |
| TRAN_ANN | | 0.900 ** | 9.000 | 5.661 *** | 9.000 | 5.059 *** | 9.000 | 1.543 * | 9.000 | | 3.388 *** | | | 9.000 | | 3.409 * | 9.000 |
| MEAN_TAVG | | 4.316 *** | 9.000 | 1.894 ** | 9.000 | 0.001 | 9.000 | 3.453 ** | 9.000 | | 2.326 * | | | 9.000 | | 2.392 *** | 9.000 |
| RRAV_ANN | | 3.765 ** | 9.000 | 0.002 | 9.000 | 0.029 | 9.000 | 0.001 | 9.000 | | 1.562 *** | | | 9.000 | | 0.984 | 9.000 |
| MEAN_RRAV | | 0.928 *** | 9.000 | 3.452 *** | 9.000 | 2.774 *** | 9.000 | 1.343 * | 9.000 | | 2.689 *** | | | 9.000 | | 1.343 ** | 9.000 |
| MEAN_ASTP | | 4.335 *** | 9.000 | 4.620 *** | 9.000 | 4.958 *** | 9.000 | 0.000 | 9.000 | | 3.430 *** | | | 9.000 | | 4.537 *** | 9.000 |
| MEAN_AWND | | 4.626 *** | 9.000 | 4.199 *** | 9.000 | 2.654 ** | 9.000 | 5.088 *** | 9.000 | | 4.705 *** | | | 9.000 | | 4.440 *** | 9.000 |
| Year | | 1.867 *** | 4.000 | 2.493 *** | 4.000 | 0.916 ** | 4.000 | 3.455 *** | 4.000 | | 2.420 *** | | | 4.000 | | 2.019 *** | 4.000 |

**Sensitivity Model S1:** DALYs ~ Population Density (55+) + s(Meteorological factors) + s(Year) + Obesity Prevalence + Smoking Rate.

Adjusted R^2^: Rheumatoid arthritis 0.702; Osteoarthritis 0.601; Low back pain 0.568; Neck pain 0.388; Gout 0.485; Other musculoskeletal disorders 0.416.

**Sensitivity Model S2:** DALYs ~ Healthaccess_ PC1 + Healthaccess_ PC2 + s(Meteorological factors) + s(Year) + Obesity Prevalence + Smoking Rate

Adjusted R^2^: Rheumatoid arthritis 0.587; Osteoarthritis 0.633; Low back pain 0.507; Neck pain 0.394; Gout 0.496; Other musculoskeletal disorders 0.416.

**Estimate:** Linear coefficient.

**Std. Error:** Standard Error.

**Edf:** Estimated Degrees of Freedom.

**Ref.df:** Reference Degrees of Freedom

**
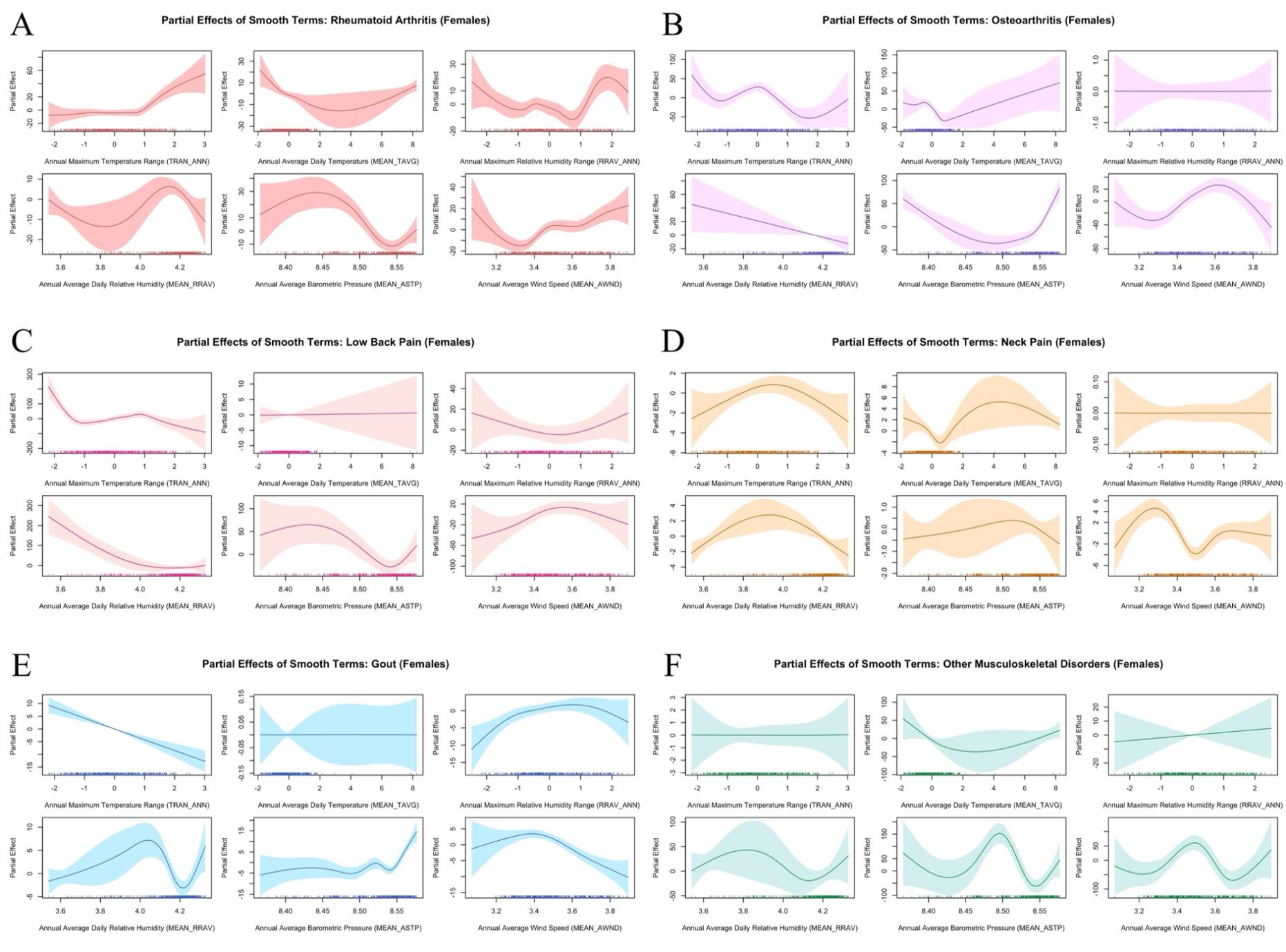
**

**Supplementary Material Figure 6. Partial effects of GAM smooth terms (Model D) for females**

**(A: rheumatoid arthritis; B: osteoarthritis; C: low back pain; D: neck pain; E: gout; F: other musculoskeletal disorders).**

TRAN_ANN: Annual maximum temperature range; MEAN_TAVG: Annual average daily temperature; RRAV_ANN: Annual relative humidity range; MEAN_RRAV: Annual average daily relative humidity; MEAN_ASTP: Annual average barometric pressure; MEAN_AWND: Annual average wind speed.

**
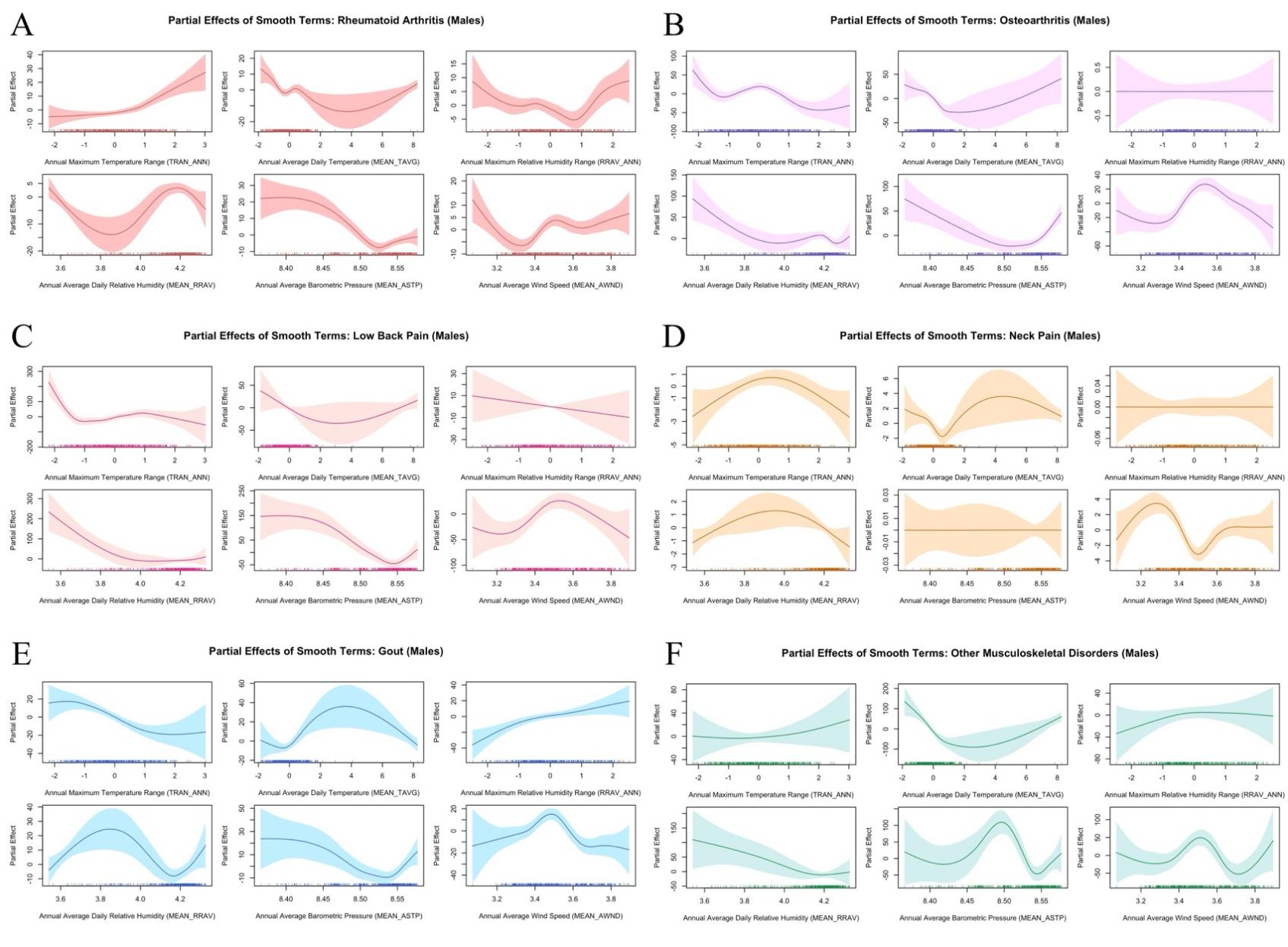
**

**Supplementary Material Figure 7.** **Partial effects of GAM smooth terms** **(Model D) for males**

**(A: rheumatoid arthritis; B: osteoarthritis; C: low back pain; D: neck pain; E: gout; F: other musculoskeletal disorders).**

TRAN_ANN: Annual maximum temperature range; MEAN_TAVG: Annual average daily temperature; RRAV_ANN: Annual relative humidity range; MEAN_RRAV: Annual average daily relative humidity; MEAN_ASTP: Annual average barometric pressure; MEAN_AWND: Annual average wind speed


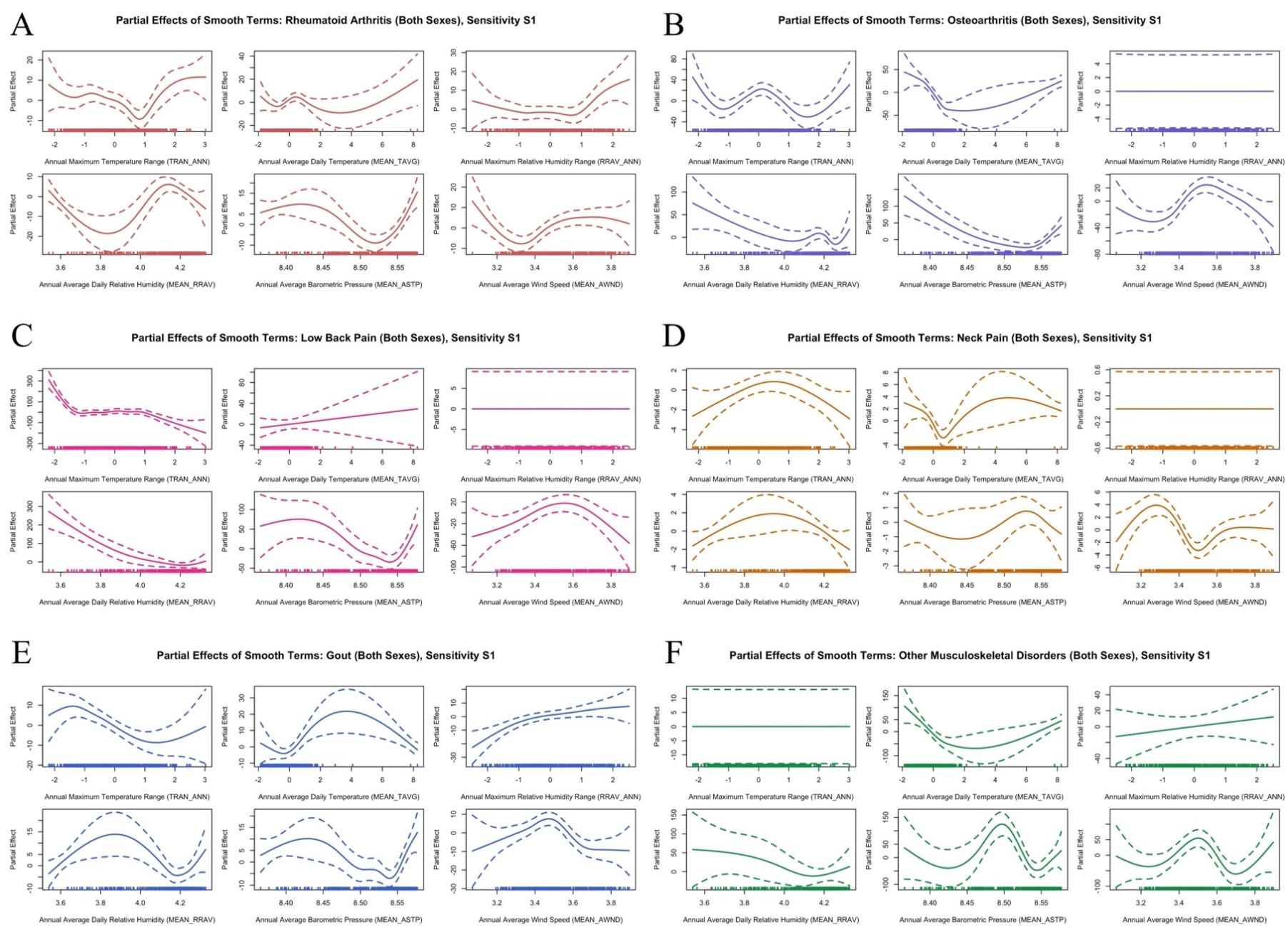


**Supplementary Material Figure 8.** **Partial effects of GAM smooth terms (****Sensitivity Model S1) for both sexes**

**(A: rheumatoid arthritis; B: osteoarthritis; C: low back pain; D: neck pain; E: gout; F: other musculoskeletal disorders).**

TRAN_ANN: Annual maximum temperature range; MEAN_TAVG: Annual average daily temperature; RRAV_ANN: Annual relative humidity range; MEAN_RRAV: Annual average daily relative humidity; MEAN_ASTP: Annual average barometric pressure; MEAN_AWND: Annual average wind speed


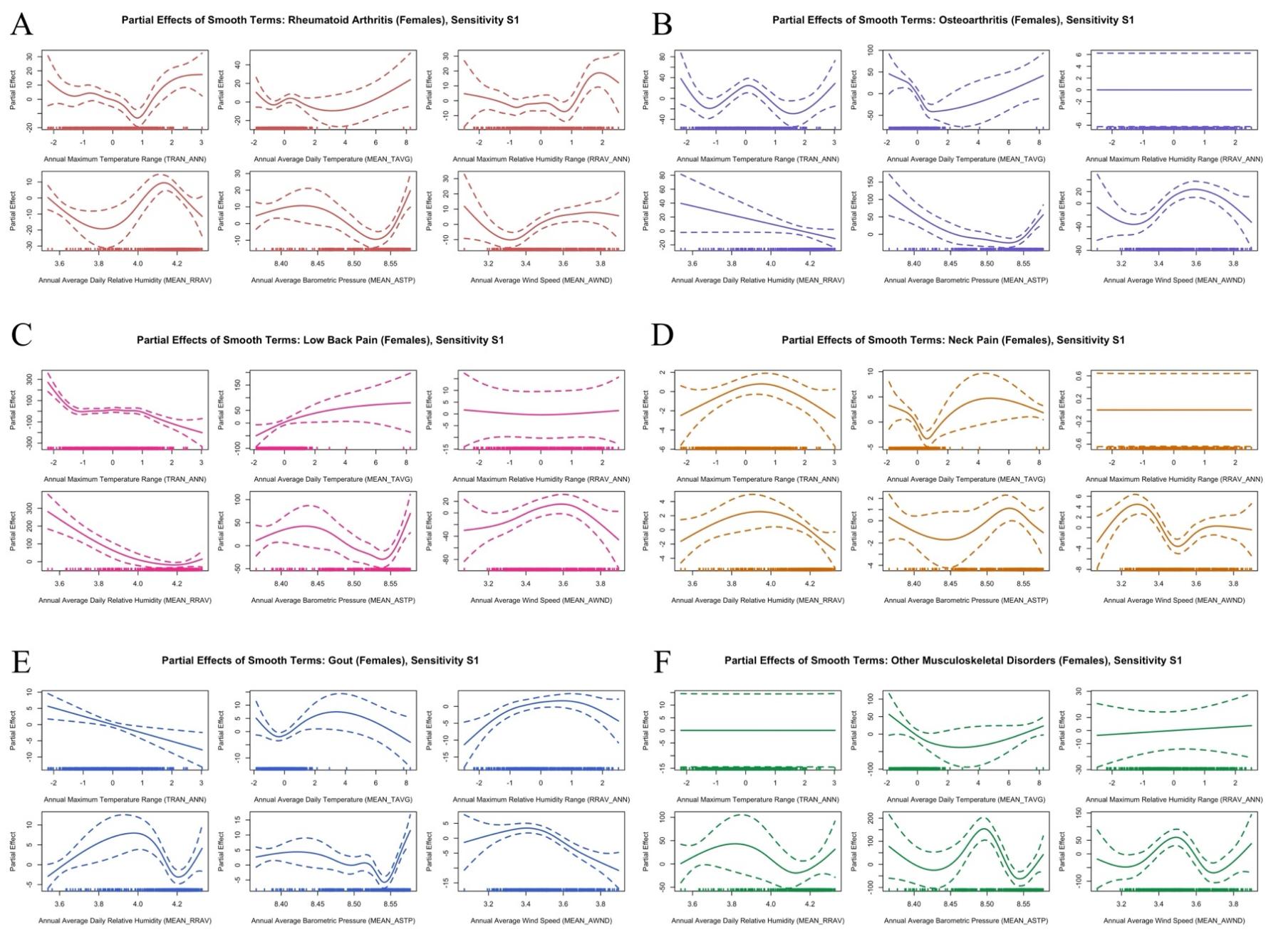


**Supplementary Material Figure 9.** **Partial effects of GAM smooth terms (Sensitivity Model S1) for females**

**(A: rheumatoid arthritis; B: osteoarthritis; C: low back pain; D: neck pain; E: gout; F: other musculoskeletal disorders).**

TRAN_ANN: Annual maximum temperature range; MEAN_TAVG: Annual average daily temperature; RRAV_ANN: Annual relative humidity range; MEAN_RRAV: Annual average daily relative humidity; MEAN_ASTP: Annual average barometric pressure; MEAN_AWND: Annual average wind speed


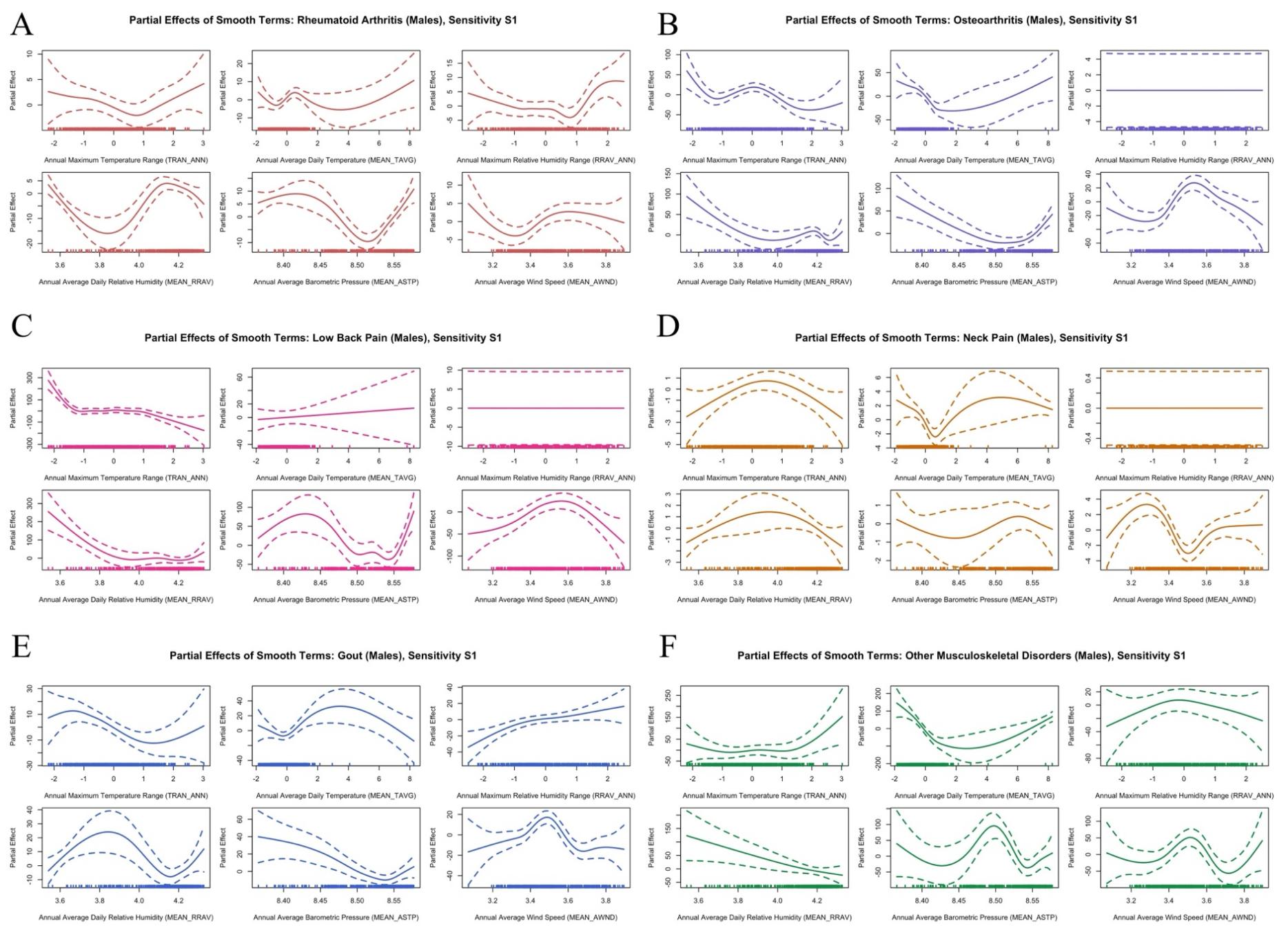


**Supplementary Material Figure 10.** **Partial effects of GAM smooth terms (Sensitivity Model S1) for males**

**(A: rheumatoid arthritis; B: osteoarthritis; C: low back pain; D: neck pain; E: gout; F: other musculoskeletal disorders).**

TRAN_ANN: Annual maximum temperature range; MEAN_TAVG: Annual average daily temperature; RRAV_ANN: Annual relative humidity range; MEAN_RRAV: Annual average daily relative humidity; MEAN_ASTP: Annual average barometric pressure; MEAN_AWND: Annual average wind speed


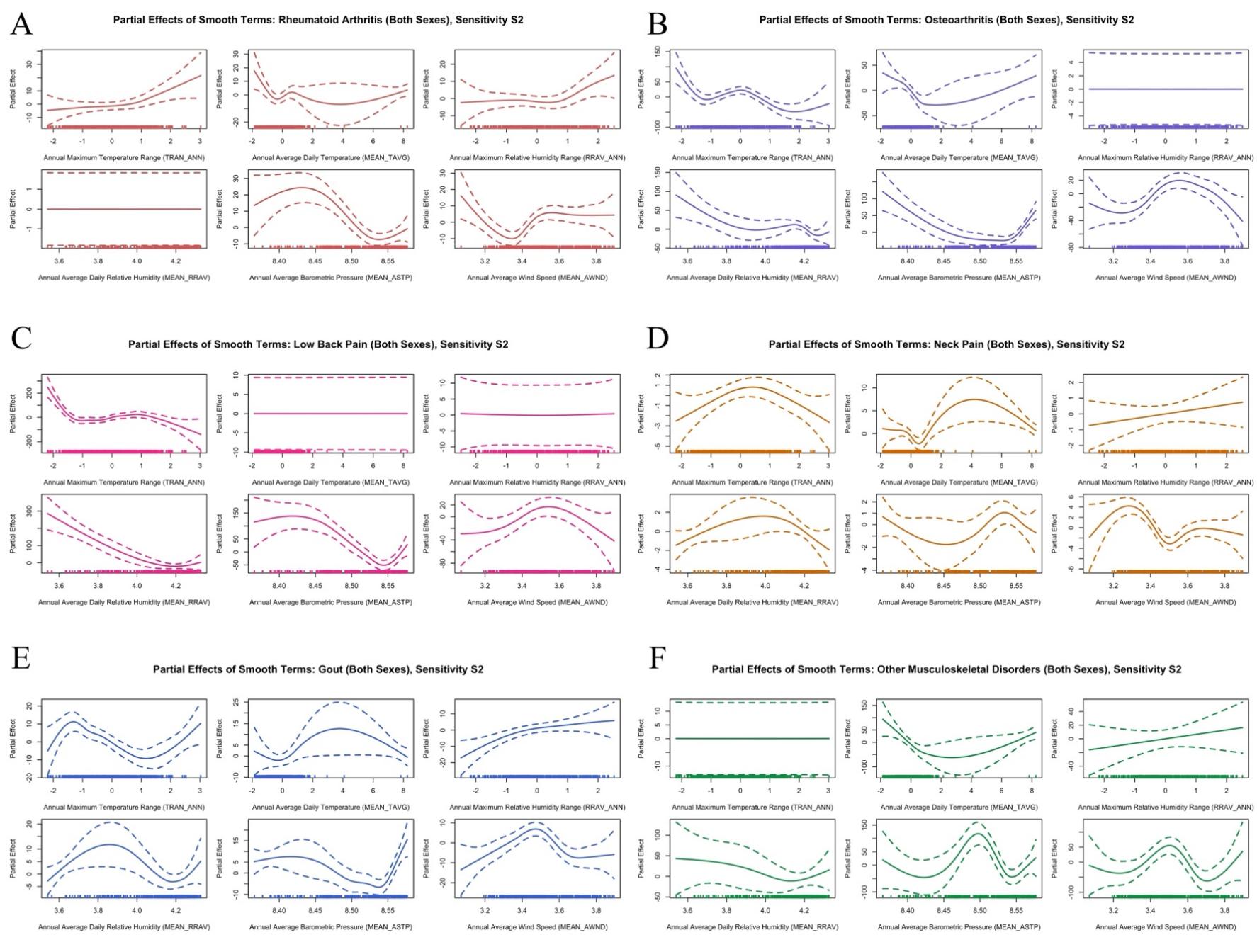


**Supplementary Material Figure 11.** **Partial effects of GAM smooth terms (Sensitivity Model S2) for both sexes**

**(A: rheumatoid arthritis; B: osteoarthritis; C: low back pain; D: neck pain; E: gout; F: other musculoskeletal disorders).**

TRAN_ANN: Annual maximum temperature range; MEAN_TAVG: Annual average daily temperature; RRAV_ANN: Annual relative humidity range; MEAN_RRAV: Annual average daily relative humidity; MEAN_ASTP: Annual average barometric pressure; MEAN_AWND: Annual average wind speed


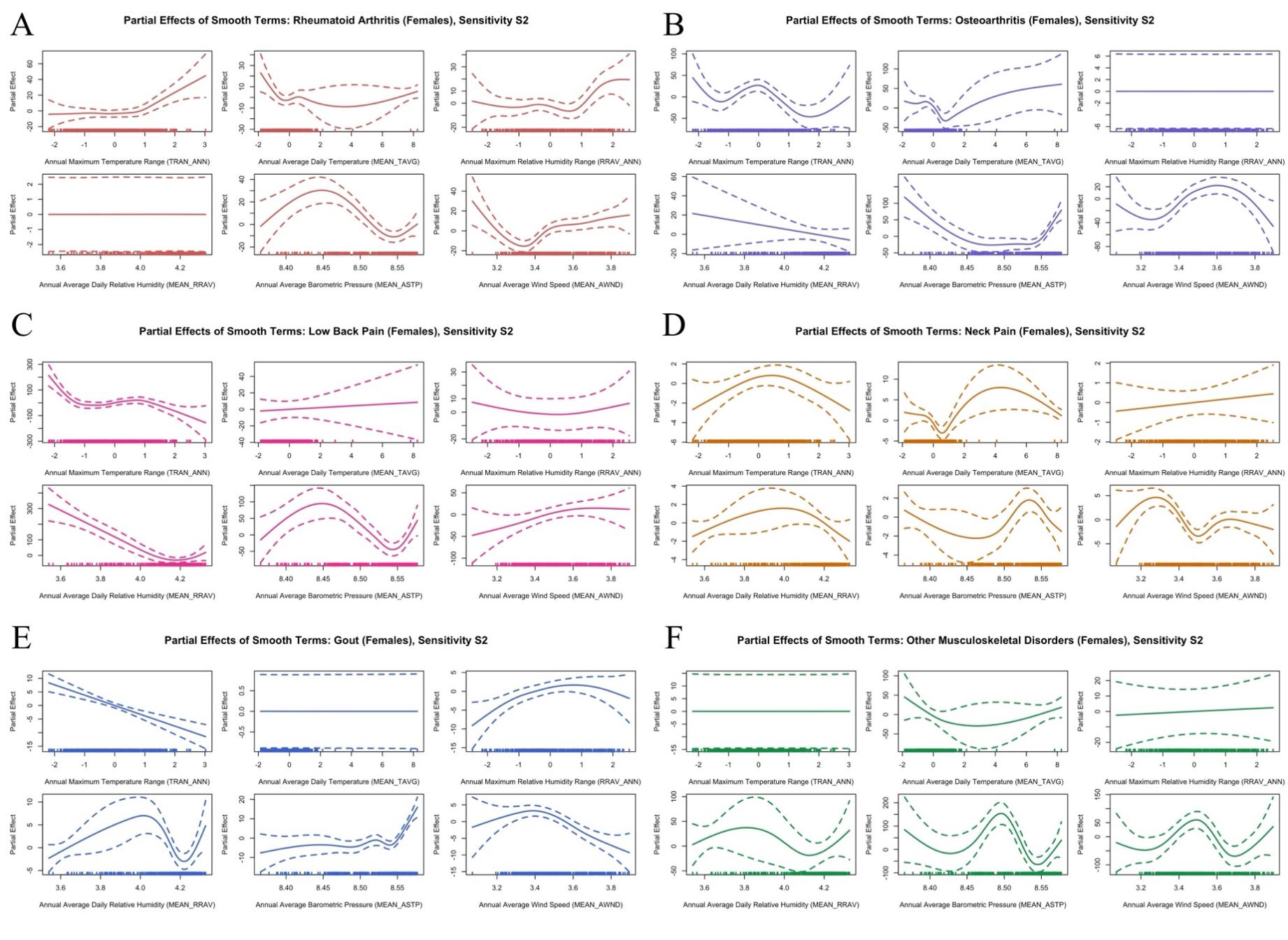


**Supplementary Material Figure 12.** **Partial effects of GAM smooth terms (Sensitivity Model S2) for females**

**(A: rheumatoid arthritis; B: osteoarthritis; C: low back pain; D: neck pain; E: gout; F: other musculoskeletal disorders).**

TRAN_ANN: Annual maximum temperature range; MEAN_TAVG: Annual average daily temperature; RRAV_ANN: Annual relative humidity range; MEAN_RRAV: Annual average daily relative humidity; MEAN_ASTP: Annual average barometric pressure; MEAN_AWND: Annual average wind speed


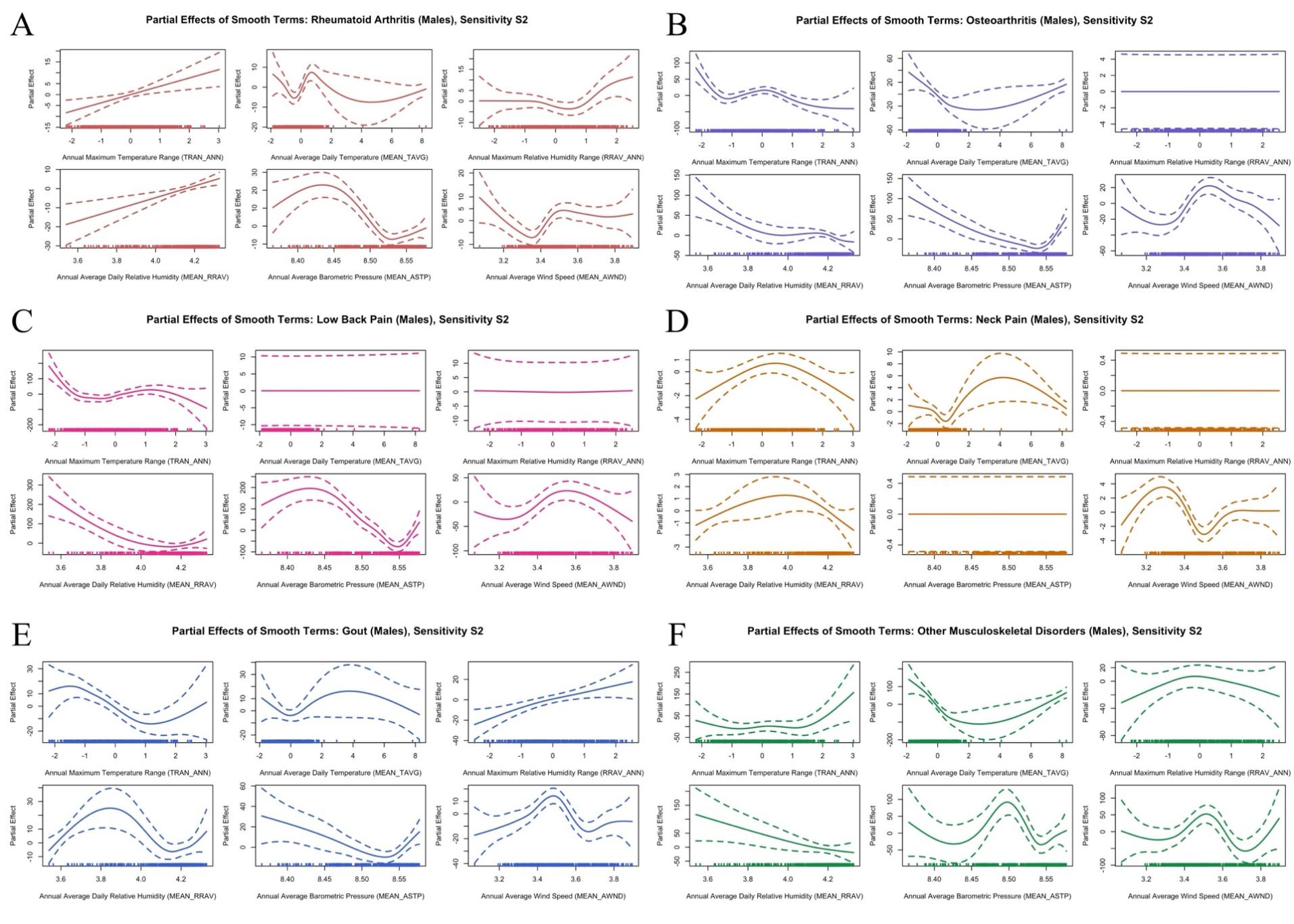


**Supplementary Material Figure 13.** **Partial effects of GAM smooth terms (Sensitivity Model S2) for males**

**(A: rheumatoid arthritis; B: osteoarthritis; C: low back pain; D: neck pain; E: gout; F: other musculoskeletal disorders).**

TRAN_ANN: Annual maximum temperature range; MEAN_TAVG: Annual average daily temperature; RRAV_ANN: Annual relative humidity range; MEAN_RRAV: Annual average daily relative humidity; MEAN_ASTP: Annual average barometric pressure; MEAN_AWND: Annual average wind speed.

**
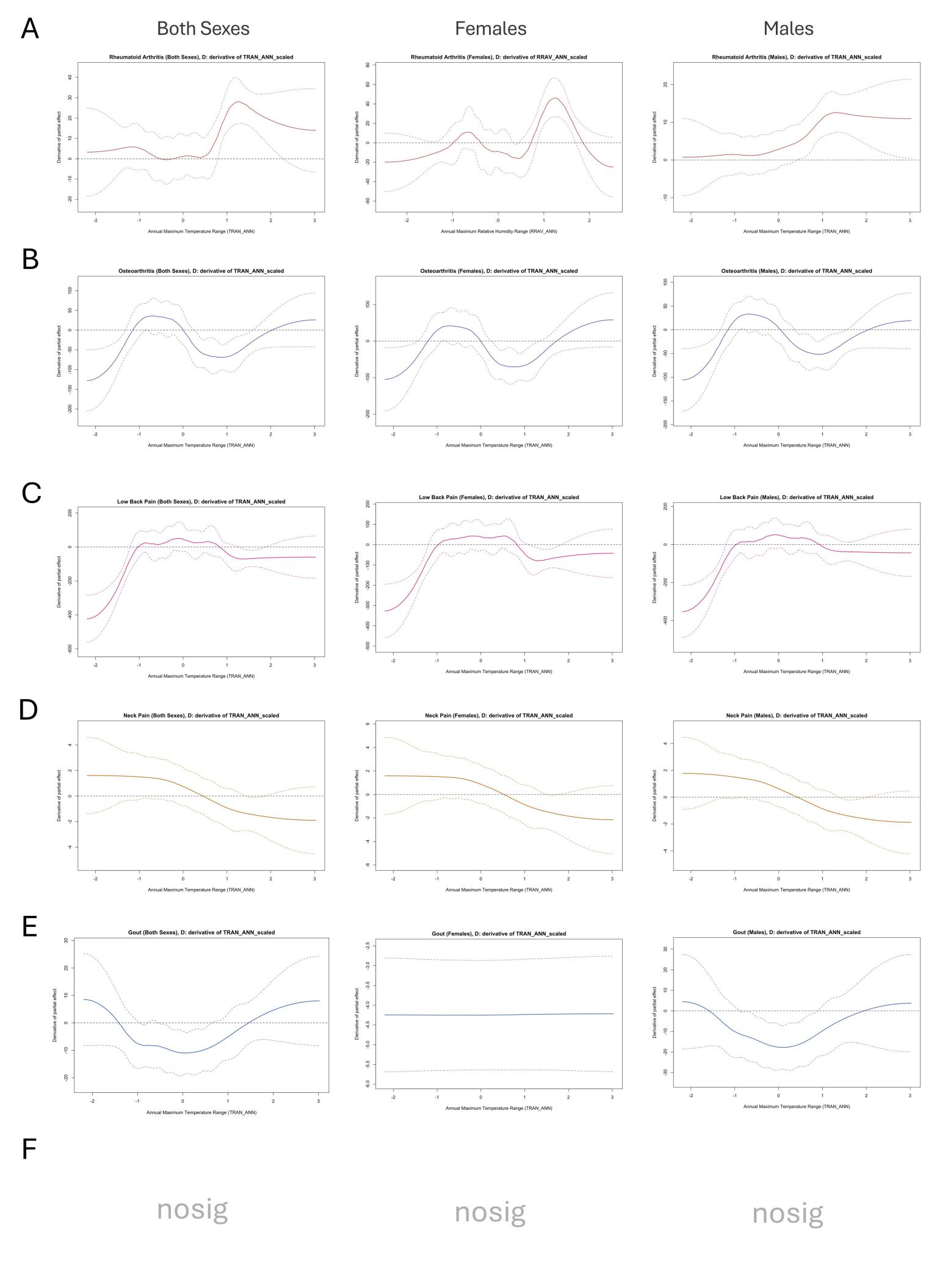
**

**Supplementary Material Figure 14. First derivatives of GAM smooth terms for Annual maximum temperature range (TRAN_ANN; nosig: no statistical significance at 0.05 level).** A: rheumatoid arthritis; B: osteoarthritis; C: low back pain; D: neck pain; E: gout; F: other musculoskeletal disorders.


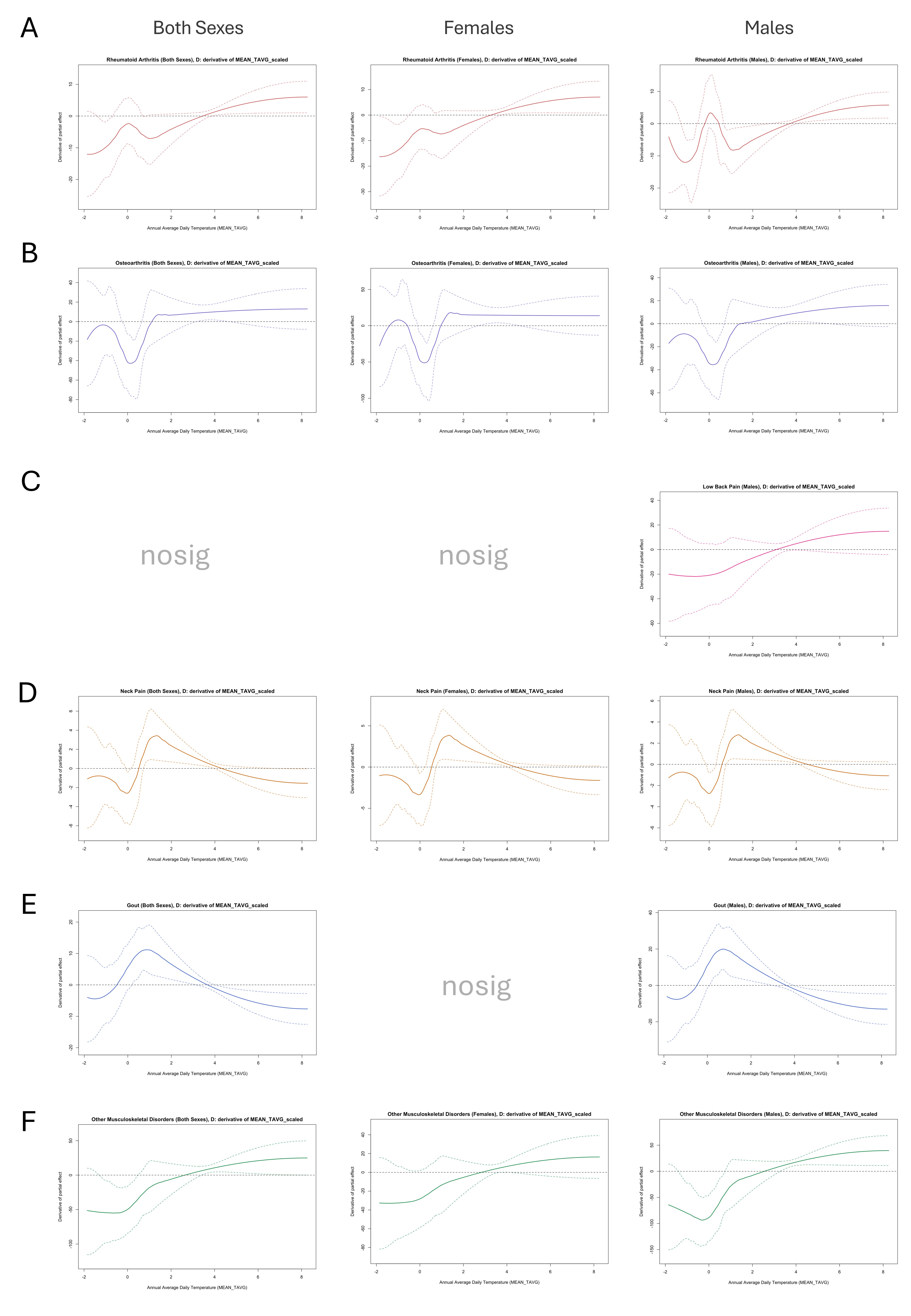


**Supplementary Material Figure 15. First derivatives of GAM smooth terms for Annual average daily temperature (MEAN_TAVG; nosig: no statistical significance at 0.05 level).** A: rheumatoid arthritis; B: osteoarthritis; C: low back pain; D: neck pain; E: gout; F: other musculoskeletal disorders.


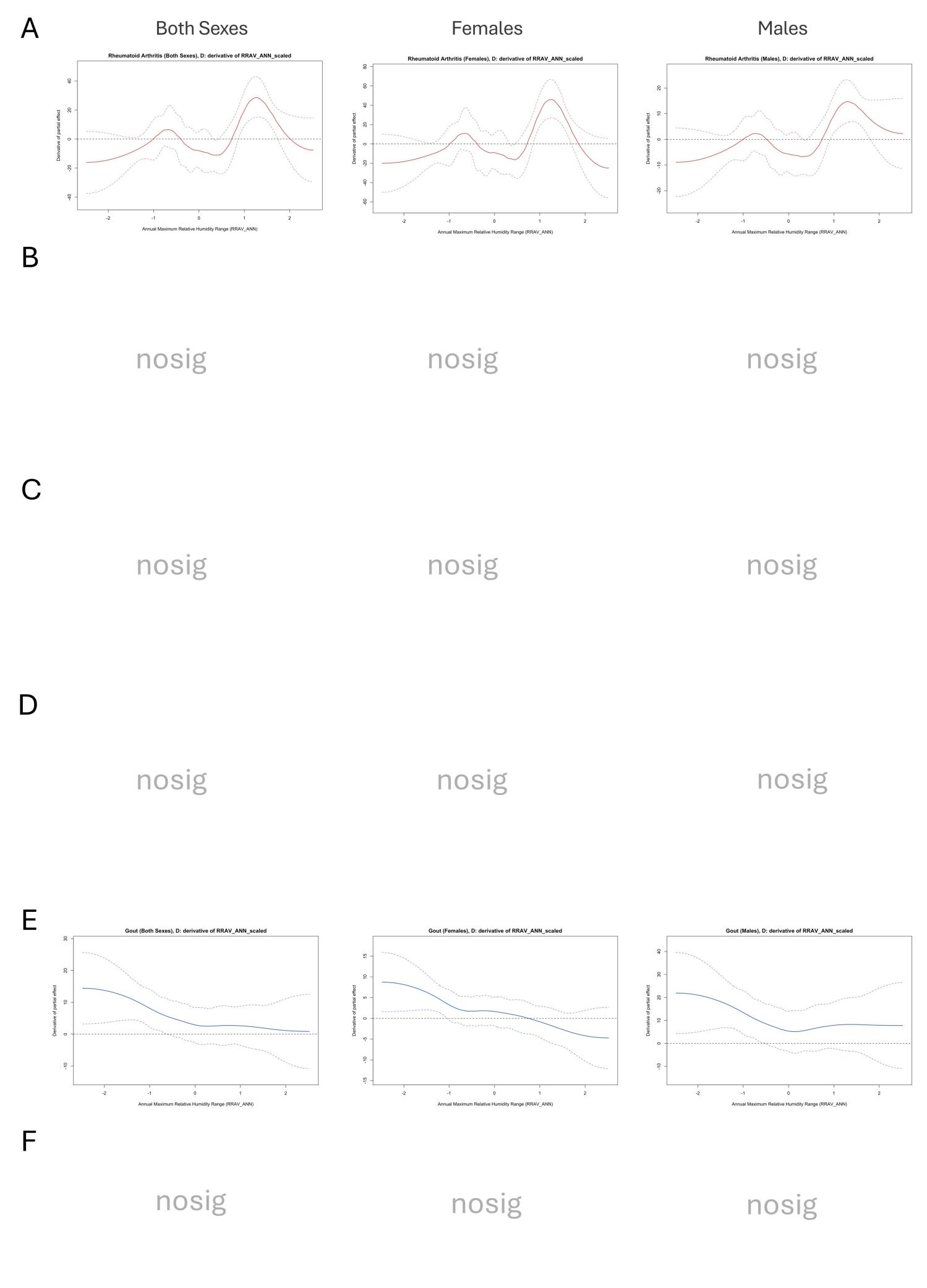


**Supplementary Material Figure 16. First derivatives of GAM smooth terms for Annual relative humidity range (RRAV_ANN; nosig: no statistical significance at 0.05 level).** A: rheumatoid arthritis; B: osteoarthritis; C: low back pain; D: neck pain; E: gout; F: other musculoskeletal disorders.


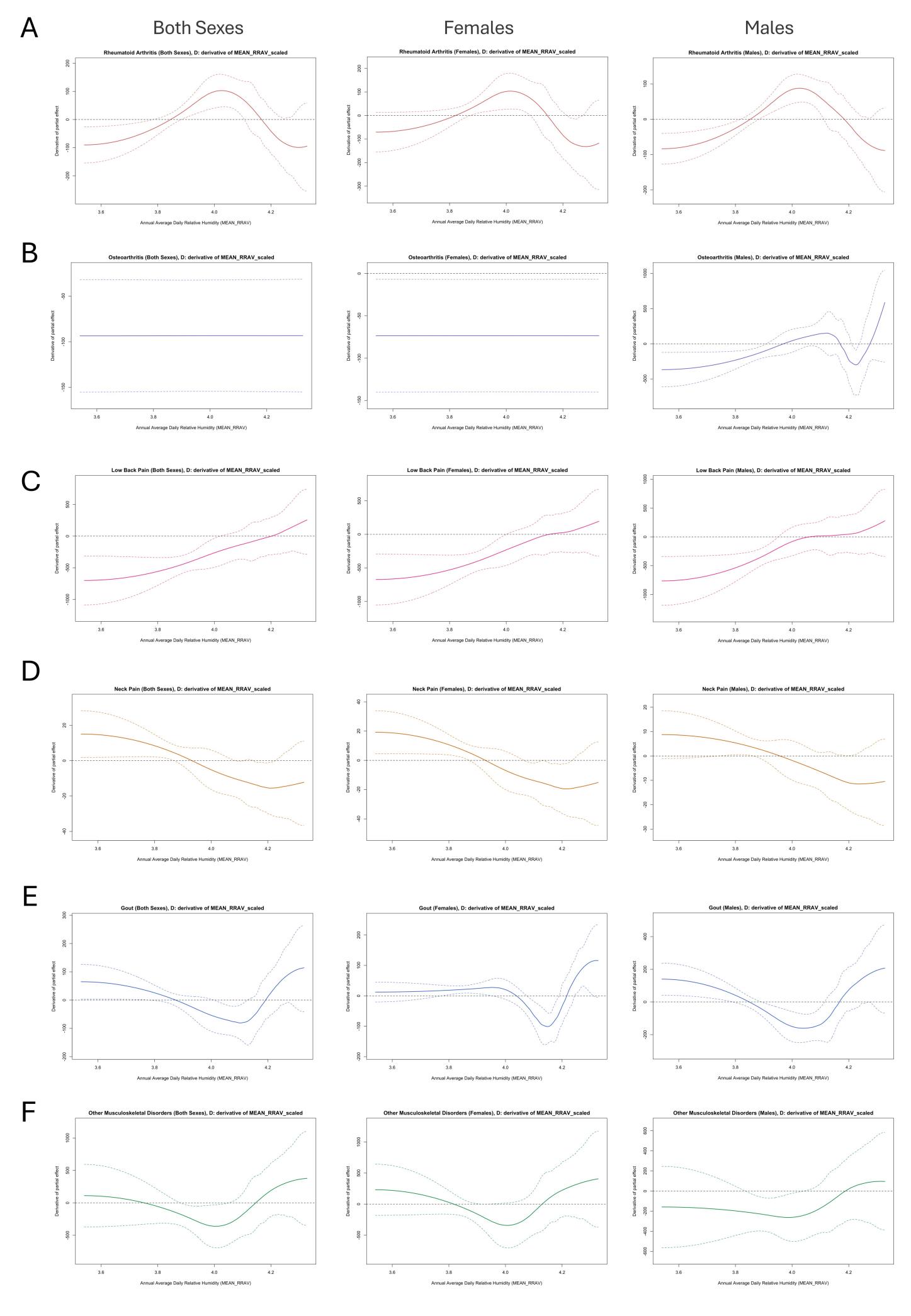


**Supplementary Material Figure 17. First derivatives of GAM smooth terms for Annual average daily relative humidity (MEAN_RRAV; nosig: no statistical significance at 0.05 level).** A: rheumatoid arthritis; B: osteoarthritis; C: low back pain; D: neck pain; E: gout; F: other musculoskeletal disorders.


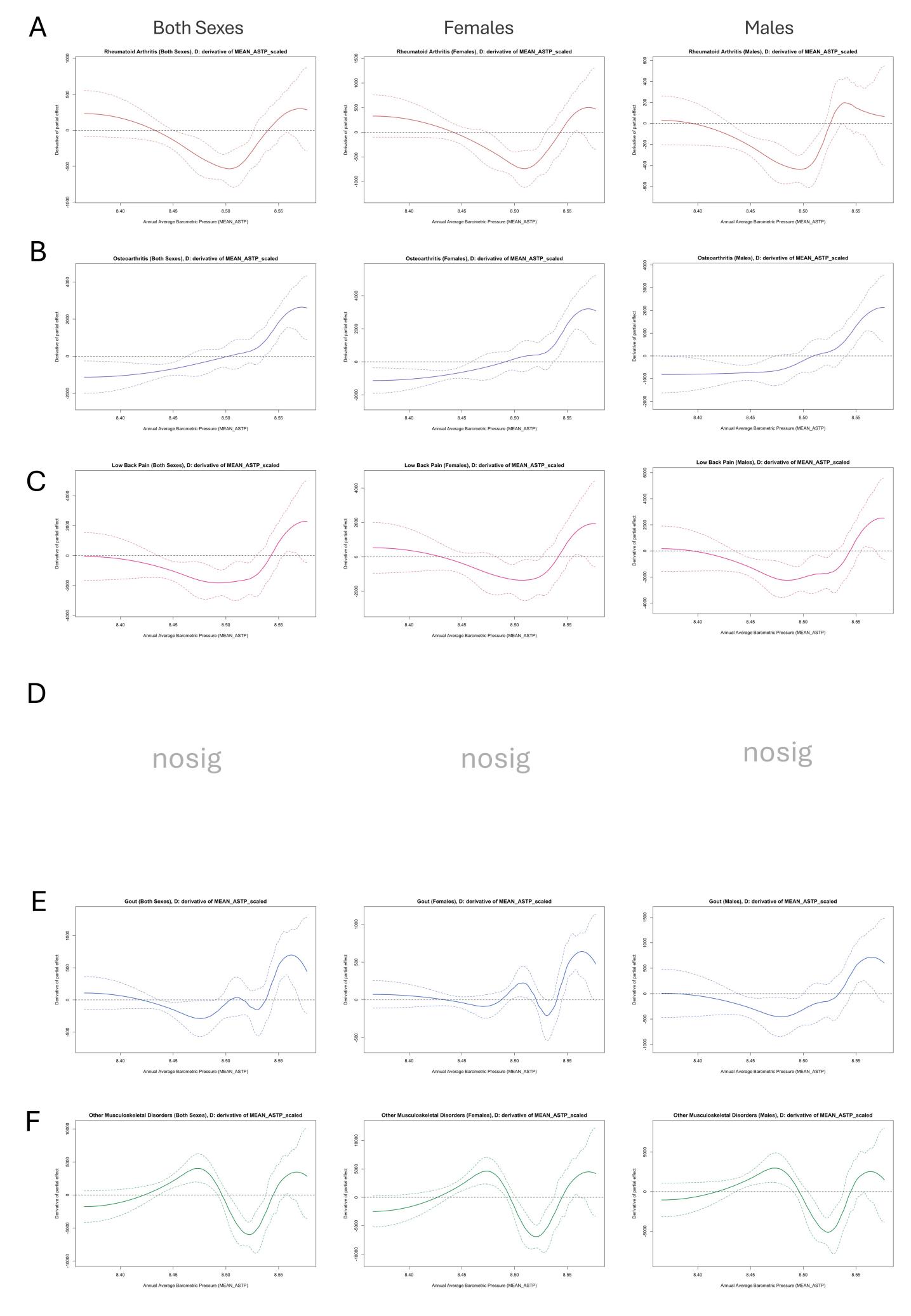


**Supplementary Material Figure 18. First derivatives of GAM smooth terms for Annual average barometric pressure (MEAN_ASTP; nosig: no statistical significance at 0.05 level).** A: rheumatoid arthritis; B: osteoarthritis; C: low back pain; D: neck pain; E: gout; F: other musculoskeletal disorders.


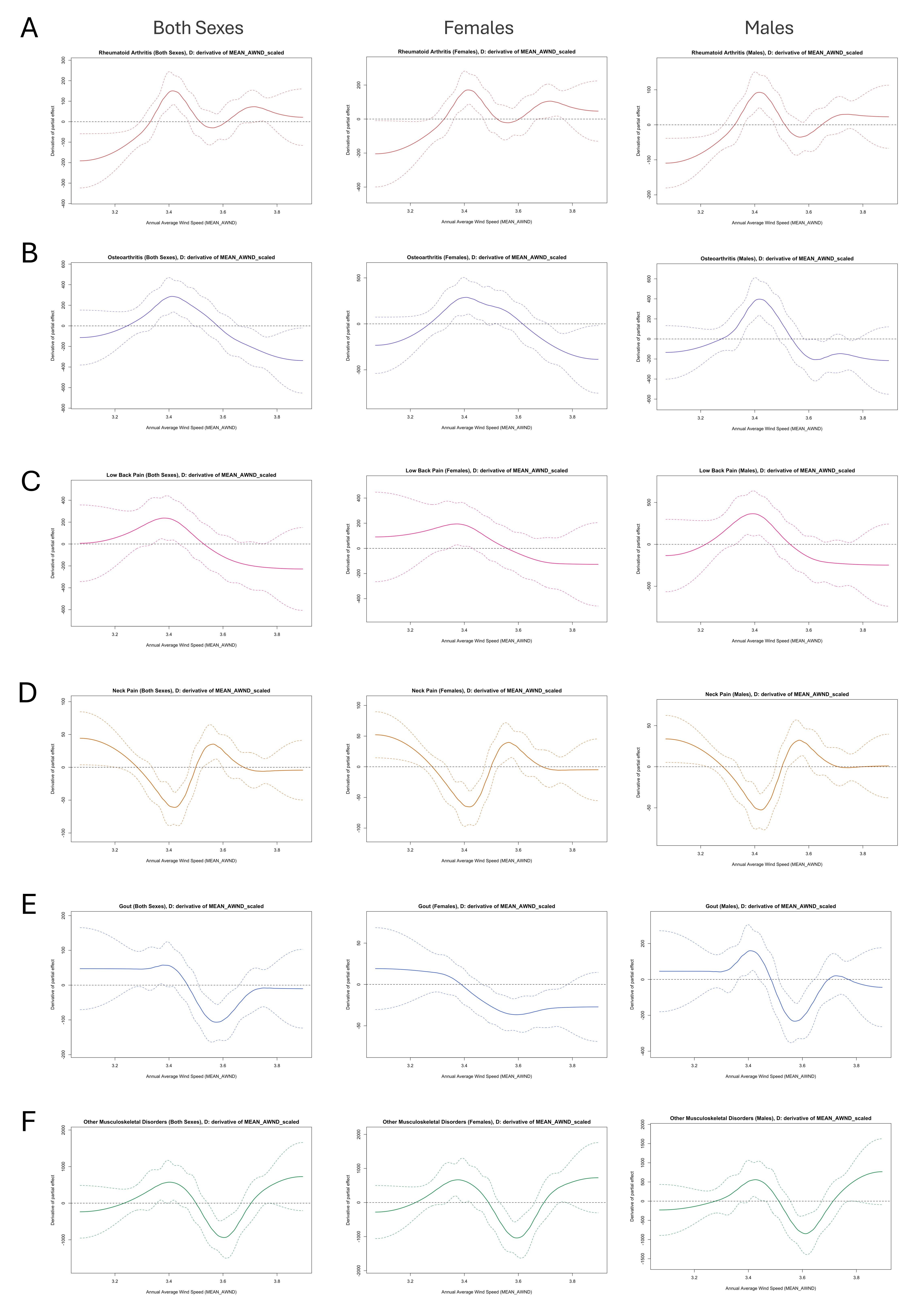
**Supplementary Material Figure 19. First derivatives of GAM smooth terms for Annual average wind speed (MEAN_AWND; nosig: no statistical significance at 0.05 level).** A: rheumatoid arthritis; B: osteoarthritis; C: low back pain; D: neck pain; E: gout; F: other musculoskeletal disorders.

**Reference**

1. Devleesschauwer B, Charalampous P, Gorasso V, Assunção R, Hilderink H, Idavain J, et al. Standardised reporting of burden of disease studies: the STROBOD statement. Population Health Metrics. 2024;22(1):28.

2. Jin Y, Guo C, Abbasian M, Abbasifard M, Abbott JH, Abdullahi A, et al. Global pattern, trend, and cross-country inequality of early musculoskeletal disorders from 1990 to 2019, with projection from 2020 to 2050. Med. 2024;5(8):943-62.e6.

3. Global, regional, and national burden of other musculoskeletal disorders, 1990-2020, and projections to 2050: a systematic analysis of the Global Burden of Disease Study 2021. Lancet Rheumatol. 2023;5(11):e670-e82.

4. Menne MJ, Durre I, Vose RS, Gleason BE, Houston TG. An Overview of the Global Historical Climatology Network-Daily Database. Journal of Atmospheric and Oceanic Technology. 2012;29(7):897-910.
